# Contribution of Amino Acid Catabolism to the Tissue Specific Persistence of *Campylobacter jejuni* in a Murine Colonization Model

**DOI:** 10.1371/journal.pone.0050699

**Published:** 2012-11-30

**Authors:** Dirk Hofreuter, Juliane Mohr, Olga Wensel, Sebastian Rademacher, Kerstin Schreiber, Dietmar Schomburg, Beile Gao, Jorge E. Galán

**Affiliations:** 1 Institute for Medical Microbiology, Hannover Medical School, Hannover, Germany; 2 Institute for Biochemistry, Biotechnology and Bioinformatics, Technische Universität Braunschweig, Braunschweig, Germany; 3 Section of Microbial Pathogenesis, Yale University School of Medicine, New Haven, Connecticut, United States of America; Institut Pasteur Paris, France

## Abstract

*Campylobacter jejuni* is a major cause of food-borne disease in industrialized countries. Carbohydrate utilization by *C. jejuni* is severely restricted, and knowledge about which substrates fuel *C. jejuni* infection and growth is limited. Some amino acids have been shown to serve as carbon sources both *in vitro* and *in vivo*. In the present study we investigated the contribution of serine and proline catabolism to the *in* *vitro* and *in* *vivo* growth of *C*. *jejuni* 81-176. We confirmed that the serine transporter SdaC and the serine ammonia-lyase SdaA are required for serine utilization, and demonstrated that a predicted proline permease PutP and a bifunctional proline/delta-1-pyrroline-5-carboxylate dehydrogenase PutA are required for proline utilization by *C. jejuni* 81-176. *C. jejuni* 81-176 mutants unable to utilize serine were shown to be severely defective for colonization of the intestine and systemic tissues in a mouse model of infection. In contrast, *C. jejuni* 81-176 mutants unable to utilize proline were only defective for intestinal colonization. These results further emphasize the importance of amino acid utilization in *C. jejuni* colonization of various tissues.

## Introduction

Although knowledge of bacterial metabolism during growth *in vitro* is substantial, much less is known about the metabolism of bacterial pathogens during infection [1], [2]. The understanding of the *in vivo* metabolism of pathogens has gained importance in recent years as it may facilitate the development of novel antimicrobial drugs [3], [4]. *Campylobacter jejuni*, a gram-negative, microaerophilic and chemoorganotrophic bacterium, is a major cause of food-borne diarrhea worldwide [5]. *C. jejuni* efficiently colonizes the intestinal tract of birds, especially poultry, without any adverse effects [6]–[8]. In contrast, infections of humans can lead to severe diarrheal illness [9], [10] and in rare cases to an acute polyneuropathy, the Guillain-Barré Syndrome, as sequelae to the foodborne disease [11]. Relatively little is known about the molecular mechanisms of *C. jejuni* pathogenesis [12], [13]. Previous studies suggest that adherence to as well as invasion into intestinal epithelial cells and the production of cytolethal distending toxin (CDT) contribute to the pathogenesis of *C. jejuni* [14]–[18]. Furthermore, motility and chemotaxis [19]–[22], a N-gylcosylation system as well as surface structures like lipooligosaccharide, capsule and the O-glycosylated flagella have been shown to participate in the experimental infection of animals [20], [23]–[30]. Some studies have also begun to define the metabolic requirements for *C*. *jejuni* growth *in vitro* [31], [32]. Unlike other enteropathogenic bacteria, *C. jejuni* cannot utilize glucose as a growth substrate since it lacks the glycolytic enzyme phosphofructokinase of the Embden-Meyerhof-Parnas (EMP) pathway [33]–[35]. Furthermore, *C*. *jejuni* does not encode a complete pentose phosphate (PPP) or Entner-Doudoroff (ED) pathway [33], [34]. *C. jejuni* has been considered asaccharolytic, but recent studies demonstrated that a subset of *C. jejuni* strains encode a gene cluster required for the utilization of fucose as an energy source [36], [37]. Instead of glucose and other carbohydrates *C. jejuni* can utilize several glucogenic amino acids as efficient growth substrates [38]–[42], although there are intriguing strain differences in the ability to utilize specific amino acids [43]. In addition to glucogenic amino acids, the TCA cycle intermediates fumarate, malate, succinate and α-ketoglutarate [44], as well as pyruvate [45], [46] and lactate [35], [47] can serve as growth-promoting substrates for various *C. jejuni* strains *in vitro*.

The metabolic requirements for *C. jejuni* colonization in different animal model systems are being defined, although only few metabolic traits have so far been demonstrated to be important for infection. As for many other pathogens, zinc and iron acquisition plays an important role in *C. jejuni* colonization and mutations in zinc, ferrous as well as ferric iron uptake systems reduced the ability of *C. jejuni* to colonize the chicken intestine [48]–[50]. Its ability to carry out oxygen-independent respiration mediated by a gluconate dehydrogenase and nitrate, nitrite, fumarate as well as dimethylsulfoxide reductases are also required for efficient colonization [51]–[54]. While it is well documented that amino acids represent major energy and carbon sources for the *in vitro* growth of *C. jejuni*, only a few studies have examined the contribution of amino acid catabolism to bacterial colonization. For example, serine and aspartate catabolism were shown to be important for *C. jejuni* colonization of the avian gut [38], [45] and mutation of a putative branched amino acid transporter resulted in an attenuated colonization phenotype in chicken [20], [55]. The γ-glutamyltranspeptidase (GGT) dependent utilization of glutamine and glutathione by *C. jejuni* 81-176 supports the colonization of the murine intestine [51]. In addition, asparagine catabolism enhances the tissue-specific colonization process of *C. jejuni* 81-176 in a murine infection model [43]. Here we show that the ability to utilize the glucogenic amino acids serine and proline is also important for the colonization of specific tissues by *C. jejuni*.

## Results

### Serine Catabolism is a Variable Metabolic Trait in *Campylobacter*

**Figure 1:**
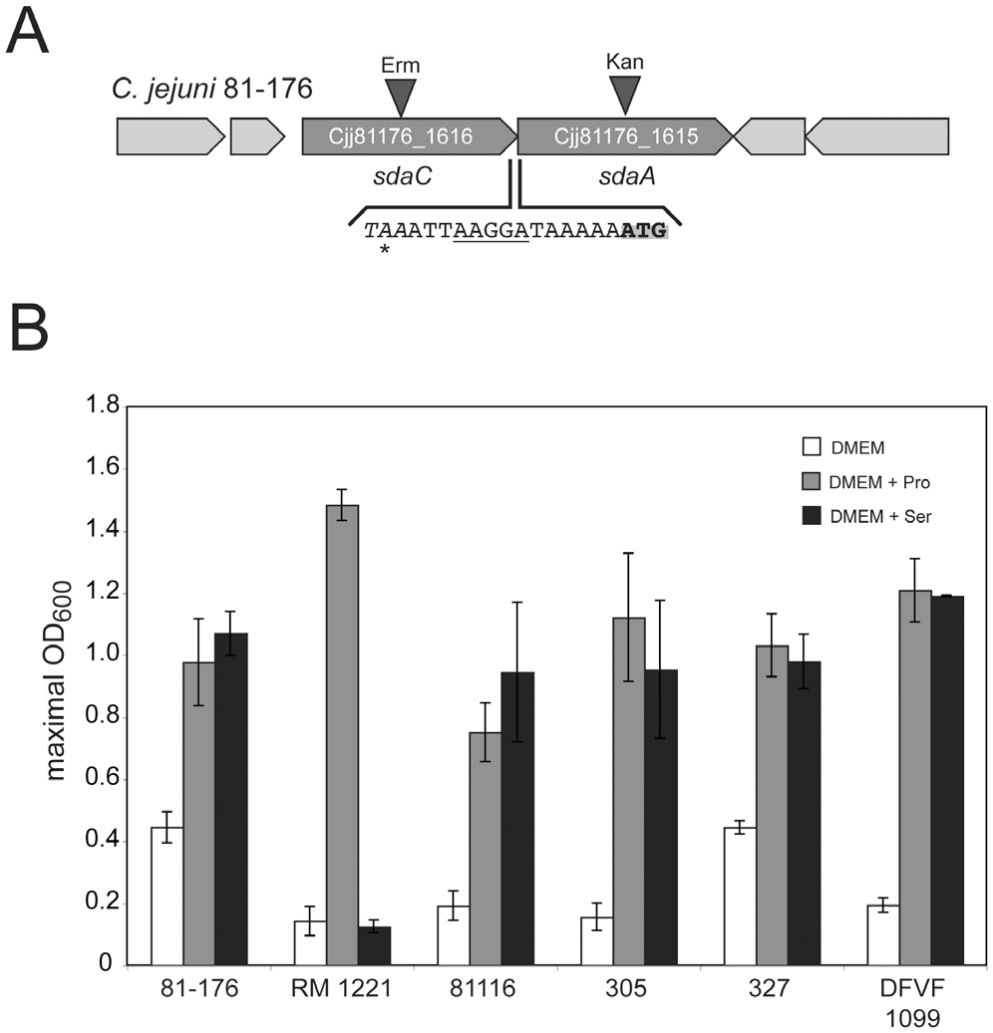
Serine utilization locus in *C. jejuni.* (A) The serine utilization locus of *C. jejuni* consists of the s*daCA* operon, which is conserved in all sequenced *C. jejuni* strains. The genes *sdaC* and *sdaA* encode for a serine uptake transporter and a L-serine dehydratase, respectively. The intergenic sequence is shown with the *sdaA* start codon (ATG), the *sdaA* shine-dalgarno squence (underlined) and the *sdaC* stop codon (*). Mutants of *sdaA* and *sdaC* were constructed by insertion of kanamycin and erythromycin resistance cassettes in respective genes as indicated. (B) Comparison of different *C. jejuni* wild-type isolates to utilize serine as growth substrate. The shown optical densities are the mean values ±SD that were maximally reached by indicated wild-type *C. jejuni* strains RM1221, 81116, 305, 327 and DFVF1099 during a growth period of 48 hours in DMEM supplemented with 20 mM of the indicated amino acids.

**Figure 2:**
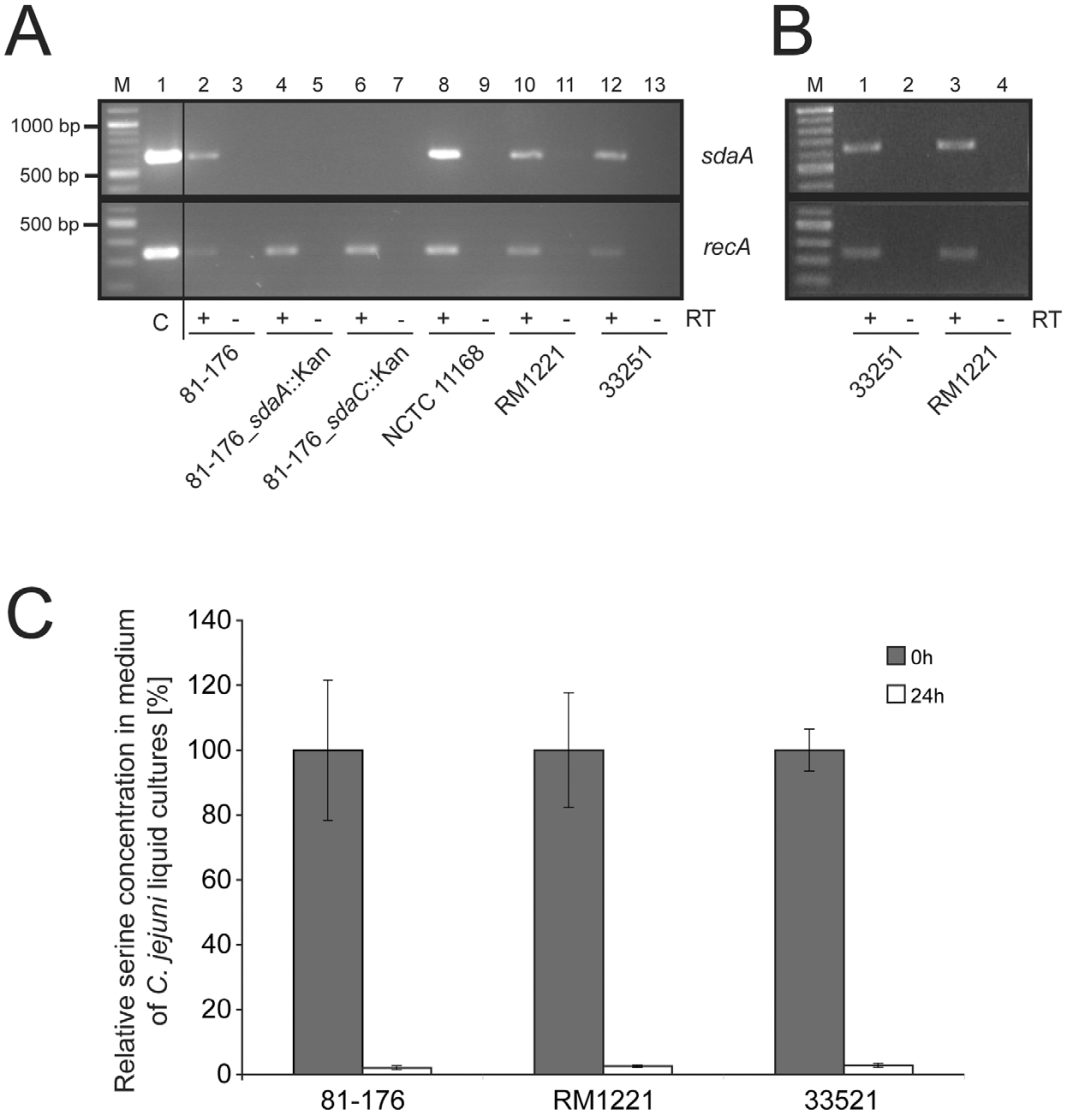
Comparison of *sdaA* expression and serine uptake in different *C. jejuni* isolates. The expression of the s*daCA* operon in various wild-type *C. jejuni* strains as well as the *sdaA* and *sdaC* mutants of *C. jejuni* 81-176 cultivated in BHI medium (A) or DMEM (B) was examined by reverse transcriptase (RT)-PCR analysis using *sdaA* specific primer (upper panel). RT-PCRs with *recA* specific primers are shown in the lower panel for comparison. To verify the absence of chromosomal DNA contamination in the RNA preparations, PCRs were performed using RNA samples without RT treatment (- RT) as templates. PCRs with respective *sdaA* and recA primers and chromosomal DNA of *C. jejuni* 81-176 served as positive controls (A, lane 1, upper and lower panel). (C) The capability of *C. jejuni* isolates 81-176, RM1221 and 33251 to take up serine from the medium was measured by monitoring the relative changes of serine concentration in the culture supernatant over a cultivation period of 24 hours by GC-MS analysis. The bacteria were grown in Hanks Balance Salt Solution (HBSS) with 1% casein hydrolysate as amino acid sources and supplemented with vitamins and iron. The relative serine concentration at the beginning of the experiment was set as 100%. The mean values ±SD are the results of two independent experiments measured in triplicates.

It was previously shown, that serine serves as an efficient nutrient for the *in vitro* growth of various *C. jejuni* isolates [39], [42], [45]. Serine utilization in *C. jejuni* is mediated by the L-serine dehydratase SdaA and the serine transporter SdaC [45] as described for other bacteria. Our examination of published [33], [34], [51], [56]–[64] and unpublished *C. jejuni* genome sequences revealed a high conservation of the genes encoding for SdaA and SdaC in *C jejuni* (Figure 1A, Figures S1 and S2, Tables S1 and S2). Strikingly, we recently identified *C. jejuni* 33251 as the only strain of 13 isolates tested that was unable to utilize serine [43]. This was surprising considering the observed conservation of *sdaA* and *sdaC* among *C. jejuni* isolates. To understand the inability of *C. jejuni* 33251 to utilize serine, we sequenced its *sdaCA* locus. We identified only few nucleotide differences in the *sdaA* and sdaC genes that do not result in the inactivation of respective open reading frames of this isolate. In addition, we found a small insertion leading to 3 additional amino acids in the coding sequence of SdaA (Figures S1, S2, S3 and S4). We confirmed that mutations of *sdaA* and/or *sdaC* are not necessarily responsible for the abolished serine catabolism in certain *C. jejuni* strains by analyzing the sequenced *C. jejuni* isolates RM1221, 81116, 305, 327 and DFVF1099. While these five strains encode for nearly identical *sdaA* and *sdaC* gene products (Figures S3 and S4), *C. jejuni* RM1221 was, like *C. jejuni* 33251, unable to grow with serine as sole energy source, whereas the *C. jejuni* 81116, 305, 327 and DFVF1099 catabolized serine efficiently (Figure 1B). We hypothesized that reduced expression of the *sdaCA* operon could be responsible for the inability of *C. jejuni* 33251 and RM1221 to grow with serine. To examine this, mRNA from the strains *C. jejuni* 81-176, NCTC 11168, 33251 and RM1221 was isolated and reverse transcriptase-PCR analysis of *sdaA* was used to elucidate if differences in the expression between the wild-type strains occur. Interestingly, the serine utilizing and non-utilizing *C. jejuni* strains showed similar *sdaA* expression as *C. jejuni* 81-176 and NCTC 11168 when cultivated in nutrient rich BHI medium (Figure 2A) or in DMEM (Figure 2B). To clarify if differences in the uptake of serine by the different *C. jejuni* isolates was causing the defective growth phenotypes of *C. jejuni* RM1221 and 33251, we analyzed the decrease of serine concentration in the growth medium with GC-MS analysis. For all three tested *C. jejuni* isolates efficient serine uptake was observed, as the amount of serine in the culture medium vanished to a similar extent over the time period of 24 hours (Figure 2C). This suggested that other strain-specific characteristics are accountable for the inability of *C. jejuni* 33251 and RM1221 to catabolize serine. We suspected that perhaps dissimilarities in the L-serine dehydratase activity of individual *C. jejuni* isolates could be responsible for their variable growth with serine. Using the enzymatic assay previously described for the measurement of the SdaA activity in *C. jejuni* NCTC 11168 [45], we detected in the cell extracts of *C. jejuni 33251* and RM1221 a small but significant reduction of L-serine dehydratase activity in comparison to *C. jejuni* 81-176 (Table S5). Taken together our data suggest that neither differences in the *sdaCA* gene expression nor serine uptake are responsible for the inability of *C. jejuni* 33251 and RM1221 to grow this serine, but rather a reduced serine deyhdratase activity or other yet not identified factors.

### Serine Catabolism is Required for Efficient *C. jejuni* 81-176 Mouse Colonization

It has previously been demonstrated that inactivation of the *sdaA* gene in *C. jejuni* NCTC 11168 resulted in reduced persistence in the intestine of infected chicken [45]. Here we examined whether the serine catabolism of *C. jejuni* 81-176 provides an advantage for its colonization of different tissues in a murine infection model [30]. As predicted from studies in *C. jejuni* strain NCTC 11168 [45], inactivation of the *sdaA* or *sdaC* genes in *C. jejuni* 81-176 abolished its ability to utilize serine as a carbon source (Figure S5). Both mutants exhibited comparable growth to the wild type strain when cultivated in DMEM supplemented with other amino acids. Furthermore, the phenotype of the *sdaA* mutant was reversed by the reintroduction of a copy of the wild-type *sdaA* gene.

**Figure 3:**
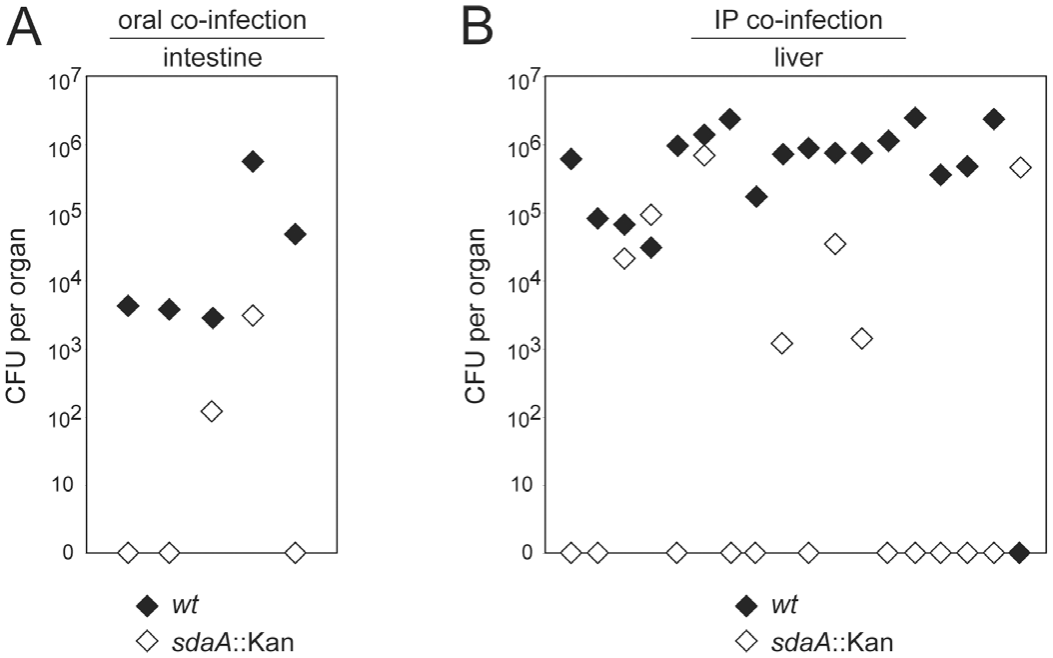
Role of serine catabolism in *C. jejuni* 81-176 mouse colonization. Shown is the colonization efficiency of *C. jejuni* 81-176 wild-type (black diamonds) and its isogenic *sdaA* mutant strain (white diamonds) in a murine model of infection. The colonization of the intestine (A) and liver (B) was evaluated 5 weeks after oral or intraperitoneal co-infection of myd88^−/−^ nramp^−/−^ mice, respectively. Each pair of black and white diamonds represents the CFU numbers of the *C. jejuni* 81-176 wild-type strain and its *sdaA* mutant recovered from the indicated organs of an individual animal. The combined results of one (A) and two (B) independent infection experiments are shown. The Mann–Whitney U test ( = *Wilcoxon rank-sum test)* was used to calculate the P values for the statistical differences between the CFU numbers of the different strains: P<0.05 (A) and P<0.001 (B).

To investigate how serine catabolism affects *C. jejuni* 81-176 colonization and persistence in the murine infection model, we inoculated *myd88* ^_^/^_^ C57BL/6 mice orally or intraperitoneally with equal numbers of wild-type *C. jejuni* 81-176 and its isogenic *sdaA* mutant. Five weeks after infection animals were sacrificed and the colony forming units (CFUs) of *C. jejuni* in intestine and liver were determined (Figure 3). High CFUs numbers of the wild-type *C. jejuni* strain could be recovered from the intestine of orally infected mice. In contrast, the *sdaA* mutant had a reduced capacity to persist in the murine intestine (Figure 3A, Figure S6A). In addition, the *sdaA* mutant could not be recovered from the liver of most animals (11 out of 18) or was recovered in significantly reduced numbers in comparison to the wild-type strain (Figure 3B, Figure S6A). Taken together, the wild-type strain was able to outcompete the *sdaA* mutant in 16 of the 18 intraperitoneally infected animals. These results indicate that in contrast to the *ggt* and *ansB* mutants, which exhibited a tissue-specific colonization defect, the *C. jejuni sdaA* mutant strain was defective for persistence in all tissues tested. It also stresses the importance of serine catabolism for *C. jejuni* 81-176 *in vivo* since the growth of the *sdaA* mutant was less dramatically affected when co-cultivated with the wild-type strain *in vitro* (Figure S7).

### The Role of Peb1A on the Persistence of *C. jejuni* in Different Murine Tissues

**Figure 4:**
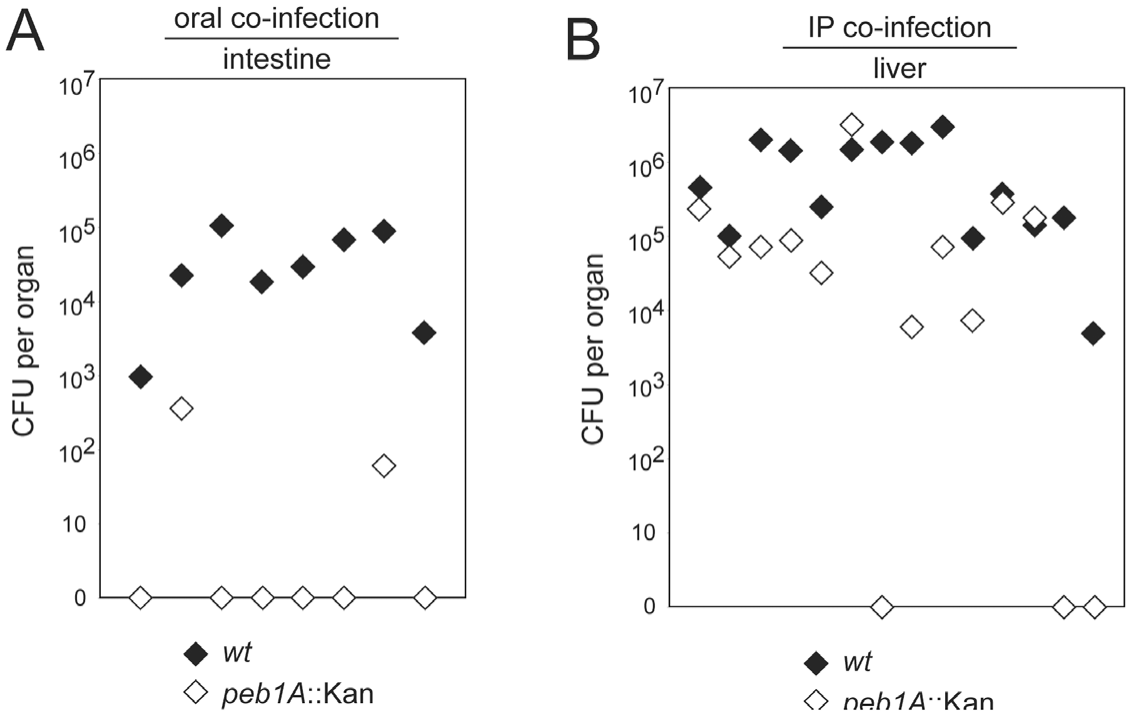
Peb1A-mediated amino acid uptake in *C. jejuni* 81-176 is important for its persistence in the murine intestine and liver. Mice were orally (A) or intraperitoneally (B) co-infected with the *C. jejuni* 81-176 wild-type strain (black diamonds) and the *peb1A* mutant (white diamonds). The bacterial load in the intestine and liver of the infected animals was examined 5 weeks after infection. The pairs of black and white diamonds indicate the CFU numbers of *C. jejuni* 81-176 and its *peb1A* mutant that could be recovered from the indicated organs of one animal. The combined results of two independent infection experiments are shown. The Mann–Whitney U test was used to calculate the P values for the statistical differences between the CFU numbers of the different strains: P<0.001 (A) and P<0.05 (B).

The pronounced and broad colonization defect of the *sdaA* mutant suggests a particularly important role for serine catabolism in the ability of *C. jejuni* to persist both in the intestine and systemically. This is in contrast to the more moderate and tissue specific defects observed in *C. jejuni* mutants unable to utilize other amino acids such as glutamine or asparagine [43], [51]. To gain more insight into the relative contribution of amino acids in *C. jejuni* colonization, we examined the ability of a *C. jejuni peb1A* mutant strain to colonize different tissues. Peb1A is a periplasmic aspartate-glutamate binding protein [40], [65] that is required for the growth with aspartate and glutamate by *C. jejuni* [40], [43]. Certain *C. jejuni* isolates like *C. jejuni* 81-176 secrete an asparaginase and γ-glutamyltranspeptidase enabling the efficient deamination of asparagine and glutamine and subsequently the additional generation of aspartate and glutamate in the periplasm [43]. Thus a *C. jejuni* 81-176 *peb1A* mutant is simultaneously defective in its ability to utilize all four amino acids resulting in slightly reduced growth efficiency when grown in rich media *in vitro* as compared to the wild-type strain during co-cultivation experiments (Figure S7). Equal numbers of wild-type *C. jejuni* 81-176 and the isogenic *peb1A* mutant were administrated orally or intraperitoneally into *myd88*−/− C57BL/6 mice. A significant difference in the bacterial load of the intestine with wild-type strain and its *peb1A* mutant was observed 5 weeks after oral infection and in all cases the wild-type strain was able to outcompete the mutant (Figure 4A, Figure S6B). Moreover, the *peb1A* mutant could not be recovered in 6 of the 8 infected animals. This observation is in good agreement with previous results, describing the colonization defect of the *C. jejuni* 81-176 *peb1A* mutant in BALB/c mice [66]. Upon intraperitoneal co-infection the wild-type strain was recovered from the liver in higher numbers than the mutant strain in 9 out of 14 infected mice, and the *peb1A* mutant of *C. jejuni* 81-176 was not recovered from the liver of three infected animals at all (Figure 4B, Figure S6B). These experiments clearly demonstrated the importance of Peb1A for the persistence of *C. jejuni* 81-176 in the liver.

### Proline Metabolism in *C. jejuni* 81-176

**Figure 5:**
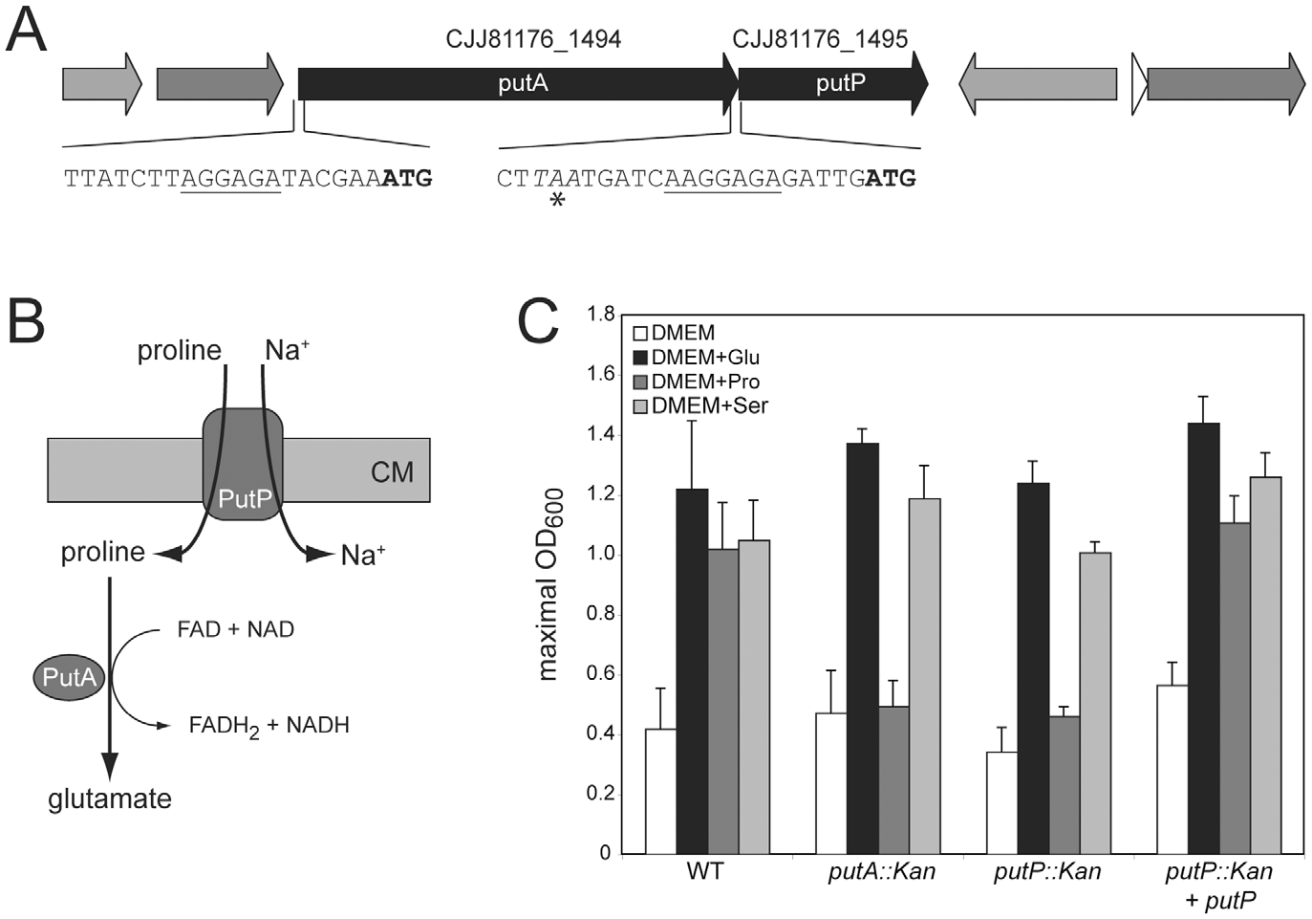
Proline metabolism in *C. jejuni* 81-176. (A) The gene locus of *C. jejuni* 81-176 encoding the proline permease PutP and the bifunctional proline dehydrogenase PutA is shown. The start codons (bold) and Shine-Dalgarno-sequences (underlined) of *putA* and *putP* as well as the intergenic region are indicated below the gene region. (B) Schematic model of the proline uptake system in *C. jejuni*. After proline is being co-imported with sodium ions by the permease PutP into *C. jejuni*, the cytoplasmic, bifunctional proline dehydrogenase PutA converts proline into glutamate. (C) The ability of *C. jejuni* 81-176 wild-type strain, its isogenic *putA* and *putP* mutants as well as the complemented *putP* mutant strains to utilize proline as a growth substrate. Values are the mean ± SD of at least three determinations of maximal reached optical density by *C. jejuni* 81-176 and its mutant derivates in liquid culture. The growth of *C. jejuni* in DMEM supplemented with 20 mM glutamate, proline or serine occurred over 24 hours at 37°C in 10% CO_2_.

Several studies have documented that *C. jejuni* can utilize proline for *in vitro* growth [38], [39], [42], [43]. However, the specific genes involved in the catabolism of proline have not been experimentally investigated. All available *C. jejuni* genome sequences [33], [34], [51], [56]–[62], [64] show the presence of a homologue of PutP, a proline-sodium symporter, and a homologue of PutA that catalyzes the conversion of proline to glutamate (Figure 5A and 5B). PutA represents a bifunctional enzyme combining the enzymatic activities of a proline dehydrogenase and a 1-pyrroline-5-dehydrogenase. Because reducing equivalents in the form of FADH and NADH are generated by the PutA-mediated oxidation of proline to glutamate, the catabolism of proline not only provides energy but also affects the intracellular redox level of *C. jejuni* [67]. The arrangement of *putA* (CJJ81176_1495) and *putP* (CJJ81176_1494) in the genome of *C. jejuni* 81-176 suggests that the two genes are organized in an operon structure (Figure 5A), which is in contrast to the organization of the *putA* and *putP* orthologues in enterobacteria (Figure S8). PutA is well conserved in the epsilon-bacteria and exhibits high homology to PutA orthologues in the unrelated species *Bacteroides spp*. and *Corynebacterium spp.* (Table S3). Likewise, PutP is highly conserved in *C. jejuni* and exhibits significant homology to the PutP orthologues found in *Bacillus spp.*, *Enterobacter* spp. and *Pseudomonas* spp. (Table S4). We therefore investigated the potential role of the *putA* and *putP* homologues in the ability of *C. jejuni* 81-176 to utilize proline. We found that insertional inactivation of *putA* or *putP* completely abolished the ability of *C. jejuni* to utilize proline as a carbon source, whereas no defects in the utilization of glutamate or serine as growth substrates were detected (Figure 5C). These results indicated that PutA and PutP are required for the catabolism of proline in *C. jejuni*. Interestingly, an abolished *putP*-mediated proline catabolism does not affect the mutant in growth competition experiments with the wild-type strain when co-cultivated *in vitro* using nutrient rich BHI medium (Figure S7).

### Role of Proline Catabolism in *C. jejuni* Mouse Colonization

**Figure 6:**
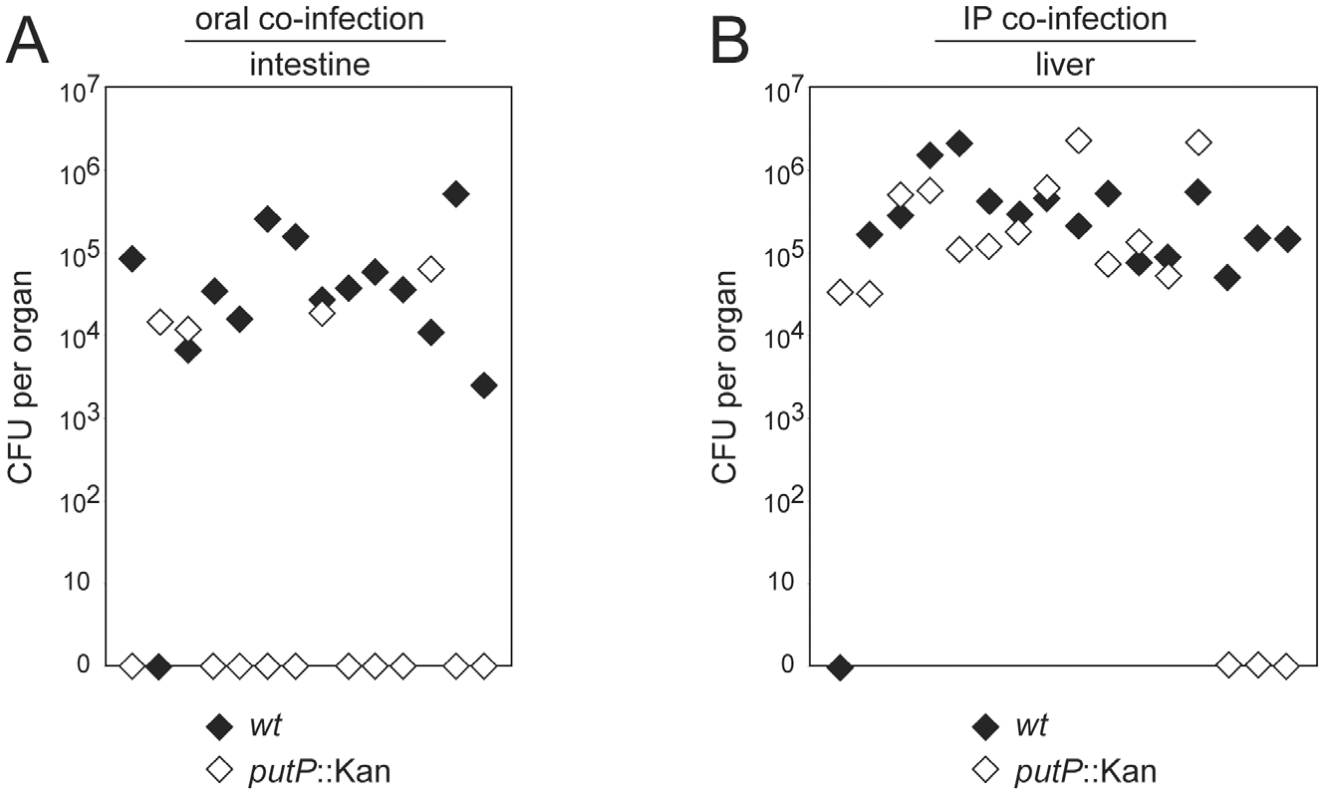
Tissue-specific impact of proline catabolism on *C. jejuni* 81-176 mouse colonization. Shown is the colonization efficiency of *C. jejuni* 81-176 wild-type (black diamonds) and its isogenic *putP* mutant strain (white diamonds) in a murine model of infection. The colonization of the intestine (A) and liver (B) was evaluated 5 weeks after oral (A) or intraperitoneal (B) co-infection of *myd88* ^−/−^, *nramp* ^−/−^ mice, respectively. Each pair of black and white diamonds represents the CFU numbers of *C. jejuni* 81-176 wild-type strain and its *putP* mutant recovered from the indicated organs of an individual animal. The combined results of four (A) and three (B) independent infection experiments are shown. The Mann–Whitney U test was used to calculate the P values for the statistical differences between the CFU numbers of the different strains: P<0.001 (A) and P ≥ 0.05 (B).

Since only limited information is available on the contribution of proline catabolism to bacterial pathogen colonization, we examined the role of proline utilization in *C. jejuni* 81-176 mouse colonization. We infected *myd88*−/− mice orally or intraperitoneally with equal numbers of wild-type *C. jejuni* 81-176 and the isogenic *putP* mutant strain and examined bacterial loads in different tissues. We found significantly lower numbers of the *C. jejuni putP* mutant in the intestine of infected mice 5 weeks after the oral co-administration of the two bacterial strains (Figure 6A, Figure S6C). These results indicate that proline metabolism is important for efficient *C. jejuni* colonization of and persistence in the mouse intestine of infected animals. In contrast, we found no difference between the bacterial loads of wild-type strain and the *putP* mutant in the livers of infected animals 5 weeks after intraperitoneal administration (Figure 6B, Figure S6C). These results indicate that proline catabolism, like glutamine catabolism, is dispensable for the colonization of systemic tissues by *C. jejuni.* Therefore, the utilization of proline also confers a tissue specific advantage for *C. jejuni* colonization.

### Capability of *C. jejuni* Mutants Defective in Amino Acid Catabolism to Cope with Oxidative and Osmotic Stress

**Figure 7:**
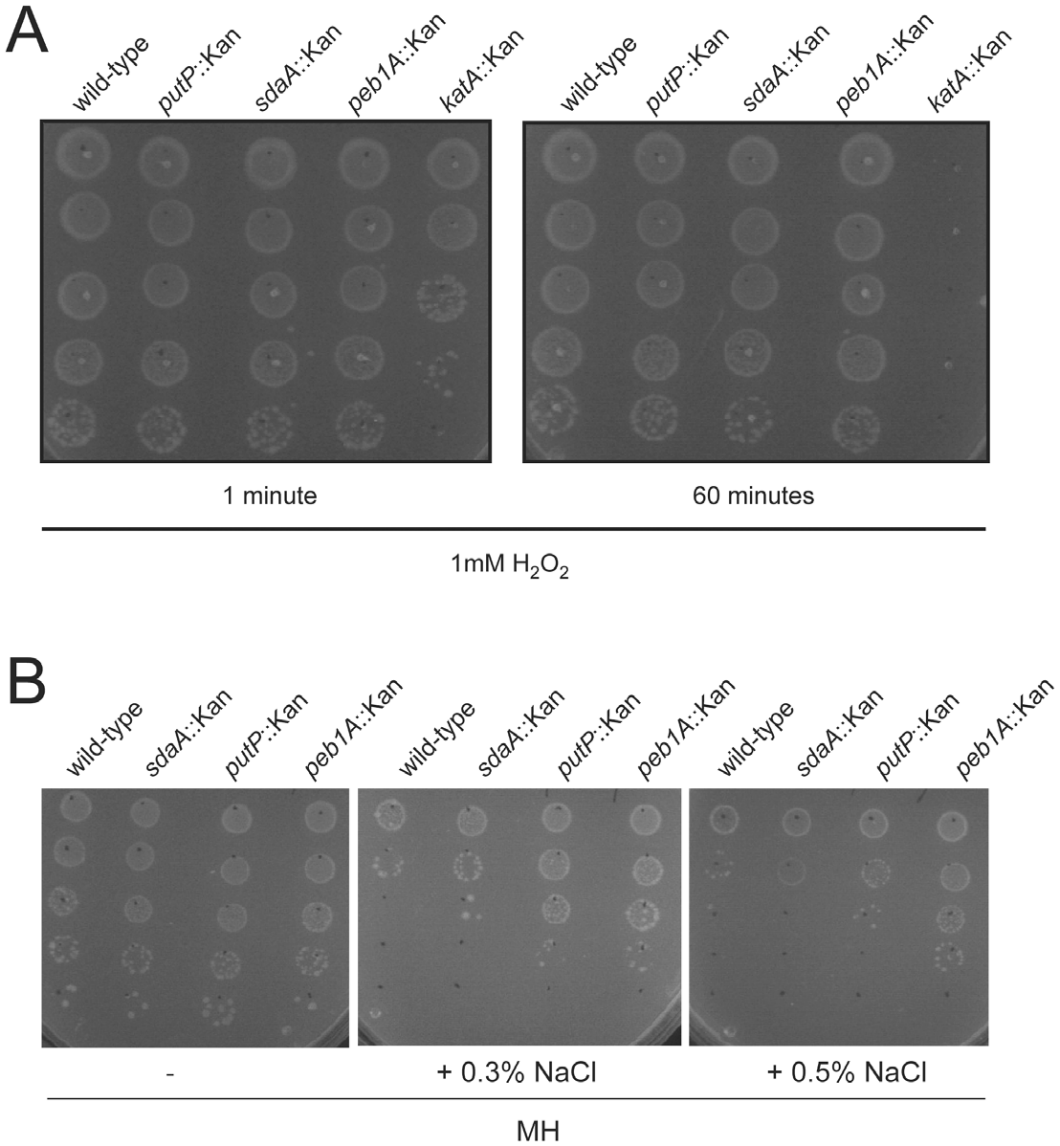
Effect of oxidative and osmotic stress on the viability of *C. jejuni* 81-176 mutants with defective amino acid catabolism. (A) Shown is the capability of *C. jejuni* 81-176 wild-type and the s*daA*, *putP* and *peb1A* mutants to survive oxidative stress caused by treatment with H_2_O_2_. Serial dilutions of the bacterial suspensions after the different treatments were spotted on Brucella broth agar plates after the indicated incubation times. (B) The resistance of *C. jejuni* 81-176 wild-type and its isogenic *sdaA*, *putP* and *peb1A* mutants to variable osmotic stress conditions is presented. Liquid cultures were adjusted to an OD_600_ of 0.1 and serial dilutions were spotted on Mueller Hinton agar plates supplemented with 0%, and 0.5% NaCl.

It has been recently shown that mutations in the amino acid transporters *paqP* and *paqQ* have altered tolerance to osmotic and oxidative stress [68]. Therefore we tested the ability of the *C. jejuni* 81-176 wild-type as well as its *peb1A*, *putP, sdaA* and *katA* mutants to cope with oxidative stress by exposing them to hydrogen peroxide (H_2_O_2_). Whereas the viability of the *katA* mutant was dramatically affected by the H_2_O_2_ treatment similar to previous descriptions [69], we found no difference between the *peb1A*, *putP* and *sdaA* mutants and wild-type *C. jejuni* 81-176 in their plating efficiency after exposure to oxidative stress (Figure 7A). To examine whether mutations in *peb1A*, *putP* and *sdaA* affect the ability of *C. jejuni* to cope with osmotic stress, we examined the plating efficiency of the different mutants in media containing varying concentration of sodium chloride. None of the tested mutants showed an increased sensitivity to osmotic stress (Figure 7B). The *putP* and *peb1A* mutants were even more resistant to 0.5% NaCl than the wild-type strain. We do not yet know the cause of this unexpected phenotype.

Taking together these results indicate that the colonization defect observed in these mutant strains is due to their defective acquisition of growth substrates rather than their elevated sensitivity to oxidative and osmotic stresses.

## Discussion

Although *C. jejuni* is a fastidious bacterium *in vitro* and exhibits significant metabolic restrictions in comparison to other enteropathogenic bacteria [35], [70], this pathogen requires a remarkably low infection dose to successfully colonize different hosts [71], [72]. Therefore *C. jejuni* must have the capability to acquire and utilize nutrients efficiently to circumvent the competition by the host microbiota. Unlike other enteropathogenic bacteria *C. jejuni* is unable to use glucose as growth substrate [35] and only a subset of *C. jejuni* isolates harbor a *fucP* gene cluster that facilitates the catabolism of fucose [36], [37]. Instead of carbohydrates, the glucogenic amino acids aspartate, asparagine, glutamate, glutamine, proline and serine represent important nutrients fueling the *in vitro* growth of *C. jejun*i [38]–[40], [42], [43], [45], [55], [68].

So far, only a few studies have examined the impact of amino acid catabolism on the capacity of *C. jejuni* to thrive in different hosts [38], [55], [68] and to persist in different tissues [43], [51]. Velayudhan and Kelly (2004) have previously shown that a *sdaA* mutant of *C. jejuni* NCTC 11168 is defective in utilizing serine and exhibits a significant colonization defect in chickens. We were interested to examine whether serine catabolism provides *C. jejuni* with an advantage to colonize animals other than chickens. We found that a *C. jejuni sdaA* mutant is required for colonization in a mouse model of infection indicating that serine utilization may be required for the colonization of many animal species. Other metabolic traits exhibit similar benefits for the infection process of *C. jejuni* in various hosts. For example, C. *jejuni* mutants incapable to use glutamine and glutathione as growth substrates, not only showed a colonization defect in mice [51] but also in chicken [73]. Furthermore, the AspA-mediated aspartate utilization is important for the colonization of *C. jejuni* in murine [74] and avian [38] hosts. Besides amino acid catabolism, the FeoB-mediated uptake of ferrous iron provides a general benefit for *C. jejuni* during the colonization of the avian and porcine gut [49]. Interestingly, not all physiological properties of *C. jejuni* exhibit a host-independent affect on its colonization proficiency but show a host-specific defect instead: the ability of some *C. jejuni* strains to metabolize fucose is required for efficient colonization of pigs but not chickens [37] and a *C. jejuni* mutant defective in gluconate respiration shows a diminished colonization efficiency in chickens but not in mice [52]. Future studies will be required to clarify how specific metabolic traits of *C. jejuni* shape its persistence in specific hosts.

Several *in vitro* studies have shown that *C. jejuni* utilizes proline as a growth substrate [38], [39], [42]. It was suggested that proline represents a less favoured growth substrate in comparison to glutamate or serine for *Campylobacter* [42]. Nevertheless, proline catabolism seems to be a highly conserved phenotypic trait in *C. jejuni* [43] and *C. coli* isolates (Wensel and Hofreuter, unpublished data). We have shown here that proline catabolism is required for efficient colonization of the murine intestine. Strikingly, the inability of the *putP* mutant to utilize proline for growth did not affect its persistence in the murine liver. This tissue-specific colonization phenotype resembles the behaviour of the previously characterized *ggt* mutant of *C. jejuni* 81-176, which was also defective for the colonization of the murine gut but could colonize the liver of the infected mice to the same extent as the wild-type strain [43], [51]. The importance of proline catabolism in host colonization has also been shown in *Helicobacter* spp. [75]–[77], *Rhizobium meliloti* [78] and *Staphylococcus aureus* [79]. The PutA mediated oxidation of proline to glutamate generates the reducing equivalents FADH and NADH. Subsequently, the electrons are transferred to the respiration chain and partially to molecular oxygen generating H_2_O_2_ [76]. Future experiments have to clarify to what extent the observed colonization defect of the *C. jejuni* 81-176 *putP* mutant is the result of a reduced energy catabolism or an imbalanced intracellular redox environment.

The protective role of amino acid metabolism in counteracting osmotic and oxidative stress is well documented in enteric bacteria [80]. In particular, the amino acids glutamate and proline serve as osmoprotective substances in various bacteria [81], [82]. Though the effect of glutamine metabolism on the ability of *C. jejuni* to withstand osmotic and oxidative stress was recently described [68], the *peb1A*, *putP* nor *sdaA* mutants of *C. jejuni* 81-176 did not show reduced sensitivity to these types of stresses. These observations indicate that the reduced colonization efficiency of the here-described *C. jejuni* 81-176 mutants is most likely due to their inability to utilize the specific amino acids and not to other secondary effects.

The present study has extended our previous observations indicating that the catabolism of certain amino acids confers tissue-specific advantages for *C. jejuni* colonization: Serine supports the growth of *C. jejuni* 81-176 in the murine intestine and liver, indicating that the uptake of neither lactate nor of pyruvate could compensate the restricted catabolism of the *sdaA* mutant at either infection site. The catabolism of proline and glutamine provides an advantage for the persistence of *C. jejuni* 81-176 in the intestine but not in the liver of infected mice. As glutamate is generated by the catabolism of glutamine as well as proline, our studies suggest that glutamate is a crucial growth substrate for the colonization of the murine intestine by *C. jejuni* 81-176. These results also imply that glutamate is not available in sufficient amounts at the intestinal niche that *C. jejuni* occupies, since glutamate would be expected to overcome the catabolic defects of the *C. jejuni* 81-176 *ggt* and *putA* mutants as shown *in vitro*. We have demonstrated previously that asparagine utilization by *C. jejuni* 81-176 is especially beneficial for the persistence in the liver but not the intestine [43]. The importance of asparagine catabolism for the liver colonization by *C. jejuni* 81-176 is further supported by our here-presented observations that the *peb1A* mutant, defective in the utilization of asparagine, aspartate, glutamate and glutamine, exhibits a similar colonization defect in the murine liver as an *ansB* mutant of *C. jejuni* 81-176, solely incapable to utilize asparagine [43]. Interestingly, the tissue specific necessity for certain amino acids has also been shown in *S. aureus* [83] and recently in *Francisella novicida*, [84].

There is remarkable variability in the metabolic profiles of *C. jejuni* isolates [36], [43], [44], [85] and serine catabolism is one such non-conserved metabolic trait in *C. jejuni*. Though *C. jejuni* ATCC 33251 and RM1221 are not able to utilize serine under the *in vitro* growth condition tested, we did not detect any obvious changes in the coding sequences of either *sdaA* or *sdaC* that could account for this phenotype. Furthermore, both strains express the *sdaCA* operon and are able to take up serine from the culture medium like *C. jejuni* 81-176. As *C. jejuni* ATCC 33251 and RM1221 showed only slightly reduced SdaA activities in comparison to the strain *C. jejuni* 81-176, future experiments have to clarify if these differences are responsible for the abolished serine catabolism. More likely, defects in other, yet unidentified genes could be required for serine utilization and may explain the inability of certain *C. jejuni* strains to utilize this amino acid.

Though it has been demonstrated for *C. jejuni* NCTC 11168 that serine catabolism is important for the colonization of chickens [45], *C. jejuni* RM1221 with its inability to utilize serine was originally isolated from chicken [86]. Considering the metabolic diversity of *C. jejuni* isolates, further studies will be required to elucidate any metabolic characteristics *C. jejuni* RM1221 may harbor that could compensate its inability to grow with serine. In this context, *C. jejuni* RM1221 showed the best growth with proline in comparison to all other wild-type strains tested (Figure 1B). Though *C. jejuni* is non-glycolytic [35], certain isolates like *C. jejuni* NCTC 11168 and RM1221 encode for genes involved in fucose catabolism [37]. Differences in the utilization of fucose or certain other yet not identified carbohydrates perhaps allow *C. jejuni* RM1221 to overcome its restricted amino acid catabolism during the colonization process. Also future studies have to examine if the efficient catabolism of citric acid cycle intermediates like fumarate or succinate [38], [44] could compensate the abolished serine catabolism in RM1221 *in vivo*.

We are just beginning to understand the specific nutrients upon which pathogenic bacteria rely during host colonization [1], [2]. It is becoming increasingly clear that there are significant differences between the metabolic requirements *in vitro* and *in vivo*. Various studies have shown a clear correlation between certain metabolic traits and the ability of *C. jejuni* to colonize different animals. So understanding the metabolism of *C. jejuni* during the infection process may facilitate the development of anti-microbial drugs that specifically target essential metabolic pathways in *C. jejuni*.

## Materials and Methods

### Bacterial Strains, Media and Culture Conditions

*C. jejuni* was routinely cultivated on Brucella broth agar plates and tryptic soy agar (TSA) or Columbia agar plates containing 5% (vol/vol) sheep blood at 37°C in 10% CO_2_ atmosphere. To select for *C. jejuni* mutants carrying antibiotic-resistant determinants, either kanamycin, chloramphenicol or erythromycin were added to Brucella broth (BB) agar plates with a final concentration of 50 mg/l, 7.5 mg/l or 10 mg/l, respectively. For growth experiments in 4 ml liquid cultures, *C. jejuni* was inoculated with a starting OD_600_ of approximately 0.1 in Brain Heart Infusion (BHI; BD 237500) medium or in defined Dulbecco’s Modified Eagle’s Medium (DMEM; Invitrogen 11965), supplemented with Fe(II)-ascrobate (Sigma; AO207) containing 20 mM of the indicated amino acids (Sigma). The liquid cultures were incubated in a rotating wheel (50 rpm) at 10% CO_2_ and 37°C. Bacterial growth in liquid cultures was monitored with a spectrophotometer (Spectronic 20, Genesys) by measuring the optical density of the cultures at 600 nm (OD_600_). All *C. jejuni* strains were stored at –80°C in BHI broth containing 40% glycerol.

*Escherichia coli* DH5α was cultured at 37°C on Luria-Bertani (LB) medium agar plates supplemented when necessary with the antibiotics kanamycin (50 mg/l), chloramphenicol (30 mg/l) or erythromycin (100 mg/l).

### Natural Transformation of *C. jejuni*

Natural transformation was used to genetically manipulate *C. jejuni* 81-176 similar as described previously (18). Briefly, *C. jejuni* 81-176 was resuspended in 1 ml BHI medium with an OD_600_ of 0.1. Approximately 1 µg of DNA was added and the bacterial suspension was incubated overnight (37°C, 10% CO_2_). The next day bacteria were spread onto Brucella Broth plates supplemented with the appropriate antibiotics.

### Construction of Recombinant Plasmids for Generating *C. jejuni* Mutants

To generate an isogenic *sdaA* (CJJ81176_1615) mutant of *C. jejuni* 81-176 the 1.3 kb *sdaA* gene with 500 bp of the upstream and downstream regions was PCR amplified with the primers sdaA_fwd and sdaA_rev (see Table S6 for primer information) and cloned into the *Bam*HI/*Pst*I-restriction sites of pBluescriptIIKS. The *aphA*3-cassette was released from pILL600 (see Table S7 for plasmids used in this study) by *Sma*I digestion and introduced into a unique *Nhe*I site within the cloned *sdaA* gene by blunt end ligation. The resulting plasmid pSB3622 was introduced into *C. jejuni* 81-176 by natural transformation as described above and *C. jejuni* transformants were selected with Brucella broth plates containing kanamycin (50 mg/l). To complement the *C. jejuni* 81-176 *sdaA* mutant strain, the *sdaA* gene of *C. jejuni* 81-176 was PCR amplified with its Shine-Dalgarno sequence by using the primers OW2 and OW3 and cloned into the *NcoI*/*Spe*I sites of pSB3021 [30]. The resulting plasmid pOW4 was verified by sequencing and transformed into the *sdaA* mutant of *C. jejuni* 81-176. Transformants were selected on Brucella broth agar plates supplemented with chloramphenicol (7.5 mg/l). A *sdaC* mutant of *C. jejuni* 81-176 was generated by introducing an erythromycin resistance marker into the gene CJJ81176_1616: a 2.9 kb *sdaA*-*sdaC* fragment was PCR amplified with the primers DHO285 and DHO284 and cloned into the *Xba*I/*Xho*I-restriction sites of pBluescriptIISK resulting into the plasmid pSK1. An erythromycin cassette was amplified by PCR with the oligonucleotides DHO349 and DHO350 using pDHO36 as template. The PCR fragment was inserted into a unique *Eco*RI site of *sdaC* generating pOW34. This plasmid was transformed into *C. jejuni* 81-176. Transformants with an interrupted *sdaC* gene grew on Brucella Broth plates supplemented with erythromycin (10 mg/l).

To construct the *putA* (CJJ81176_1495) insertion mutant of *C. jejuni* 81-176 a gene fragment of about 3.0 kb was PCR amplified from the chromosome of *C. jejuni* 81-176 using the oligonucletides DHO231_putA-5′ and DHO232_putA-3′. The PCR product was cloned as *Xba*I/*Xho*I-fragment into the compatible digested vector pBluescriptIIKS generating pSB3030. The plasmid was electroporated into the dam^-^ *E. coli* strain GM2199 (Table S7) and a singular *Cla*I restriction site of the *putA* gence was used to insert an *aphA*3 kanamycin resistence cassette, which was isolated as *Cla*I-fragment from pILL600. The resulting plasmid pSB3031 was introduced into *C. jejuni* 81-176 by natural transformation. Transformants with an inactivated *putA* gene were selected on Brucella broth plates supplemented with kanamycin (50 mg/l). A *putP* mutant of *C. jejuni* 81-176 was constructed by PCR amplification of the *putP* gene (CJJ81176_1494) with the primer DHO233_putP-5′ and DHO234_putP-3′ and cloning of the *Xba*I/*Xho*I-digested PCR fragment into pBluescriptIIKS. A unique *Hind*III restriction site within the *putP* gene was used to insert the *aphA3* resistance marker that was PCR amplified from pILL600 with the primers DHO2 and DHO52 resulting in plasmid pSB3032. The *putP* mutant was obtained by transformation of pSB3032 into *C. jejuni* 81-176 and selection of transformants on Brucella broth agar plates supplemented with kanamycin (50 mg/l). Complementation of the *putP* mutant was done by PCR amplification of the *putP* gene of *C. jejuni* 81-176 with the primes DHO286_putP-SD and DHO287_putP-3′, followed by cloning the *Nco*I−/*Spe*I-digested PCR fragment into the complementation vector pSB3021 resulting in the plasmid pSB3033. Natural transformation of *C. jejuni* 81-176 with plasmid pSB3033 generated the complemented *putP* mutant. A *katA* insertion mutant was constructed by *in vitro* transposon mutagenesis as described before [87] using a modified Tn552 transposon with an outward oriented *cat* and *aphA3* cassette. The generation of the isogenic *peb1A* mutant has been described before [74].

### Gene Expression Analysis by Reverse Transcriptase PCR Analysis

For the extraction of total RNA from *C. jejuni* aliquots of bacterial liquid cultures were taken after 18 hours of incubation and added to two volumes of RNAprotect bacteria reagent (Qiagen). The mixture was mixed immediately and incubated for 10 minutes at room temperature. After centrifugation (10 min, 7000 rpm) the supernatant was removed and the pellet was used for RNA extraction by RNeasy Mini kit (Qiagen) following the manufacturer’s instructions. RNA was eluted in 40 µL nuclease free water and remaining DNA was digested by DNase I (NEB) following the manufacturer’s instructions. The quantity and purity of RNA were measured spectrophotometrically using a NanoDrop 1000 (Thermo Scientific). For cDNA synthesis 40 ng RNA were added to a total volume of 12 µL nuclease free water containing 1 µL dNTPs and 1 µL random hexameres (Qiagen). The SuperScript II Kit (Fermentas) was used for the reverse transcription according to the manufacturer’s instructions. Equal amounts of the synthesized cDNA was used as templates for the partial PCR amplification of the *sdaA* gene with the primers DHO352 and OW10 generating a 628 bp PCR product. As reference the *recA* gene was amplified with the primers SR15 and SR16. The PCR was carried out in a total volume of 50 µL containing 2 µL cDNA, 5 µL TaqBuffer, 2 µL dNTPs, 1.5 µL MgSO4, 0.75 µL Primers each, 0.5 µL Taq polymerase (NEB), and 38.5 µL water with 30 cycles of 95°C for 15 s (denature), 58°C for 60 s (annealing), and 72°C for 45 s (extension). As a control to exclude amplification of DNA contamination an aliquot of RNA without RT treatment was used instead of cDNA as PCR template.

### Measurement of Serine Dehydratase Activity in Cell Extracts

*C. jejuni* was grown in liquid cultures (200 ml BHI) for 18 hours at 37°C under microaerophilic conditions using an Anaerocult C pack (Merck). Cells were harvested by centrifugation (20 min, 7000 rpm, 4°C), washed with cold 0,1 M triethanolamine buffer (pH 7,5), and resuspended in 500 µl of the same buffer. Lysis was followed by sonification with the Branson Sonifier 450 (5 min, 30% of duty). The cell extracts were clarified from cell debris by centrifugation (30 min, 14000 rpm, 4°C) and the supernatants were used for the enzymatic assay the same day. The amount of total protein was determined using the Pierce® BCA Protein Assay Kit (ThermoScientific). The serine dehydratase activity of the cell lysates was measured as described previously [88]: Briefly, the assay was carried out in a total volume of 1 mL 50 mM HEBS buffer (pH 8,4) containing 150 µM NADH (Roth), 2,7 U LDH (Sigma), and cell-free extract. The reaction was started by adding 50 mM L-serine (Sigma Aldrich) and the decrease of NADH was monitored photometrically at 360 nm.

### Serine Uptake by *C. jejuni*

For the comparison of serine uptake from the growth medium by different *C. jejuni* isolates, cultures of *C. jejuni* 81-176, *C. jejuni* RM1221 and *C. jejuni* 33251 were grown in 50 ml Hank`s Balanced Salt Solution supplemented with Fe^2+^ in the form of Iron(II)-ascorbate (Sigma, A0207), MEM Vitamin Solution (Invitrogen, 11120) and 1% Casamino acids (Roth, AE41.1) as a carbon source. All cultivations were done at 37°C and 150 r.p.m. on a rotary shaker under oxygen reduced atmosphere using Anaerocult C-Packs (Merck, 116275). The uptake of serine in *C. jejuni* was studied by analyzing the changes in the amount of serine in the culture supernatants. Samples of 2 ml of supernatant were taken at the particular time points. The bacterial cells were removed by centrifugation (17000 *g*, 5 min, 4°C) and an aliquot of the filtrated supernatant was used for GC-MS analysis. This aliquot (100 µl) of the supernatant was diluted in 500 µl water, containing 4 µg of ribitol as an internal standard. The samples were mixed, dried under vacuum at room temperature and stored at −20°C. Derivatization of the samples was done by using 40 µl pyridine, containing methoxyamine hydrochloride (20 mg ml^-1^) and 60 µl N-Methyl-N-trimethylsilyltrifluoro-acetamide (MSTFA). The GC-MS analysis was done on a Thermo GC Ultra coupled to a DSQII mass spectrometer equipped with an AS3000 autosampler (ThermoScientific, Dreieich, Germany) as previously described [89] with the following exceptions: Helium flow was set to 1.1 ml min^-1^ and the temperature was increased to final 325°C. Solvent delay time was 5.80 min. Data analysis was performed with Metabolite Detector (version 2.07**;** [90]) as described [89] and quantification was done by using one unique fragment ion for each metabolite. For statistical analysis, data were first normalized dividing the peak area of every detected compound in each sample by the peak area of the respective internal standard ribitol. Before further data evaluations derivates belonging to one substance were summarized. Afterwards the mean and the standard deviation of 5 biological samples with 2 technical replicates each were calculated.

### Osmotic Stress Assay

The capability of *C. jejuni* to tolerate osmotic stress was assayed by its growth on Mueller Hinton broth plates supplemented with various concentration of NaCl. *C. jejuni* cells were harvested from blood pates, resuspended in BHI medium and cultured for several hours in an atmosphere of 10% CO_2_ at 37°C. The bacterial suspension was adjusted to an OD_600_ of 0.1 and serially diluted. From each dilution 10 µl were spotted on Mueller Hinton **(**Difco #225220) agar plates supplemented with 0% and 0.5% NaCl. The plates were incubated in 10% CO_2_ at 37°C.

### Oxidative Stress Assay

The capability of *C. jejuni* to cope with oxidative stress was evaluated by exposure to 1 mM hydrogen peroxide (H_2_O_2_; Applichem A4269.1000) in Brucella broth. A bacterial suspension of an OD_600_ of 0.1 was incubated for various times in the H_2_O_2_-containing media. Afterwards bacteria were serially diluted and 10 µl of each dilution were spotted on Brucella Broth agar plates. The plates were incubated in 10% CO_2_ at 37°C.

### Co-cultivation Experiments

To compare the growth characteristics of *C. jejuni* 81-176 and its isogenic mutants *in vitro*, 10 ml of BHI medium were inoculated in a flask with an equal amount of wild-type and mutant strain to a final OD_600_ of 0.1. The flask was incubated in a jar at 37°C and 150 r.p.m. on a rotary shaker under oxygen-reduced atmosphere using Anaerocult C-Packs (Merck, 116275). After 20 hours, aliquots of the culture were taken and plated after serial dilution on Brucella broth agar plates and Brucella broth agar plates containing kanamycin to enumerate the wild-type and mutant strain. To determine if *C. jejuni* 81-176 or its mutants have growth advantage under *in vitro* cultivation condition, the competitive index was calculated: CI = (mutant_output_/wild-type_output_)/(mutant_inoculum_/wild-type_inoculum_). Input represents the CFUs of the inoculum and output the recovered CFUs after 20 hours.

### Mice Infection Experiments

For animal infection experiments *myd88* ^−/−^, *nramp1* ^−/−^, male age-matched (6 to 8 weeks old) C57BL/6 mice were used as previously described (72). Briefly, *C. jejuni* 81-176 wild-type strain and its isogenic mutants were grown in BHI medium to an OD_600_ of about 0.6 and then applied in equal amounts of 10^9^ or 10^7^ CFUs for oral or intraperitoneal (i.p.) co-infection of mice, respectively. The colonization levels of the different *C. jejuni* strains were monitored by enumerating the number of CFUs in the feces of inoculated animals. At the end of the experiment mice were sacrificed, and the intestine and liver were aseptically removed and homogenized in HBSS. The bacterial loads in their organs of infected animals were enumerated by plating on Brucella broth agar plates containing *Campylobacter*-selective supplements (Oxoid SR0167E) as well as on *Campylobacte*r-selective plates containing the antibiotic kanamycin (50 mg/l) to differentiate between *C. jejuni* wild-type and mutant strains. Statistical analysis of the co-infection experiments were carried out with the non-parametric, two-sided Mann–Whitney U test ( = *Wilcoxon rank-sum test)* using a Web-based algorithm (http://elegans.som.vcu.edu/~leon/stats/utest.html). The Mann–Whitney U test was used to examine if the observed differences in the numbers of recovered CFUs of the wild-type strain and its isogenic mutants from co-infected mice were significant. Therefore the recovered CFUs of the wild-type strain and the CFUs of the mutant strains were grouped in two datasets and directly analyzed with the web-based algorithm. The competitive index was determined for each infected mice with the equation (mutant_output_/wild-type_output_)/(mutant_inoculum_/wild-type_inoculum_). Output numbers equal the bacterial loads of wild-type and mutant strains recovered from the organs. The CFUs of the inoculums were determined through plating of serial dilutions of the bacterial suspensions that had been adjusted to an equal number of wild-type and mutant bacteria. All animal work was approved by the Yale University Institutional Animal Care and Use Committee (IACUC) and all animals were maintained as well as animal experiments were conducted according to the guidelines of the IACUC regulations.

## Supporting Information

Figure S1 **Nucleotide sequence comparison of the L-serine dehydratase** ***sdaA*** **genes in indifferent** ***C. jejuni*** **isolates.** The nucleotide sequences of sdaA open reading frames from different *C. jejuni* strains were compared using ClustalW (www.ebi.ac.uk/Tools/msa/clustalW2). The sources for the DNA sequences are as follows: CG8486 (Cj8486_1666c; NZ_AASY01000001.2), ATCC 33251 (this study), CF93-6 (CJJCF936_1718; NZ_AANJ01000002.1), 84-25 (CJJ8425_1708; NZ_AANT02000001.1), NCTC 11168 (Cj1624c; NC_002163.1), IA3902 (CJSA_1536; CP001876.1), DFVF1099 (CSQ_0902; ADHK01000020.1; this study), 305 (CSS_1725; ADHL01000259.1; this study), RM1221 (CJE1796; NC_003912.7), S3 (CJS3_1705; CP001960.1), 260.94 (CJJ26094_1675; NZ_AANK01000006.1), ICDCCJ07001 (ICDCCJ07001_1539; NC_014802.1), HB93-13 (CJJHB9313_1615; NZ_AANQ01000001.1), 81-176 (CJJ81176_1615; AASL01000001.1), 81116 (C8J_1526; NC_009839.1), M1 (CJM1_1565; CP001900.1), 327 (CSU_0676; ADHM01000033.1; this study), 1336 (C1336_000330073; NZ_ADGL01000024.1), CG8421 (Cj8421_1678; NZ_ABGQ01000002.1), 414 (C414_000010127; NZ_ADGM01000001.1).
(DOC)

Figure S2 **Nucleotide sequence comparison of the serine transporter** ***sdaC*** **genes in different** ***C. jejuni*** **isolates.** The published sequences of the *sdaC* genes from several *C. jejuni* isolates were compared using ClustalW (www.ebi.ac.uk/Tools/msa/clustalW2). The sources for the nucleotide sequences were obtained from GenBank (NCBI) and have following accession numbers: 260.94 (CJJ26094_1676; NZ_AANK01000006), ICDCCJ07001 (ICDCCJ07001_1540; NC_014802), 81116 (C8J_1527; NC_009839), M1 (CJM1_1566; CP001900), 327(CSU_0678; ADHM01000033.1), DFVF1099 (CSQ_0900; ADHK01000020), 305 (CSS_1724; ADHL01000259.1), IA3902 (CJSA_1537; CP001876), NCTC11168 (Cj1625c; NC_002163), CG8486 (Cj8486_1667c; NZ_AASY01000001), 84-25 (CJJ8425_1709; NZ_AANT02000001), CF93-6 (CJJCF936_1719; NZ_AANJ01000002), CG8421 (Cj8421_1679; NZ_ABGQ01000002), ATCC 33251 (this study), RM1221 (CJE1797; NC_003912), S3 (CJS3_1706; CP001960), HB93-13 (CJJHB9313_1616; NZ_AANQ01000001), 81-176 (CJJ81176_1616; NC_008787), 1336 (C1336_000330074; NZ_ADGL01000024), 414 (C414_000010126; NZ_ADGM01000001).
(DOC)

Figure S3 **Amino acid sequence comparison of SdaA proteins from different** ***C. jejuni*** **strains.** ClustaW (www.ebi.ac.uk/Tools/msa/clustalW2) was used for the alignment of the SdaA amino acid sequences from various *C. jejuni* isolates. The accession numbers were as follows: CF93-6 (CJJCF936_1718; ZP_01067690), 84-25 (CJJ8425_1708; ZP_01099834), NCTC 11168 (Cj1624c; YP_002344993), IA3902 (CJSA_1536; ADC29171), DFVF1099 (CSQ_0902, this study), 305 (CSS_1725; this study), CG8486 (Cj8486_1666c; ZP_01809456), RM1221 (CJE1796; YP_179767), S3 (CJS3_1705; ADT73404), 260.94 (CJJ26094_1675; NZ_AANK01000006.1), 81-176 (CJJ81176_1615; YP_001001267), 1336 (C1336_000330073; ZP_06374482), HB93-13 (CJJHB9313_1615; ZP_01070837), M1 (CJM1_1565; ADN91750), 81116 (C8J_1526; YP_001483100), ICDCCJ07001 (ICDCCJ07001_1539; YP_004067035), 327 (CSU_0676; this study), CG8421 (Cj8421_1678; ZP_03222408), ATCC 33251 (this study), 414 (C414_000010127; ZP_06371291).
(DOC)

Figure S4 **Comparison of SdaC serine transporter** **protein sequences in various** ***C. jejuni*** **isolates.** ClustaW (www.ebi.ac.uk/Tools/msa/clustalW2) was used for the alignment of the SdaC amino acid sequences from listed *C. jejuni* isolates. The protein sequence accession numbers for the SdaC serine transporters of the different C. jejuni strains were as follows: RM1221 (CJE1797; YP_179768), S3 (CJS3_1706, ADT73405), CF93-6 (CJJCF936_1719; ZP_01067605), 84-25 (CJJ8425_1709; ZP_01099564); CG8486 (Cj8486_1667c; ZP_01809457), NCTC 11168 (Cj1625c; YP_002344994), IA3902 (CJSA_1537; ADC29172), DFVF1099 (CSQ_0900; EFV06958), 305 (CSS_1724; EFV08300); CG8421 (Cj8421_1679; ZP_03222409); ATCC 33251 (this study); 260.94 (CJJ26094_1676; ZP_01070445), HB93-13 (CJJHB9313_1616; ZP_01071370); 81-176 (CJJ81176_1616; ZP_02271921), 81116 (C8J_1527; YP_001483101), ICDCCJ07001 (ICDCCJ07001_1540; YP_004067036), M1 (CJM1_1566; ADN91751); 327 (CSU_0678; EFV10631), 1336 (C1336_000330074; ZP_06374483), 414 (C414_000010126; ZP_06371290).
(DOC)

Figure S5 **Growth of** ***C. jejuni*** **81-176 and its isogenic** ***sdaA*** **and** ***sdaC*** **mutants.** Growth characteristics of the *C. jejuni* 81-176 wild-type strain, its isogenic *sdaC* and *sdaA* mutants as well as a complemented *sdaA* mutant in DMEM and DMEM supplemented with 20 mM serine, glutamate or proline. The maximal optical densities (OD_600_) of liquid cultures from indicated *C. jejuni* strains over a time period of 24 hours are presented.
(TIF)

Figure S6 **Competitive index of mice co-infection with** ***C. jejuni*** **81-176 and its** ***sdaA*** **,** ***peb1A*** **or** ***putP*** **mutants.** The competitive index, CI = (mutant_output_/wild-type_output_)/(mutant_inoculum_/wild-type_inoculum_), was calculated for each mouse infected with *C. jejuni* 81-176 wild-type strain and its indicated mutant. The output numbers representing the CFUs of wild-type and mutant strains recovered from the intestine or the liver of each animal are plotted in Figures 3, 4 and 6. Each mouse was infected with approximately the same number of wild-type and mutant strain as determined by the CFU counting of the inoculum.
(TIF)

Figure S7 ***In vitro*** **growth competition experiments of** ***C. jejuni*** **81-176 and its mutants.** Shown are the co-cultivation experiments of *C. jejuni* 81-176 with its *peb1A*, *putP* and *sdaA* mutants, respectively. Equal amounts of wild-type and a mutant strain were incubated in nutrient rich BHI medium over night and the CFUs of each strain were determined after 20 hours. Each symbol represents the calculated competitive index for one co-cultivation experiment.
(TIF)

Figure S8 **Comparison of the** ***putA*** **/** ***putP*** **gene cluster in** ***Campylobacter*** **and** ***Enterobacteria*** **.** The schematic comparison of the *putAputP* gene locus and its flanking regions of several *Campylobacter* and *Enterobacter* strains was derived from the comparative genome database xBASE2 (Chaudhuri RR *et al*. (2008) Nucleic Acids Res., D543-6) in particular CampyDB (www.xbase.ac.uk/campydb) and ColiDB (www.xbase.ac.uk/colibase). The *putA* and *putP* genes are marked in light and dark grey, respectively. The genes of the flaking regions are represented as white arrows.
(TIF)

Table S1 **Proteobacteria with homologues to the L-serine dehydratase SdaA of** ***C. jejuni*** **81-176.** The table shows the homology between the SdaA protein of *C. jejuni* 81-176 and the SdaA proteins in other proteobacteria with their given accession numbers. The percent of amino acids identical and similar (conserved amino acid exchanges) between the serine ammonia-lyase of *C. jejuni* 81-176 and the other SdaA proteins were determined by BLASTP analysis (http://blast.ncbi.nlm.nih.gov/Blast.cgi). The order of the table reflects the score values calculated by the BLASTP algorithm. *C. jejuni* isolates are marked in red, other *Campylobacter* species in orange and *Helicobacter* species in yellow. Only a subset of *C. jejuni* isolates are listed, but all sequenced *C. jejuni* strains encode for SdaA homologues that are 100% or 99% identical to the SdaA protein of *C. jejuni* 81-176.
(DOC)

Table S2 **Proteobacteria with homologues to the serine transporter SdaC of** ***C. jejuni*** **81-176.** The table shows the homology between the SdaC protein of *C. jejuni* 81-176 and the SdaC proteins in other proteobacteria. The order represents the grade of homology according to the score calculated by the BlastP algorithm (http://blast.ncbi.nlm.nih.gov/Blast.cgi). *C. jejuni* isolates are marked in red other *Campylobacter* species in orange and *Helicobacter* species in yellow. Sequenced *C. jejuni* strains that are not represented in the table encode for SdaC homologues that are at least 99% identical to the SdaC of *C. jejuni* 81-176.
(DOC)

Table S3 **Proteobacteria with homologues to the proline dehydrogenase/delta 1-pyrroline-5-carboxylate dehydrogenase PutA of** ***C. jejuni*** **81-176.** The table shows the homology between the PutA protein of *C. jejuni* 81-176 and the PutA proteins in other proteobacteria with their given accession numbers. The percent of amino acids identical and similar (conserved amino acid exchanges) between *C. jejuni* 81-176 PutA and the PutA proteins of other presented proteobacteria were determined by BLASTP analysis (http://blast.ncbi.nlm.nih.gov/Blast.cgi). The order of the table reflects the score values calculated by the BLASTP algorithm. *C. jejuni* isolates are marked in red, other *Campylobacter* species in orange and *Helicobacter* species in yellow. Only a subset of *C. jejuni* isolates are listed, but all sequenced *C. jejuni* strains encode for SdaA homologues that are at least 99% identical to the SdaA protein of *C. jejuni* 81-176.
(DOC)

Table S4 **Proteobacteria with homologues to the proline transporter PutP of** ***C. jejuni*** **81-176.** The table illustrates the homology between the PutP protein of *C. jejuni* 81-176 and the PutP proteins in other proteobacteria. The accession numbers of the PutP proteins from indicated bacteria are listed. The order represents the grade of homology according to the score calculated by the BlastP algorithm (http://blast.ncbi.nlm.nih.gov/Blast.cgi). *C. jejuni* isolates are marked in red, *Campyobacter* species besides *C. jejuni* are highlighted in orange and *Helicobacter* species in yellow. Not all *C. jejuni* isolates are listed, but every sequenced *C. jejuni* strain encodes for a PutP homologue that is at least 98% identical to the PutP protein of *C. jejuni* 81-176.
(DOC)

Table S5 **L-serine dehydratase activity in cell extracts of** ***C. jejuni*** **isolates.** The mean serine dehydratase activities with standard deviations are shown for measurements repeated four (a) and three (b) times in triplicates. The statistical significance in the differences of SdaA activity between *C. jejuni* 81-176 and *C. jejuni* 33251 or RM1221 was calculated by Student *t* test: * P<0.01; ** P<0.05.
(DOC)

Table S6 **Primers used in this study.**
(DOC)

Table S7 **Strains and plasmids used in this study.**
(DOC)
