# Supplementary material for: Contribution of Amino Acid Catabolism to the Tissue Specific Persistence of Campylobacter jejuni in a Murine Colonization Model
Source: PLoS One. 2012 Nov 30;7(11):e50699. doi: 10.1371/journal.pone.0050699 (PMC3511319; doi:10.1371/journal.pone.0050699)
Supplement: Figure S1 — Nucleotide sequence comparison of the L-serine dehydratase sdaA genes in indifferent C. jejuni isolates. The nucleotide sequences of sdaA open reading frames from different C. jejuni strains were compared using ClustalW (www.ebi.ac.uk/Tools/msa/clustalW2). The sources for the DNA sequences are as follows: CG8486 (Cj8486_1666c; NZ_AASY01000001.2), ATCC 33251 (this study), CF93-6 (CJJCF936_1718; NZ_AANJ01000002.1), 84-25 (CJJ8425_1708; NZ_AANT02000001.1), NCTC 11168 (Cj1624c; NC_002163.1), IA3902 (CJSA_1536; CP001876.1), DFVF1099 (CSQ_0902; ADHK01000020.1; this study), 305 (CSS_1725; ADHL01000259.1; this study), RM1221 (CJE1796; NC_003912.7), S3 (CJS3_1705; CP001960.1), 260.94 (CJJ26094_1675; NZ_AANK01000006.1), ICDCCJ07001 (ICDCCJ07001_1539; NC_014802.1), HB93-13 (CJJHB9313_1615; NZ_AANQ01000001.1), 81-176 (CJJ81176_1615; AASL01000001.1), 81116 (C8J_1526; NC_009839.1), M1 (CJM1_1565; CP001900.1), 327 (CSU_0676; ADHM01000033.1; this study), 1336 (C1336_000330073; NZ_ADGL01000024.1), CG8421 (Cj8421_1678; NZ_ABGQ01000002.1), 414 (C414_000010127; NZ_ADGM01000001.1). (DOC) [file pone.0050699.s001.doc]

CG8486 ATGAGTAATTTAAGCATTTTTAAAATAGGTGTTGGCCCTTCTTCTTCTCATACTTTAGGG 60

**ATCC 33251** ATGAGTAATTTAAACATTTTTAAAATAGGTGTTGGCCCTTCTTCTTCTCATACTTTAGGG 60

CF93-6 ATGAGTAATTTAAGCATTTTTAAAATAGGTGTTGGCCCTTCTTCTTCTCATACTTTAGGG 60

84-25 ATGAGTAATTTAAGCATTTTTAAAATAGGTGTTGGCCCTTCTTCTTCTCATACTTTAGGG 60

NCTC 11168 ATGAGTAATTTAAGCATTTTTAAAATAGGTGTTGGCCCTTCTTCTTCTCATACTTTAGGG 60

IA3902 ATGAGTAATTTAAGCATTTTTAAAATAGGTGTTGGCCCTTCTTCTTCTCATACTTTAGGG 60

DFVF1099 ATGAGTAATTTAAGCATTTTTAAAATAGGTGTTGGCCCTTCTTCTTCTCATACTTTAGGG 60

305 ATGAGTAATTTAAGCATTTTTAAAATAGGTGTTGGCCCTTCTTCTTCTCATACTTTAGGG 60

RM1221 ATGAGTAATTTAAGCATTTTTAAAATAGGTGTTGGTCCTTCTTCTTCTCATACTTTAGGG 60

S3 ATGAGTAATTTAAGCATTTTTAAAATAGGTGTTGGTCCTTCTTCTTCTCATACTTTAGGG 60

260.94 ATGAGTAATTTAAGCATTTTTAAAATAGGTGTTGGTCCTTCTTCTTCTCATACTTTAGGG 60

ICDCCJ07001 ATGAGTAATTTAAGCATTTTTAAAATAGGTGTTGGTCCTTCTTCTTCTCATACTTTAGGG 60

HB93-13 ATGAGTAATTTAAGCATTTTTAAAATAGGTGTTGGTCCTTCTTCTTCTCATACTTTAGGG 60

81-176 ATGAGTAATTTAAGCATTTTTAAAATAGGTGTTGGTCCTTCTTCTTCTCATACTTTAGGG 60

81116 ATGAGTAATTTAAGCATTTTTAAAATAGGTGTTGGTCCTTCTTCTTCTCATACTTTAGGG 60

M1 ATGAGTAATTTAAGCATTTTTAAAATAGGTGTTGGTCCTTCTTCTTCTCATACTTTAGGG 60

327 ATGAGTAATTTAAGCATTTTTAAAATAGGTGTTGGTCCTTCTTCTTCTCATACTTTAGGG 60

1336 ATGAGTAATTTAAGCATTTTTAAAATAGGTGTCGGTCCTTCTTCTTCTCATACTTTAGGG 60

CG8421 ATGAGTAATTTAAGCATTTTTAAAATAGGTGTTGGCCCTTCTTCTTCTCATACTTTAGGG 60

414 ATGAGCAATTTAAGCATTTTTAAAATAGGTGTTGGTCCTTCGTCTTCTCATACTTTAGGG 60

***** ******* ****************** ** ***** ******************

CG8486 CCTATGCTTGCTGGAAATTTATTTTGTAAAAAAGTTGCTAAAAAGCTTGATGAAATTGAT 120

**ATCC 33251** CCTATGCTTGCTGGAAATTTATTTTGTAAAAAAGTTGCTAAAAAGCTTGATGAAATTGAT 120

CF93-6 CCTATGCTTGCTGGAAATTTATTTTGTAAAAAAGTTGCTAAAAAGCTTGATGAAATTGAT 120

84-25 CCTATGCTTGCTGGAAATTTATTTTGTAAAAAAGTTGCTAAAAAGCTTGATGAAATTGAT 120

NCTC 11168 CCTATGCTTGCTGGAAATTTATTTTGTAAAAAAGTTGCTAAAAAGCTTGATGAAATTGAT 120

IA3902 CCTATGCTTGCTGGAAATTTATTTTGTAAAAAAGTTGCTAAAAAGCTTGATGAAATTGAT 120

DFVF1099 CCTATGCTTGCTGGAAATTTATTTTGTAAAAAAGTTGCTAAAAAGCTTGATGAAATTGAT 120

305 CCTATGCTTGCTGGAAATTTATTTTGTAAAAAAGTTGCTAAAAAGCTTGATGAAATTGAT 120

RM1221 CCTATGCTTGCTGGAAATTTATTTTGTAAAAAAGTCGCTAAAAAGCTTGATGAAATTGAT 120

S3 CCTATGCTTGCTGGAAATTTATTTTGTAAAAAAGTCGCTAAAAAGCTTGATGAAATTGAT 120

260.94 CCTATGCTTGCTGGAAATTTATTTTGTAAAAAAGTCGCTAAAAAGCTTGATGAAATTGAT 120

ICDCCJ07001 CCTATGCTTGCTGGAAATTTATTTTGTAAAAAAGTCGCTAAAAAGCTTGATGAAATTGAT 120

HB93-13 CCTATGCTTGCTGGAAATTTATTTTGTAAAAAAGTCGCTAAAAAGCTTGATGAAATTGAT 120

81-176 CCTATGCTTGCTGGAAATTTATTTTGTAAAAAAGTCGCTAAAAAGCTTGATGAAATTGAT 120

81116 CCTATGCTTGCTGGAAATTTATTTTGTAAAAAAGTCGCTAAAAAGCTTGATGAAATTGAT 120

M1 CCTATGCTTGCTGGAAATTTATTTTGTAAAAAAGTCGCTAAAAAGCTTGATGAAATTGAT 120

327 CCTATGCTTGCTGGAAATTTATTTTGTAAAAAAGTCGCTAAAAAGCTTGATGAAATTGAT 120

1336 CCTATGCTTGCCGGAAATTTATTTTGTAAAAAAGTTGCTAAAAAGCTTGATGAAATTGAT 120

CG8421 CCTATGCTTGCTGGAAATTTATTTTGTAAAAAAGTTGCTAAAAAGCTTGATGAAATTGAT 120

414 CCTATGCTTGCTGGAAATTTATTTTGTAAAAAAGTTGCTAAAAAGCTTGATGAAATCAAT 120

*********** *********************** ******************** **

CG8486 AGAGTTGAGGTTACTCTTTATGGTTCTTTGTCGTTAACAGGCAAAGGACACTTGAGTGAT 180

**ATCC 33251** AGAGTTGAGGTTACTCTTTATGGTTCTTTGTCGTTAACAGGCAAAGGACACTTGAGTGAT 180

CF93-6 AGAGTTGAGGTTACTCTTTATGGTTCTTTGTCGTTAACAGGCAAAGGACACTTGAGTGAT 180

84-25 AGAGTTGAGGTTACTCTTTATGGTTCTTTGTCGTTAACAGGCAAAGGACACTTGAGTGAT 180

NCTC 11168 AGAGTTGAGGTTACTCTTTATGGTTCTTTGTCGTTAACAGGCAAAGGACACTTGAGTGAT 180

IA3902 AGAGTTGAGGTTACTCTTTATGGTTCTTTGTCGTTAACAGGCAAAGGACACTTGAGTGAT 180

DFVF1099 AGAGTTGAGGTTACTCTTTATGGTTCTTTGTCGTTAACAGGCAAAGGACACTTGAGTGAT 180

305 AGAGTTGAGGTTACTCTTTATGGTTCTTTGTCGTTAACAGGCAAAGGACACTTGAGTGAT 180

RM1221 AGAGTTGAGGTTACTCTTTATGGTTCTTTGTCATTAACAGGTAAAGGACACTTGAGTGAT 180

S3 AGAGTTGAGGTTACTCTTTATGGTTCTTTGTCATTAACAGGTAAAGGACACTTGAGTGAT 180

260.94 AGAGTTGAGGTTACTCTTTATGGTTCTTTGTCATTAACAGGTAAAGGACACTTGAGTGAT 180

ICDCCJ07001 AGAGTTGAGGTTACTCTTTATGGTTCTTTGTCATTAACAGGTAAAGGACACTTGAGTGAT 180

HB93-13 AGAGTTGAGGTTACTCTTTATGGTTCTTTGTCATTAACAGGTAAAGGACACTTGAGTGAT 180

81-176 AGAGTTGAGGTTACTCTTTATGGTTCTTTGTCATTAACAGGTAAAGGACACTTGAGTGAT 180

81116 AGAGTTGAGGTTACTCTTTATGGTTCTTTGTCATTAACAGGTAAAGGACACTTGAGTGAT 180

M1 AGAGTTGAGGTTACTCTTTATGGTTCTTTGTCATTAACAGGTAAAGGACACTTGAGTGAT 180

327 AGAGTTGAGGTTACTCTTTATGGTTCTTTGTCATTAACAGGTAAAGGACACTTGAGTGAT 180

1336 AGAGTTGAGGTTACTCTTTATGGTTCTTTGTCATTAACAGGTAAAGGACACTTGAGTGAT 180

CG8421 AGAGTTGAGGTTACTCTTTATGGTTCTTTGTCGTTAACAGGCAAAGGACACTTGAGTGAT 180

414 AGGGTTGAAGTTACTCTTTATGGTTCTTTGTCGTTAACGGGCAAGGGGCACTTGAGCGAT 180

** ***** *********************** ***** ** ** ** ******** ***

CG8486 AAGGCTGTGATTTGGGGGTTAAATGGCTTAGAAGCTAAAAATTTAAGTGCGGCAATACAA 240

**ATCC 33251** AAGGCTGTGATTTGGGGGTTAAATGGCTTAGAAGCTAAAAATTTAAGTGCAGCAATACAA 240

CF93-6 AAGGCTGTGATTTGGGGGTTAAATGGCTTAGAAGCTAAAAATTTAAGTGCGGCAATACAA 240

84-25 AAGGCTGTGATTTGGGGGTTAAATGGCTTAGAAGCTAAAAATTTAAGTGCGGCAATACAA 240

NCTC 11168 AAGGCTGTGATTTGGGGGTTAAATGGCTTAGAAGCTAAAAATTTAAGTGCGGCAATACAA 240

IA3902 AAGGCTGTGATTTGGGGGTTAAATGGCTTAGAAGCTAAAAATTTAAGTGCGGCAATACAA 240

DFVF1099 AAGGCTGTGATTTGGGGGTTAAATGGCTTAGAAGCTAAAAATTTAAGTGCGGCAATACAA 240

305 AAGGCTGTGATTTGGGGGTTAAATGGCTTAGAAGCTAAAAATTTAAGTGCGGCAATACAA 240

RM1221 AAGGCTGTAATTTGGGGGTTAAATGGCCTAGAAGCTAAAAATTTAAGCACAGCAATACAA 240

S3 AAGGCTGTAATTTGGGGGTTAAATGGCCTAGAAGCTAAAAATTTAAGCACAGCAATACAA 240

260.94 AAGGCTGTAATTTGGGGGTTAAATGGCCTAGAAGCTAAAAATTTAAGCGCAGCAATACAA 240

ICDCCJ07001 AAGGCTGTAATTTGGGGGTTAAATGGCCTAGAAGCTAAAAATTTAAGCGCAGCAATACAA 240

HB93-13 AAGGCTGTAATTTGGGGGTTAAATGGCCTAGAAGCTAAAAATTTAAGCGCAGCAATACAA 240

81-176 AAGGCTGTAATTTGGGGGTTAAATGGCCTAGAAGCTAAAAATTTAAGCGCAGCAATACAA 240

81116 AAGGCTGTAATTTGGGGGTTAAATGGCCTAGAAGCTAAAAATTTAAGCGCAGCAATACAA 240

M1 AAGGCTGTAATTTGGGGGTTAAATGGCCTAGAAGCTAAAAATTTAAGCGCAGCAATACAA 240

327 AAGGCTGTAATTTGGGGGTTAAATGGCCTAGAAGCTAAAAATTTAAGCGCAGCAATACAA 240

1336 AAGGCTGTAATTTGGGGGTTAAATGGCCTAGAAGCTAAAAATTTAAGCGCAGCAATACAA 240

CG8421 AAGGCTGTGATTTGGGGGTTAAATGGCTTAGAAGCTAAAAATTTAAGTGCGGCAATACAA 240

414 AAGGCTGTAATTTGGGGGTTAAATGGTTTAGAGGCTAAAAACTTAAGTGCAGCAATACAA 240

******** ***************** **** ******** ***** * *********

CG8486 GATGAAGTAAATAAAAATGCTATTGAAAATGCTCAAATTGATTTTTGTGGCGAGAAAAAA 300

**ATCC 33251** GATGAAGTAAATAAAAATGCTATTGAAAATGCTCAAATTGATTTTTGTGGCGAGAAAAAA 300

CF93-6 GATGAAGTAAATAAAAATGCTATTGAAAATGCTCAAATTGATTTTTGTGGCGAGAAAAAA 300

84-25 GATGAAGTAAATAAAAATGCTATTGAAAATGCTCAAATTGATTTTTGTGGCGAGAAAAAA 300

NCTC 11168 GATGAAGTAAATAAAAATGCTATTGAAAATGCTCAAATTGATTTTTGTGGCGAGAAAAAA 300

IA3902 GATGAAGTAAATAAAAATGCTATTGAAAATGCTCAAATTGATTTTTGTGGCGAGAAAAAA 300

DFVF1099 GATGAAGTAAATAAAAATGCTATTGAAAATGCTCAAATTGATTTTTGTGGCGAGAAAAAA 300

305 GATGAAGTAAATAAAAATGCTATTGAAAATGCTCAAATTGATTTTTGTGGCGAGAAAAAA 300

RM1221 GATGAAGTAAATAAAAATGCTATTGAAAATGCTCAAATTGATTTTTGTGGTGAGAAAAAA 300

S3 GATGAAGTAAATAAAAATGCTATTGAAAATGCTCAAATTGATTTTTGTGGTGAGAAAAAA 300

260.94 GATGAAGTAAATAAAAATGCTATTGAAAATGCTCAAATTGATTTTTGTGGTGAGAAAAAA 300

ICDCCJ07001 GATGAAGTAAATAAAAATGCTATTGAAAATGCTCAAATTGATTTTTGTGGTGAGAAAAAA 300

HB93-13 GATGAAGTAAATAAAAATGCTATTGAAAATGCTCAAATTGATTTTTGTGGTGAGAAAAAA 300

81-176 GATGAAGTAAATAAAAATGCTATTGAAAATGCTCAAATTGATTTTTGTGGTGAGAAAAAA 300

81116 GATGAAGTAAATAAAAATGCTATTGAAAATGCTCAAATTGATTTTTGTGGTGAGAAAAAA 300

M1 GATGAAGTAAATAAAAATGCTATTGAAAATGCTCAAATTGATTTTTGTGGTGAGAAAAAA 300

327 GATGAAGTAAATAAAAATGCTATTGAAAATGCTCAAATTGATTTTTGTGGTGAGAAAAAA 300

1336 GATGAAGTAAATAAAAATGCTATTGAAAATGCTCAAATTGATTTTTGTGGTGAGAAAAAA 300

CG8421 GATGAAGTAAATAAAAATGCTATTGAAAATGCTCAAATTGATTTTTGTGGTGAGAAAAAA 300

414 GATGAAGTGAATAAAAATGCTATTGAAAATGCTCAAATTGATTTTTGCGGTGAGAAAAAA 300

******** ************************************** ** *********

CG8486 CTTAGTTTTAATTATGAAAAAGATTTGATATTTTCTAAAGATTTTTTACCTTTACATG-- 358

**ATCC 33251** CTTAGTTTTAATTATGAAAAAGATTTGATATTTTCTAAAGATTTTTTACCTTTACATGTA 360

CF93-6 CTTAGTTTTAATTATGAAAAAGATTTGATATTTTCTAAAGATTTTTTACCTTTACATG-- 358

84-25 CTTAGTTTTAATTATGAAAAAGATTTGATATTTTCTAAAGATTTTTTACCTTTACATG-- 358

NCTC 11168 CTTAGTTTTAATTATGAAAAAGATTTGATATTTTCTAAAGATTTTTTACCTTTACATG-- 358

IA3902 CTTAGTTTTAATTATGAAAAAGATTTGATATTTTCTAAAGATTTTTTACCTTTACATG-- 358

DFVF1099 CTTAGTTTTAATTATGAAAAAGATTTGATATTTTCTAAAGATTTTTTACCTTTACATG-- 358

305 CTTAGTTTTAATTATGAAAAAGATTTGATATTTTCTAAAGATTTTTTACCTTTACATG-- 358

RM1221 CTTTGTTTTAATTATGAAAAAGATTTGATATTTTCCAAAGATTTTTTACCTTTACATG-- 358

S3 CTTTGTTTTAATTATGAAAAAGATTTGATATTTTCCAAAGATTTTTTACCTTTACATG-- 358

260.94 CTTTGTTTTAATTATGAAAAAGATTTGATATTTTCCAAAGATTTTTTACCTTTACATG-- 358

ICDCCJ07001 CTTTGTTTTAATTATGAAAAAGATTTGATATTTTCCAAAGATTTTTTACCTTTACATG-- 358

HB93-13 CTTTGTTTTAATTATGAAAAAGATTTGATATTTTCCAAAGATTTTTTACCTTTACATG-- 358

81-176 CTTTGTTTTAATTATGAAAAAGATTTGATATTTTCCAAAGATTTTTTACCTTTACATG-- 358

81116 CTTTGTTTTAATTATGAAAAAGATTTGATATTTTCCAAAGATTTTTTACCTTTACATG-- 358

M1 CTTTGTTTTAATTATGAAAAAGATTTGATATTTTCCAAAGATTTTTTACCTTTACATG-- 358

327 CTTTGTTTTAATTATGAAAAAGATTTGATATTTTCCAAAGATTTTTTACCTTTACATG-- 358

1336 CTTTGTTTTAATTATGAAAAAGATTTGATATTTTCCAAAGATTTTTTACCTTTACATG-- 358

CG8421 CTTTGTTTTAATTATGAAAAAGATTTGATATTTTCCAAAGATTTTTTACCTTTACATG-- 358

414 CTTAGTTTTAATTATGAAAAAGATTTGATATTTTCCAAAGATTTCTTACCTTTACATG-- 358

*** ******************************* ******** *************

CG8486 ----AGAATGGTATGAAAATTAAAGCTTATGATTGTAAAGGTGGGTTAGTTGATGAGGAA 414

**ATCC 33251** CATGAGAATGGTATGAAAATTAAAGCTTATGATTGTAAAGGTGGGTTAGTTGATGAGGAA 420

CF93-6 ----AGAATGGTATGAAAATTAAAGCTTATGATTGTAAAGGTGGGTTAGTTGATGAGGAA 414

84-25 ----AGAATGGTATGAAAATTAAAGCTTATGATTGTAAAGGTGGGTTAGTTGATGAGGAA 414

NCTC 11168 ----AGAATGGTATGAAAATTAAAGCTTATGATTGTAAAGGTGGGTTAGTTGATGAGGAA 414

IA3902 ----AGAATGGTATGAAAATTAAAGCTTATGATTGTAAAGGTGGGTTAGTTGATGAGGAA 414

DFVF1099 ----AGAATGGTATGAAAATTAAAGCTTATGATTGTAAAGGTGGGTTAGTTGATGAGGAA 414

305 ----AGAATGGTATGAAAATTAAAGCTTATGATTGTAAAGGTGGGTTAGTTGATGAGGAA 414

RM1221 ----AGAATGGTATGAAGATTAAAGCTTATGATTGTAAAGGTGGGTTAGTTGATGAAGAA 414

S3 ----AGAATGGTATGAAGATTAAAGCTTATGATTGTAAAGGTGGGTTAGTTGATGAAGAA 414

260.94 ----AGAATGGTATGAAGATTAAAGCTTATGATTGTAAAGGCGGGTTAGTTGATGAAGAA 414

ICDCCJ07001 ----AGAATGGTATGAAGATTAAAGCTTATGATTGTAAAGGCGGGTTAGTTGATGAAGAA 414

HB93-13 ----AGAATGGTATGAAGATTAAAGCTTATGATTGTAAAGGTGGGTTAGTTGATGAAGAA 414

81-176 ----AGAATGGTATGAAGATTAAAGCTTATGATTGTAAAGGTGGGTTAGTTGATGAAGAA 414

81116 ----AGAATGGTATGAAGATTAAAGCTTATGATTGTAAAGGTGGGTTAGTCGATGAAGAA 414

M1 ----AGAATGGTATGAAGATTAAAGCTTATGATTGTAAAGGTGGGTTAGTCGATGAAGAA 414

327 ----AGAATGGTATGAAGATTAAAGCTTATGATTGTAAAGGTGGGTTAGTCGATGAAGAA 414

1336 ----AGAATGGTATGAAGATTAAAGCTTATGATTGTAAAGGTGGGTTAGTTGATGAAGAA 414

CG8421 ----AGAATGGTATGAAGATTAAAGCTTATGATTGTAAAGGTGGGTTAGTTGATGAGGAA 414

414 ----AGAATGGTATGAAAATTAAAGCTTATGATTGCAAAGGTGCATTAGTTGATGAAGAA 414

************* ***************** ***** * ***** ***** ***

CG8486 ACTTATTATTCTGTAGGTGGAGGCTTTGTTTTAACAGCTGCGGAATTAGAAAAAAAAGGT 474

**ATCC 33251** ACTTATTATTCTGTAGGTGGAGGCTTTGTTTTAACAGCTGCGGAATTAGAAAAAAAAGGT 480

CF93-6 ACTTATTATTCTGTAGGTGGAGGCTTTGTTTTAACAGCTGCGGAATTAGAAAAAAAAGGT 474

84-25 ACTTATTATTCTGTAGGTGGAGGCTTTGTTTTAACAGCTGCGGAATTAGAAAAAAAAGGT 474

NCTC 11168 ACTTATTATTCTGTAGGTGGAGGCTTTGTTTTAACAGCTGCGGAATTAGAAAAAAAAGGT 474

IA3902 ACTTATTATTCTGTAGGTGGAGGCTTTGTTTTAACAGCTGCGGAATTAGAAAAAAAAGGT 474

DFVF1099 ACTTATTATTCTGTAGGTGGAGGCTTTGTTTTAACAGCTGCGGAATTAGAAAAAAAAGGT 474

305 ACTTATTATTCTGTAGGTGGAGGCTTTGTTTTAACAGCTGCGGAATTAGAAAAAAAAGGT 474

RM1221 ACTTATTATTCTGTAGGCGGAGGCTTTGTTTTAACAGCTGCACAATTAGAAAAAAAAGGT 474

S3 ACTTATTATTCTGTAGGCGGAGGCTTTGTTTTAACAGCTGCACAATTAGAAAAAAAAGGT 474

260.94 ACTTATTATTCTGTAGGTGGAGGCTTTGTTTTAACAGCTGCACAATTAGAAAAAAAAGGT 474

ICDCCJ07001 ACTTATTATTCTGTAGGTGGAGGCTTTGTTTTAACAGCTGCACAATTAGAAAAAAAAGGT 474

HB93-13 ACTTATTATTCTGTAGGTGGAGGCTTTGTTTTAACAGCTGCACAATTAGAAAAAAAAGGT 474

81-176 ACTTATTATTCTGTAGGTGGAGGCTTTGTTTTAACAGCTGCACAATTAGAAAAAAAAGGT 474

81116 ACTTATTATTCTGTAGGTGGAGGCTTTGTTTTAACAGCTGCACAATTAGAAAAAAAAGGT 474

M1 ACTTATTATTCTGTAGGTGGAGGCTTTGTTTTAACAGCTGCACAATTAGAAAAAAAAGGT 474

327 ACTTATTATTCTGTAGGTGGAGGCTTTGTTTTAACAGCTGCACAATTAGAAAAAAAAGGT 474

1336 ACTTATTATTCTGTAGGTGGAGGCTTTGTTTTAACAGCTGCACAATTAGAAAAAAAAGGT 474

CG8421 ACTTATTATTCTGTAGGTGGAGGCTTTGTTTTAACAGCTGCGGAATTAGAAAAAAAAGAT 474

414 ACTTATTATTCTGTAGGCGGTGGCTTTGTTTTAACAGCTGCGGAATTAGAAAAAGAAGGT 474

***************** ** ******************** *********** *** *

CG8486 AAAAATTCTAACCAAAATAAAAAGAAAAAACTAGATATAGAGCTTAATAACGCTAAAGAA 534

**ATCC 33251** AAAAATTCTAACCAAAATAAAAAGAAAAAACTAGATATAGAGCTTAATAACGCTAAAGAA 540

CF93-6 AAAAATTCTAACCAAAATAAAAAGAAAAAACTAGATATAGAGCTTAATAACGCTAAAGAA 534

84-25 AAAAATTCTAACCAAAATAAAAAGAAAAAACTAGATATAGAGCTTAATAACGCTAAAGAA 534

NCTC 11168 AAAAATTCTAACCAAAATAAAAAGAAAAAACTAGATATAGAGCTTAATAACGCTAAAGAA 534

IA3902 AAAAATTCTAACCAAAATAAAAAGAAAAAACTAGATATAGAGCTTAATAACGCTAAAGAA 534

DFVF1099 AAAAATTCTAACCAAAATAAAAAGAAAAAACTAGATATAGAGCTTAATAACGCTAAAGAA 534

305 AAAAATTCTAACCAAAATAAAAAGAAAAAACTAGATATAGAGCTTAATAACGCTAAAGAA 534

RM1221 AAAAATTCCAACCAAAATAAAAAGAAAAAACTAGATATAGAGCTTAACAATGCAAAAGAA 534

S3 AAAAATTCCAACCAAAATAAAAAGAAAAAACTAGATATAGAGCTTAACAATGCAAAAGAA 534

260.94 AAAAATTCCAACCAAAATAAAAAGAAAAAACTAGATATAGAGCTTAACAATGCAAAAGAA 534

ICDCCJ07001 AAAAATTCCAACCAAAATAAAAAGAAAAAACTAGATATAGAGCTTAACAATGCAAAAGAA 534

HB93-13 AAAAATTCCAATCAAAATAAAAAGAAAAAACTAGATATAGAGCTTAACAATGCAAAAGAA 534

81-176 AAAAATTCCAATCAAAATAAAAAGAAAAAACTAGATATAGAGCTTAACAATGCAAAAGAA 534

81116 AAAAATTCCAACCAAAATAAAAAGAAAAAACTAGATATAGAGCTTAACAATGCAAAAGAA 534

M1 AAAAATTCCAACCAAAATAAAAAGAAAAAACTAGATATAGAGCTTAACAATGCAAAAGAA 534

327 AAAAATTCCAACCAAAATAAAAAGAAAAAACTAGATATAGAGCTTAACAATGCAAAAGAA 534

1336 AAAAATTCCAACCAAAATAAAAAGAAAAAACTAGATATAGAGCTTAACAATGCAAAAGAA 534

CG8421 AAAAATTCCAACCAAAATAAAAAGAAAAAACTAGATATAGAGCTTAACAATGCAAAAGAA 534

414 AAAAATTCTAATCAAAACAAAAAGAAAAAACTAGACATAGAGCTTAATAATGCAAAAGAA 534

******** ** ***** ***************** *********** ** ** ******

CG8486 GCTTTAGAGCTTTGCGATAAAAGAGATTGGGATTTAGCAGAGCTTTCCTATCGTTATGAA 594

**ATCC 33251** GCTTTAGAGCTTTGCGATAAAAGAGATTGGGATTTAGCAGAGCTTTCCTATCGTTATGAA 600

CF93-6 GCTTTAGAGCTTTGCGATAAAAGAGATTGGGATTTGGCAGAGCTTTCTTATCGTTATGAA 594

84-25 GCTTTAGAGCTTTGCGATAAAAGAGATTGGGATTTGGCAGAGCTTTCTTATCGTTATGAA 594

NCTC 11168 GCTTTAGAGCTTTGCGATAAAAGAGATTGGGATTTGGCAGAGCTTTCTTATCGTTATGAA 594

IA3902 GCTTTAGAGCTTTGCGATAAAAGAGATTGGGATTTGGCAGAGCTTTCTTATCGTTATGAA 594

DFVF1099 GCTTTAGAGCTTTGCGATAAAAGAGATTGGGATTTGGCAGAGCTTTCTTATCGTTATGAA 594

305 GCTTTAGAGCTTTGCGATAAAAGAGATTGGGATTTGGCAGAGCTTTCTTATCGTTATGAA 594

RM1221 GCTTTAGAGCTTTGCGATAAAAGAGATTGGGATTTAGCAGAGCTTTCTTATCGTTACGAA 594

S3 GCTTTAGAGCTTTGCGATAAAAGAGATTGGGATTTAGCAGAGCTTTCTTATCGTTACGAA 594

260.94 GCTTTAGAGCTTTGCGATAAAAGAGATTGGGATTTAGCAGAGCTTTCTTATCGTTATGAA 594

ICDCCJ07001 GCTTTAGAGCTTTGCGATAAAAGAGATTGGGATTTAGCAGAGCTTTCTTATCGTTATGAA 594

HB93-13 GCTTTAGAGCTTTGCGATAAAAGAGATTGGGATTTAGCAGAGCTTTCTTATCGTTATGAA 594

81-176 GCTTTAGAGCTTTGCGATAAAAGAGATTGGGATTTAGCAGAGCTTTCTTATCGTTATGAA 594

81116 GCTTTAGAGCTTTGCGATAAAAGAGATTGGGATTTAGCAGAGCTTTCTTATCGTTATGAA 594

M1 GCTTTAGAGCTTTGCGATAAAAGAGATTGGGATTTAGCAGAGCTTTCTTATCGTTATGAA 594

327 GCTTTAGAGCTTTGCGATAAAAGAGATTGGGATTTAGCAGAGCTTTCTTATCGTTATGAA 594

1336 GCTTTAGAGCTTTGCGATAAAAGAGATTGGGATTTAGCAGAGCTTTCTTATCGTTATGAA 594

CG8421 GCTTTAGAGCTTTGCGATAAAAGAGATTGGGATTTAGCAGAGCTTTCTTATCGTTACGAA 594

414 GCTTTAGAACTTTGTGATAAAAGAGATTGGGATTTAGCAGAGCTTTCTTATCGTTATGAA 594

******** ***** ******************** *********** ******** ***

CG8486 TTGCAATTTCACACTAAAGAGGAAATTCGTGCTTATTGTCTTGAAATTTGGGAAGTGATG 654

**ATCC 33251** TTGCAATTTCACACTAAAGAGGAAATTCGTGCTTATTGTCTTGAAATTTGGGAAGTGATG 660

CF93-6 TTGCAATTTCACACTAAAGAGGAAATTTGTGCTTATTGTCTTGAAATTTGGGAAGTGATG 654

84-25 TTGCAATTTCACACTAAAGAGGAAATTTGTGCTTATTGTCTTGAAATTTGGGAAGTGATG 654

NCTC 11168 TTGCAATTTCACACTAAAGAGGAAATTTGTGCTTATTGTCTTGAAATTTGGGAAGTGATG 654

IA3902 TTGCAATTTCACACTAAAGAGGAAATTTGTGCTTATTGTCTTGAAATTTGGGAAGTGATG 654

DFVF1099 TTGCAATTTCACACTAAAGAGGAAATTTGTGCTTATTGTCTTGAAATTTGGGAAGTGATG 654

305 TTGCAATTTCACACTAAAGAGGAAATTTGTGCTTATTGTCTTGAAATTTGGGAAGTGATG 654

RM1221 TTGCAATTTCACACTAAAGAGGAAATTCGTGCTTATTGTCTTGAAATTTGGGAAGTAATG 654

S3 TTGCAATTTCACACTAAAGAGGAAATTCGTGCTTATTGTCTTGAAATTTGGGAAGTAATG 654

260.94 TTGCAATTTCACACTAAAGAGGAAATTCGTGCTTATTGTCTTGAAATTTGGGAAGTAATG 654

ICDCCJ07001 TTGCAATTTCACACTAAAGAGGAAATTCGTGCTTATTGTCTTGAAATTTGGGAAGTAATG 654

HB93-13 TTGCAATTTCACACTAAAGAGGAAATTCGTGCTTATTGTCTTGAAATTTGGGAAGTAATG 654

81-176 TTGCAATTTCACACTAAAGAGGAAATTCGTGCTTATTGTCTTGAAATTTGGGAAGTAATG 654

81116 TTGCAATTTCACACTAAAGAGGAAATTCGTGCTTATTGTCTTGAAATTTGGGAAGTAATG 654

M1 TTGCAATTTCACACTAAAGAGGAAATTCGTGCTTATTGTCTTGAAATTTGGGAAGTAATG 654

327 TTGCAATTTCACACTAAAGAGGAAATTCGTGCTTATTGTCTTGAAATTTGGGAAGTAATG 654

1336 TTGCAATTTCACACTAAAGAGGAAATTCGTGCTTATTGTCTTGAAATTTGGGAAGTAATG 654

CG8421 TTGCAATTTCACACTAAAGAGGAAATTCGTGCTTATTGTCTTGAAATTTGGGAAGTAATG 654

414 TTGCAATTTCATACTAAAGAAGAAATTCGTGCTTATTGTCTTGAAATTTGGGAAGTGATG 654

*********** ******** ****** **************************** ***

CG8486 CAAGAAGTGTATTATAATGGTACGCATCCAAATGAAGATTATTTGCCCGGAAAACTTCAT 714

**ATCC 33251** CAAGAAGTGTATTATAATGGTACGCATCCAAATGAAGATTATTTGCCCGGAAAACTTCAT 720

CF93-6 CAAGAAGTGTATTATAATGGTACGCACCCGAATGAAGATTATTTGCCTGGAAAACTTCAT 714

84-25 CAAGAAGTGTATTATAATGGTACGCACCCGAATGAAGATTATTTGCCTGGAAAACTTCAT 714

NCTC 11168 CAAGAAGTGTATTATAATGGTACGCACCCGAATGAAGATTATTTGCCTGGAAAACTTCAT 714

IA3902 CAAGAAGTGTATTATAATGGTACGCACCCGAATGAAGATTATTTGCCTGGAAAACTTCAT 714

DFVF1099 CAAGAAGTGTATTATAATGGTACGCACCCGAATGAAGATTATTTGCCTGGAAAACTTCAT 714

305 CAAGAAGTGTATTATAATGGTACGCACCCGAATGAAGATTATTTGCCTGGAAAACTTCAT 714

RM1221 CAAGAAGTGTATTATAATGGCACTCATCCAAATGAAGATTATTTGCCTGGAAAACTTCAT 714

S3 CAAGAAGTGTATTATAATGGCACTCATCCAAATGAAGATTATTTGCCTGGAAAACTTCAT 714

260.94 CAAGAAGTGTATTATAATGGTACGCATCCAAATGAAGATTATTTGCCTGGAAAACTTCAT 714

ICDCCJ07001 CAAGAAGTGTATTATAATGGTACGCATCCAAATGAAGATTATTTGCCTGGAAAACTTCAT 714

HB93-13 CAAGAAGTGTATTATAATGGTACGCATCCAAATGAAGATTATTTGCCTGGAAAACTTCAT 714

81-176 CAAGAAGTGTATTATAATGGTACGCATCCAAATGAAGATTATTTGCCTGGAAAACTTCAT 714

81116 CAAGAAGTGTATTATAATGGTACGCATCCAAATGAAGATTATTTGCCCGGAAAACTTCAT 714

M1 CAAGAAGTGTATTATAGTGGTACGCATCCAAATGAAGATTATTTGCCCGGAAAACTTCAT 714

327 CAAGAAGTGTATTATAATGGTACGCATCCAAATGAAGATTATTTGCCCGGAAAACTTCAT 714

1336 CAAGAAGTGTATTATAATGGTACGCATCCAAATGAAGATTATTTGCCTGGAAAACTTCAT 714

CG8421 CAAGAAGTGTATTATAATGGCACTCATCCAAATGAAGATTATTTGCCTGGAAAACTTCAT 714

414 CAAGAAGTGTATTATAATGGTACACATCCAAATGAAGATTATTTACCTGGAAAGCTTCAT 714

**************** *** ** ** ** ************** ** ***** ******

CG8486 TTAAAACGTAGAGCTAAAGGGCTTAAAGAAAGAGTGGCGATGACAGCTGATCCTATGGGC 774

**ATCC 33251** TTAAAACGTAGAGCTAAAGGGCTTAAAGAAAGAGTGGCGATGACAGCTGATCCTATGGGC 780

CF93-6 TTAAAGCGTAGAGCTAAAGGACTTAAAGAAAGAGTAGCGATGACAGCTGATCCTATGGGT 774

84-25 TTAAAGCGTAGAGCTAAAGGACTTAAAGAAAGAGTAGCGATGACAGCTGATCCTATGGGT 774

NCTC 11168 TTAAAGCGTAGAGCTAAAGGACTTAAAGAAAGAGTAGCGATGACAGCTGATCCTATGGGT 774

IA3902 TTAAAGCGTAGAGCTAAAGGACTTAAAGAAAGAGTAGCGATGACAGCTGATCCTATGGGT 774

DFVF1099 TTAAAGCGTAGAGCTAAAGGACTTAAAGAAAGAGTAGCGATGACAGCTGATCCTATGGGT 774

305 TTAAAGCGTAGAGCTAAAGGACTTAAAGAAAGAGTAGCGATGACAGCTGATCCTATGGGT 774

RM1221 TTAAAGCGTAGAGCTAAAGGACTTAAAGAAAGAGTGGCGATGACAGCTGATCCTATGGGC 774

S3 TTAAAGCGTAGAGCTAAAGGACTTAAAGAAAGAGTGGCGATGACAGCTGATCCTATGGGC 774

260.94 TTAAAGCGTAGAGCCAAAGGACTTAAAGAAAGAGTGGCGATGACAGCTGATCCTATGGGC 774

ICDCCJ07001 TTAAAGCGTAGAGCCAAAGGACTTAAAGAAAGAGTGGCGATGACAGCTGATCCTATGGGC 774

HB93-13 TTAAAGCGTAGAGCCAAAGGACTTAAAGAAAGAGTGGCGATGACAGCTGATCCTATGGGC 774

81-176 TTAAAGCGTAGAGCCAAAGGACTTAAAGAAAGAGTGGCGATGACAGCTGATCCTATGGGC 774

81116 TTAAAGCGTAGAGCCAAAGGACTTAAAGAAAGAGTGGCGATGACAGCTGATCCTATGGGC 774

M1 TTAAAGCGTAGAGCCAAAGGACTTAAAGAAAGAGTGGCGATGACAGCTGATCCTATGGGC 774

327 TTAAAGCGTAGAGCCAAAGGACTTAAAGAAAGAGTGGCGATGACAGCTGATCCTATGGGC 774

1336 TTAAAGCGTAGAGCCAAAGGACTTAAAGAAAGAGTGGCGATGACAGCTGATCCTATGGGC 774

CG8421 TTAAAGCGTAGAGCTAAAGGACTTAAAGAAAGAGTGGCGATGACAGCTGATCCTATGGGC 774

414 TTAAAGCGCAGAGCCAAAGGGCTTAAGAAAAGAGTAGCAATGACGGCTGATCCTATGGGT 774

***** ** ***** ***** ***** ******* ** ***** **************

CG8486 ATTATAGATTTTATTTCTTTATATGCTATTGCGATTGCTGAAGAAAATGCTAGCGGAGCA 834

**ATCC 33251** ATTATAGATTTTATTTCTTTATATGCTATTGCGATTGCTGAAGAAAATGCTAGCGGAGCA 840

CF93-6 ATTATCGATTTTATTTCTTTATATGCTATTGCGATTGCTGAAGAAAATGCTAGCGGAGCA 834

84-25 ATTATCGATTTTATTTCTTTATATGCTATTGCGATTGCTGAAGAAAATGCTAGCGGAGCA 834

NCTC 11168 ATTATCGATTTTATTTCTTTATATGCTATTGCGATTGCTGAAGAAAATGCTAGCGGAGCA 834

IA3902 ATTATCGATTTTATTTCTTTATATGCTATTGCGATTGCTGAAGAAAATGCTAGCGGAGCA 834

DFVF1099 ATTATCGATTTTATTTCTTTATATGCTATTGCGATTGCTGAAGAAAATGCTAGCGGAGCA 834

305 ATTATCGATTTTATTTCTTTATATGCTATTGCGATTGCTGAAGAAAATGCTAGCGGAGCA 834

RM1221 ATTATCGATTTTATTTCTTTATATGCTATTGCGATTGCTGAAGAAAATGCTAGCGGAGCA 834

S3 ATTATCGATTTTATTTCTTTATATGCTATTGCGATTGCTGAAGAAAATGCTAGCGGAGCA 834

260.94 ATTATAGATTTTATTTCTTTATATGCTATTGCGATTGCTGAAGAAAATGCTAGCGGAGCA 834

ICDCCJ07001 ATTATAGATTTTATTTCTTTATATGCTATTGCGATTGCTGAAGAAAATGCTAGCGGAGCA 834

HB93-13 ATTATAGATTTTATTTCTTTATATGCTATTGCGATTGCTGAAGAAAATGCTAGCGGAGCA 834

81-176 ATTATAGATTTTATTTCTTTATATGCTATTGCGATTGCTGAAGAAAATGCTAGCGGAGCA 834

81116 ATTATAGATTTTATTTCTTTATATGCTATTGCGATTGCTGAAGAAAATGCTAGCGGAGCA 834

M1 ATTATAGATTTTATTTCTTTATATGCTATTGCGATTGCTGAAGAAAATGCTAGCGGAGCA 834

327 ATTATAGATTTTATTTCTTTATATGCTATTGCGATTGCTGAAGAAAATGCTAGCGGAGCA 834

1336 ATTATAGATTTTATTTCTTTATATGCTATTGCGATTGCTGAAGAAAATGCTAGCGGAGCA 834

CG8421 ATTATCGATTTTATTTCTTTATATGCTATTGCGATTGCTGAAGAAAATGCTAGCGGAGCA 834

414 ATTATCGATTTTATTTCTTTATACGCTATTGCGATTGCTGAAGAAAATGCTAGTGGAGCA 834

***** ***************** ***************************** ******

CG8486 AAAGTTGTAACCGCGCCAACAAATGGAGCATGTGCCGTTATCCCTGCTGTTATGCTTTAT 894

**ATCC 33251** AAAGTTGTAACCGCGCCAACAAATGGAGCATGTGCCGTTATCCCTGCTGTTATGCTTTAT 900

CF93-6 AAAGTCGTAACCGCACCAACAAATGGAGCATGTGCTGTTATCCCTGCTGTTATGCTTTAT 894

84-25 AAAGTCGTAACCGCACCAACAAATGGAGCATGTGCTGTTATCCCTGCTGTTATGCTTTAT 894

NCTC 11168 AAAGTCGTAACCGCACCAACAAATGGAGCATGTGCTGTTATCCCTGCTGTTATGCTTTAT 894

IA3902 AAAGTCGTAACCGCACCAACAAATGGAGCATGTGCTGTTATCCCTGCTGTTATGCTTTAT 894

DFVF1099 AAAGTCGTAACCGCACCAACAAATGGAGCATGTGCTGTTATCCCTGCTGTTATGCTTTAT 894

305 AAAGTCGTAACCGCACCAACAAATGGAGCATGTGCTGTTATCCCTGCTGTTATGCTTTAT 894

RM1221 AAAGTTGTAACCGCACCAACAAATGGAGCATGTGCTGTTATCCCTGCTGTTATGCTTTAT 894

S3 AAAGTTGTAACCGCACCAACAAATGGAGCATGTGCTGTTATCCCTGCTGTTATGCTTTAT 894

260.94 AAAGTTGTAACCGCGCCAACAAATGGAGCATGTGCTGTTATCCCTGCTGTTATGCTTTAT 894

ICDCCJ07001 AAAGTTGTAACCGCGCCAACAAATGGAGCATGTGCTGTTATCCCTGCTGTTATGCTTTAT 894

HB93-13 AAAGTTGTAACCGCGCCAACAAATGGAGCATGTGCTGTTATCCCTGCTGTTATGCTTTAT 894

81-176 AAAGTTGTAACCGCGCCAACAAATGGAGCATGTGCTGTTATCCCTGCTGTTATGCTTTAT 894

81116 AAAGTTGTAACCGCGCCAACAAATGGAGCATGTGCTGTTATCCCTGCTGTTATGCTTTAT 894

M1 AAAGTTGTAACCGCGCCAACAAATGGAGCATGTGCTGTTATCCCTGCTGTTATGCTTTAT 894

327 AAAGTTGTAACCGCGCCAACAAATGGAGCATGTGCTGTTATCCCTGCTGTTATGCTTTAT 894

1336 AAAGTTGTAACCGCGCCAACAAATGGAGCATGTGCTGTTATCCCTGCTGTTATGCTTTAT 894

CG8421 AAAGTTGTAACCGCACCAACAAATGGAGCATGTGCTGTTATCCCTGCTGTTATGCTTTAT 894

414 AAAGTTGTAACCGCACCAACAAATGGAGCATGTGCTGTTATCCCTGCTGTTATGCTTTAT 894

***** ******** ******************** ************************

CG8486 CTTAAAAATCATACTATAGGTTTTAGTGATGAAAAAGCTATAGAGTTTTTATTAACAGCA 954

**ATCC 33251** CTTAAAAATCATACTATAGGTTTTAGTGATGAAAAAGCTATAGAGTTTTTATTAACAGCA 960

CF93-6 CTTAAAAATCATACTATAGGTTTTAGTGATGAAAAGGTTATAGAGTTTTTATTAACAGCA 954

84-25 CTTAAAAATCATACTATAGGTTTTAGTGATGAAAAGGTTATAGAGTTTTTATTAACAGCA 954

NCTC 11168 CTTAAAAATCATACTATAGGTTTTAGTGATGAAAAGGTTATAGAGTTTTTATTAACAGCA 954

IA3902 CTTAAAAATCATACTATAGGTTTTAGTGATGAAAAGGTTATAGAGTTTTTATTAACAGCA 954

DFVF1099 CTTAAAAATCATACTATAGGTTTTAGTGATGAAAAGGTTATAGAGTTTTTATTAACAGCA 954

305 CTTAAAAATCATACTATAGGTTTTAGTGATGAAAAGGTTATAGAGTTTTTATTAACAGCA 954

RM1221 CTTAAAAACCATACTATAGGTTTTAGTGATGAAAAGGCTATAGAGTTTTTATTAACAGCA 954

S3 CTTAAAAACCATACTATAGGTTTTAGTGATGAAAAGGCTATAGAGTTTTTATTAACAGCA 954

260.94 CTTAAAAATCATACTATAGGTTTTAGCGATGAAAAGGCTATAGAGTTTTTATTAACAGCA 954

ICDCCJ07001 CTTAAAAATCATACTATAGGTTTTAGCGATGAAAAGGCTATAGAGTTTTTATTAACAGCA 954

HB93-13 CTTAAAAATCATACTATAGGTTTTAGCGATGAAAAGGCTATAGAGTTTTTATTAACAGCA 954

81-176 CTTAAAAATCATACTATAGGTTTTAGCGATGAAAAGGCTATAGAGTTTTTATTAACAGCA 954

81116 CTTAAAAATCATACTATAGGTTTTAGTGATGAAAAGGCTATAGAGTTTTTATTAACAGCA 954

M1 CTTAAAAATCATACTATAGGTTTTAGTGATGAAAAGGCTATAGAGTTTTTATTAACAGCA 954

327 CTTAAAAATCATACTATAGGTTTTAGTGATGAAAAGGCTATAGAGTTTTTATTAACAGCA 954

1336 CTTAAAAATCATACTATAGGTTTTAGCGATGAAAAGGCTATAGAGTTTTTATTAACAGCA 954

CG8421 CTTAAAAACCATACTATAGGTTTTAGTGATGAAAAGGCTATAGAGTTTTTATTAACAGCA 954

414 CTTAAAAATCATACTATAGGTTTTAGTGATGAAAAGGCTATAGAGTTTTTATTAACAGCA 954

******** ***************** ******** * **********************

CG8486 ATGCTTATAGGTTCTTTTTATAAGAAAAATGCAAGTATTAGCGGAGCAGAAGCAGGCTGT 1014

**ATCC 33251** ATGCTTATAGGTTCTTTTTATAAGAAAAATGCAAGTATTAGCGGAGCAGAAGCAGGCTGT 1020

CF93-6 ATGCTTATAGGTTCTTTTTATAAGAAAAATGCAAGTATTAGCGGAGCAGAAGCAGGTTGT 1014

84-25 ATGCTTATAGGTTCTTTTTATAAGAAAAATGCAAGTATTAGCGGAGCAGAAGCAGGTTGT 1014

NCTC 11168 ATGCTTATAGGTTCTTTTTATAAGAAAAATGCAAGTATTAGCGGAGCAGAAGCAGGTTGT 1014

IA3902 ATGCTTATAGGTTCTTTTTATAAGAAAAATGCAAGTATTAGCGGAGCAGAAGCAGGTTGT 1014

DFVF1099 ATGCTTATAGGTTCTTTTTATAAGAAAAATGCAAGTATTAGCGGAGCAGAAGCAGGTTGT 1014

305 ATGCTTATAGGTTCTTTTTATAAGAAAAATGCAAGTATTAGCGGAGCAGAAGCAGGTTGT 1014

RM1221 ATGCTTATAGGTTCTTTTTATAAGAAAAATGCAAGCATTAGCGGAGCAGAAGCAGGCTGT 1014

S3 ATGCTTATAGGTTCTTTTTATAAGAAAAATGCAAGCATTAGCGGAGCAGAAGCAGGCTGT 1014

260.94 ATGCTTATAGGTTCTTTTTATAAGAAAAATGCAAGTATTAGCGGAGCAGAAGCAGGTTGT 1014

ICDCCJ07001 ATGCTTATAGGTTCTTTTTATAAGAAAAATGCAAGTATTAGCGGAGCAGAAGCAGGTTGT 1014

HB93-13 ATGCTTATAGGTTCTTTTTATAAGAAAAATGCAAGTATTAGCGGAGCAGAAGCAGGTTGT 1014

81-176 ATGCTTATAGGTTCTTTTTATAAGAAAAATGCAAGTATTAGCGGAGCAGAAGCAGGTTGT 1014

81116 ATGCTTATAGGTTCTTTTTATAAGAAAAATGCAAGTATTAGCGGAGCAGAAGCAGGTTGT 1014

M1 ATGCTTATAGGTTCTTTTTATAAGAAAAATGCAAGTATTAGCGGAGCAGAAGCAGGTTGT 1014

327 ATGCTTATAGGTTCTTTTTATAAGAAAAATGCAAGTATTAGCGGAGCAGAAGCAGGTTGT 1014

1336 ATGCTTATAGGTTCTTTTTATAAGAAAAATGCAAGTATTAGCGGAGCAGAAGCAGGCTGT 1014

CG8421 ATGCTTATAGGTTCTTTTTATAAGAAAAATGCAAGCATTAGCGGAGCAGAAGCAGGCTGT 1014

414 ATGCTTATAGGTTCTTTTTATAAGAAAAATGCAAGTATTAGCGGAGCAGAAGCAGGTTGT 1014

*********************************** ******************** ***

CG8486 CAAGCAGAAATTGGTAGTGCTAGTTCTATGGCAGCTGCGGCTATGGCAACAGTTTTAGGG 1074

**ATCC 33251** CAAGCAGAAATTGGTAGTGCTAGTTCTATGGCAGCTGCAGCTATGGCAACAGTTTTAGGG 1080

CF93-6 CAAGCAGAAATTGGTAGTGCTAGTTCTATGGCAGCTGCGGCTATGGCAACAGTTTTAGGA 1074

84-25 CAAGCAGAAATTGGTAGTGCTAGTTCTATGGCAGCTGCGGCTATGGCAACAGTTTTAGGA 1074

NCTC 11168 CAAGCAGAAATTGGTAGTGCTAGTTCTATGGCAGCTGCGGCTATGGCAACAGTTTTAGGA 1074

IA3902 CAAGCAGAAATTGGTAGTGCTAGTTCTATGGCAGCTGCGGCTATGGCAACAGTTTTAGGA 1074

DFVF1099 CAAGCAGAAATTGGTAGTGCTAGTTCTATGGCAGCTGCGGCTATGGCAACAGTTTTAGGA 1074

305 CAAGCAGAAATTGGTAGTGCTAGTTCTATGGCAGCTGCGGCTATGGCAACAGTTTTAGGA 1074

RM1221 CAAGCAGAAATTGGTAGTGCTAGCTCTATGGCAGCTGCAGCTATGGCAACAGTTTTAGGG 1074

S3 CAAGCAGAAATTGGTAGTGCTAGCTCTATGGCAGCTGCAGCTATGGCAACAGTTTTAGGG 1074

260.94 CAAGCAGAAATTGGTAGTGCTAGTTCGATGGCAGCTGCGGCTATGGCAACAGTTTTAGGG 1074

ICDCCJ07001 CAAGCAGAAATTGGTAGTGCTAGTTCGATGGCAGCTGCGGCTATGGCAACAGTTTTAGGG 1074

HB93-13 CAAGCAGAAATTGGTAGTGCTAGTTCGATGGCAGCTGCGGCTATGGCAACAGTTTTAGGG 1074

81-176 CAAGCAGAAATTGGTAGTGCTAGTTCGATGGCAGCTGCGGCTATGGCAACAGTTTTAGGG 1074

81116 CAAGCAGAAATTGGTAGTGCTAGTTCGATGGCAGCTGCGGCTATGGCAACAGTTTTAGGG 1074

M1 CAAGCAGAAATTGGTAGTGCTAGTTCGATGGCAGCTGCGGCTATGGCAACAGTTTTAGGG 1074

327 CAAGCAGAAATTGGTAGTGCTAGTTCGATGGCAGCTGCGGCTATGGCAACAGTTTTAGGG 1074

1336 CAAGCAGAAATTGGTAGTGCTAGTTCGATGGCAGCTGCGGCTATGGCAACAGTTTTAGGG 1074

CG8421 CAAGCAGAAATTGGTAGTGCTAGCTCTATGGCAGCTGCAGCTATGGCAACAGTTTTAGGG 1074

414 CAAGCAGAAATTGGCAGTGCTAGCTCTATGGCAGCTGCAGCTATGGCAACGGTTTTAGGA 1074

************** ******** ** *********** *********** ********

CG8486 GTTAATGCTTTTAAAGCATGCAACGCTGCTGAAATGGCAATGGAACATCATCTAGGTTTA 1134

**ATCC 33251** GCTAATGCTTTTAAAGCATGCAACGCTGCTGAAATGGCAATGGAACATCATCTAGGTTTA 1140

CF93-6 GCTAATGCTTTTAAAGCATGTAATGCTGCTGAAATGGCAATGGAACATCATCTAGGTCTA 1134

84-25 GCTAATGCTTTTAAAGCATGTAATGCTGCTGAAATGGCAATGGAACATCATCTAGGTCTA 1134

NCTC 11168 GCTAATGCTTTTAAAGCATGTAATGCTGCTGAAATGGCAATGGAACATCATCTAGGTCTA 1134

IA3902 GCTAATGCTTTTAAAGCATGTAATGCTGCTGAAATGGCAATGGAACATCATCTAGGTCTA 1134

DFVF1099 GCTAATGCTTTTAAAGCATGTAATGCTGCTGAAATGGCAATGGAACATCATCTAGGTCTA 1134

305 GCTAATGCTTTTAAAGCATGTAATGCTGCTGAAATGGCAATGGAACATCATCTAGGTCTA 1134

RM1221 GCTAATGCTTTTAAAGCATGCAACGCTGCTGAAATGGCAATGGAACATCATCTAGGTTTA 1134

S3 GCTAATGCTTTTAAAGCATGCAACGCTGCTGAAATGGCAATGGAGCATCATCTAGGTTTA 1134

260.94 GCTAATGCTTTTAAAGCATGCAACGCTGCTGAAATGGCAATGGAACATCATCTAGGTTTA 1134

ICDCCJ07001 GCTAATGCTTTTAAAGCATGCAACGCTGCTGAAATGGCAATGGAACATCATCTAGGTTTA 1134

HB93-13 GCTAATGCTTTTAAAGCATGCAACGCTGCTGAAATGGCAATGGAACATCATCTAGGTTTA 1134

81-176 GCTAATGCTTTTAAAGCATGCAACGCTGCTGAAATGGCAATGGAACATCATCTAGGTTTA 1134

81116 GCTAATGCTTTTAAAGCATGCAACGCTGCTGAAATGGCAATGGAACATCATCTAGGTTTA 1134

M1 GCTAATGCTTTTAAAGCATGCAACGCTGCTGAAATGGCAATGGAACATCATCTAGGTTTA 1134

327 GCTAATGCTTTTAAAGCATGCAACGCTGCTGAAATGGCAATGGAACATCATCTAGGTTTA 1134

1336 GCTAATGCTTTTAAAGCATGCAACGCTGCTGAAATGGCAATGGAACATCATCTAGGTTTA 1134

CG8421 GCTAATGCTTTTAAAGCATGCAACGCTGCTGAAATGGCAATGGAACATCATCTAGGTTTA 1134

414 GCTAATGCTTTTAAAGCGTGTAATGCTGCTGAAATGGCAATGGAGCATCATCTAGGCTTA 1134

* *************** ** ** ******************** *********** **

CG8486 ACTTGCGATCCAGTTGCAGGACTTGTGCAAATTCCTTGCATAGAAAGAAATGCCTTTGGA 1194

**ATCC 33251** ACTTGCGATCCAGTTGCAGGGCTTGTGCAAATTCCTTGCATAGAAAGAAATGCCTTTGGA 1200

CF93-6 ACTTGCGATCCAGTTGCAGGACTTGTGCAAATTCCTTGCATAGAAAGAAATGCCTTTGGA 1194

84-25 ACTTGCGATCCAGTTGCAGGACTTGTGCAAATTCCTTGCATAGAAAGAAATGCCTTTGGA 1194

NCTC 11168 ACTTGCGATCCAGTTGCAGGACTTGTGCAAATTCCTTGCATAGAAAGAAATGCCTTTGGA 1194

IA3902 ACTTGCGATCCAGTTGCAGGACTTGTGCAAATTCCTTGCATAGAAAGAAATGCCTTTGGA 1194

DFVF1099 ACTTGCGATCCAGTTGCAGGACTTGTGCAAATTCCTTGCATAGAAAGAAATGCCTTTGGA 1194

305 ACTTGCGATCCAGTTGCAGGACTTGTGCAAATTCCTTGCATAGAAAGAAATGCCTTTGGA 1194

RM1221 ACTTGCGATCCAGTTGCAGGGCTTGTGCAAATTCCTTGCATAGAAAGAAATGCCTTTGGA 1194

S3 ACTTGCGATCCAGTTGCAGGGCTTGTGCAAATTCCTTGCATAGAAAGAAATGCCTTTGGA 1194

260.94 ACTTGCGATCCAGTTGCAGGACTTGTGCAAATTCCTTGCATAGAAAGAAATGCCTTTGGA 1194

ICDCCJ07001 ACTTGCGATCCAGTTGCAGGACTTGTGCAAATTCCTTGCATAGAAAGAAATGCCTTTGGA 1194

HB93-13 ACTTGCGATCCAGTTGCAGGACTTGTGCAAATTCCTTGCATAGAAAGAAATGCCTTTGGA 1194

81-176 ACTTGCGATCCAGTTGCAGGACTTGTGCAAATTCCTTGCATAGAAAGAAATGCCTTTGGA 1194

81116 ACTTGCGATCCAGTTGCAGGACTTGTGCAAATTCCTTGCATAGAAAGAAATGCCTTTGGA 1194

M1 ACTTGCGATCCAGTTGCAGGACTTGTGCAAATTCCTTGCATAGAAAGAAATGCCTTTGGA 1194

327 ACTTGCGATCCAGTTGCAGGACTTGTGCAAATTCCTTGCATAGAAAGAAATGCCTTTGGA 1194

1336 ACTTGCGATCCAGTTGCAGGGCTTGTGCAAATTCCTTGCATAGAAAGAAATGCCTTTGGA 1194

CG8421 ACTTGCGATCCAGTTGCAGGGCTTGTGCAAATTCCTTGCATAGAAAGAAATGCCTTTGGA 1194

414 ACTTGTGATCCAGTTGCTGGACTTGTGCAAATTCCTTGCATAGAAAGAAATGCCTTTGGA 1194

***** *********** ** ***************************************

CG8486 GCTATTAAGGCTATAAGTGCTGCTAGAATGGCAATGACACGCAAATCCACTCCTATGGTA 1254

**ATCC 33251** GCTATTAAGGCTATAAGTGCTGCTAGAATGGCAATGACACGTAAATCTACTCCTATGGTA 1260

CF93-6 GCCATTAAGGCCATAAGTGCTGCTAGAATGGCAATGACACGCAAATCCACTCCTATGGTA 1254

84-25 GCCATTAAGGCCATAAGTGCTGCTAGAATGGCAATGACACGCAAATCCACTCCTATGGTA 1254

NCTC 11168 GCCATTAAGGCCATAAGTGCTGCTAGAATGGCAATGACACGCAAATCCACTCCTATGGTA 1254

IA3902 GCCATTAAGGCCATAAGTGCTGCTAGAATGGCAATGACACGCAAATCCACTCCTATGGTA 1254

DFVF1099 GCCATTAAGGCCATAAGTGCTGCTAGAATGGCAATGACACGCAAATCCACTCCTATGGTA 1254

305 GCCATTAAGGCCATAAGTGCTGCTAGAATGGCAATGACACGCAAATCCACTCCTATGGTA 1254

RM1221 GCTATTAAGGCTATAAGTGCTGCTAGAATGGCAATGACACGTAAATCTACTCCTATGGTA 1254

S3 GCTATTAAGGCTATAAGTGCTGCTAGAATGGCAATGACACGTAAATCTACTCCTATGGTA 1254

260.94 GCTATTAAGGCTATAAGTGCTGCTAGAATGGCAATGACACGTAAATCTACTCCTATGGTA 1254

ICDCCJ07001 GCTATTAAGGCTATAAGTGCTGCTAGAATGGCAATGACACGTAAATCTACTCCTATGGTA 1254

HB93-13 GCTATTAAGGCTATAAGTGCTGCTAGAATGGCAATGACACGTAAATCTACTCCTATGGTA 1254

81-176 GCTATTAAGGCTATAAGTGCTGCTAGAATGGCAATGACACGTAAATCTACTCCTATGGTA 1254

81116 GCTATTAAGGCTATAAGTGCTGCTAGAATGGCAATGACACGCAAATCTACTCCTATGGTA 1254

M1 GCTATTAAGGCTATAAGTGCTGCTAGAATGGCAATGACACGCAAATCTACTCCTATGGTA 1254

327 GCTATTAAGGCTATAAGTGCTGCTAGAATGGCAATGACACGCAAATCTACTCCTATGGTA 1254

1336 GCTATTAAGGCTATAAGTGCTGCTAGAATGGCAATGACACGTAAATCTACTCCTATGGTA 1254

CG8421 GCTATTAAGGCTATAAGTGCTGCTAGAATGGCAATGACACGCAAATCTACTCCTATGGTA 1254

414 GCTATTAAGGCTATAAGTGCTGCTAGAATGACAATGACGCGTAAATCTACTCCTATGGTA 1254

** ******** ****************** ******* ** ***** ************

CG8486 AGCCTTGATGAGGTTATAGAAACCATGTATGAAACAGGAAAAGATATGAATTATAAATAC 1314

**ATCC 33251** AGCCTTGATGAGGTTATAGAAACCATGTGTGAAACAGGAAAAGATATGAATTATAAATAC 1320

CF93-6 AGCCTTGATGAGGTTATAGAAACCATGTATGAAACAGGAAAAGATATGAATTATAAATAT 1314

84-25 AGCCTTGATGAGGTTATAGAAACCATGTATGAAACAGGAAAAGATATGAATTATAAATAT 1314

NCTC 11168 AGCCTTGATGAGGTTATAGAAACCATGTATGAAACAGGAAAAGATATGAATTATAAATAT 1314

IA3902 AGCCTTGATGAGGTTATAGAAACCATGTATGAAACAGGAAAAGATATGAATTATAAATAT 1314

DFVF1099 AGCCTTGATGAGGTTATAGAAACCATGTATGAAACAGGAAAAGATATGAATTATAAATAT 1314

305 AGCCTTGATGAGGTTATAGAAACCATGTATGAAACAGGAAAAGATATGAATTATAAATAT 1314

RM1221 AGCCTTGATGAGGTTATAGAAACCATGTATGAAACAGGAAAAGATATGAATTATAAATAC 1314

S3 AGCCTTGATGAGGTTATAGAAACCATGTATGAAACAGGAAAAGATATGAATTATAAATAC 1314

260.94 AGCCTTGATGAGGTTATAGAAACCATGTATGAAACAGGAAAAGATATGAATTATAAATAC 1314

ICDCCJ07001 AGCCTTGATGAGGTTATAGAAACCATGTATGAAACAGGAAAAGATATGAATTATAAATAC 1314

HB93-13 AGCCTTGATGAGGTTATAGAAACCATGTATGAAACAGGAAAAGATATGAATTATAAATAC 1314

81-176 AGCCTTGATGAGGTTATAGAAACCATGTATGAAACAGGAAAAGATATGAATTATAAATAC 1314

81116 AGCCTTGATGAGGTTATAGAAACCATGTATGAAACAGGAAAAGATATGAATTATAAATAC 1314

M1 AGCCTTGATGAGGTTATAGAAACCATGTATGAAACAGGAAAAGATATGAATTATAAATAC 1314

327 AGCCTTGATGAGGTTATAGAAACCATGTATGAAACAGGAAAAGATATGAATTATAAATAC 1314

1336 AGCCTTGATGAGGTTATAGAAACCATGTATGAAACAGGAAAAGATATGAATTATAAATAC 1314

CG8421 AGCCTTGATGAGGTTATAGAAACCATGTATGAAACAGGAAAAGATATGAATTATAAATAC 1314

414 AGCCTTGATGAGGTTATAGAAACCATGTATGAAACAGGAAAAGATATGAATTATAAATAT 1314

**************************** ******************************

CG8486 AAAGAAACTTCTTTAGGTGGTCTTGCTACAAATTTAAAAACAGTATGCTAA 1365

**ATCC 33251** AAAGAAACTTCTTTAGGTGGTCTTGCTACAAATTTAAAAACAGTATGCTAA 1371

CF93-6 AAAGAAACTTCTTTAGGTGGTCTTGCTACAAATTTAAAAACAGTATGCTAA 1365

84-25 AAAGAAACTTCTTTAGGTGGTCTTGCTACAAATTTAAAAACAGTATGCTAA 1365

NCTC 11168 AAAGAAACTTCTTTAGGTGGTCTTGCTACAAATTTAAAAACAGTATGCTAA 1365

IA3902 AAAGAAACTTCTTTAGGTGGTCTTGCTACAAATTTAAAAACAGTATGCTAA 1365

DFVF1099 AAAGAAACTTCTTTAGGTGGTCTTGCTACAAATTTAAAAACAGTATGCTAA 1365

305 AAAGAAACTTCTTTAGGTGGTCTTGCTACAAATTTAAAAACAGTATGCTAA 1365

RM1221 AAAGAAACTTCTTTAGGTGGTCTTGCTACAAATTTAAAAACAGTATGCTAA 1365

S3 AAAGAAACTTCTTTAGGTGGTCTTGCTACAAATTTAAAAACAGTATGCTAA 1365

260.94 AAAGAAACTTCTTTAGGTGGTCTTGCTACAAATTTAAAAACAGTATGCTAA 1365

ICDCCJ07001 AAAGAAACTTCTTTAGGTGGTCTTGCTACAAATTTAAAAACAGTATGCTAA 1365

HB93-13 AAAGAAACTTCTTTAGGTGGTCTTGCTACAAATTTAAAAACAGTATGCTAA 1365

81-176 AAAGAAACTTCTTTAGGTGGTCTTGCTACAAATTTAAAAACAGTATGCTAA 1365

81116 AAAGAAACTTCTTTAGGTGGTCTTGCTACAAATTTAAAAACAGTATGCTAA 1365

M1 AAAGAAACTTCTTTAGGTGGTCTTGCTACAAATTTAAAAACAGTATGCTAA 1365

327 AAAGAAACTTCTTTAGGTGGTCTTGCTACAAATTTAAAAACAGTATGCTAA 1365

1336 AAAGAAACTTCTTTAGGTGGTCTTGCTACAAATTTAAAAACAGTATGCTAA 1365

CG8421 AAAGAAACTTCTTTAGGTGGTCTTGCTACAAATTTAAAAACAGTATGCTAA 1365

414 AAAGAAACTTCTTTAGGCGGTCTTGCTACAAATTTAAAAACAGTATGCTAA 1365

***************** *********************************

**Figure S1. Nucleotide sequence comparison of the L-serine dehydratase *sdaA* genes in indifferent *C. jejuni* isolates.**
