# Supplementary material for: Contribution of Amino Acid Catabolism to the Tissue Specific Persistence of Campylobacter jejuni in a Murine Colonization Model
Source: PLoS One. 2012 Nov 30;7(11):e50699. doi: 10.1371/journal.pone.0050699 (PMC3511319; doi:10.1371/journal.pone.0050699)
Supplement: Figure S2 — Nucleotide sequence comparison of the serine transporter sdaC genes in different C. jejuni isolates. The published sequences of the sdaC genes from several C. jejuni isolates were compared using ClustalW (www.ebi.ac.uk/Tools/msa/clustalW2). The sources for the nucleotide sequences were obtained from GenBank (NCBI) and have following accession numbers: 260.94 (CJJ26094_1676; NZ_AANK01000006), ICDCCJ07001 (ICDCCJ07001_1540; NC_014802), 81116 (C8J_1527; NC_009839), M1 (CJM1_1566; CP001900), 327(CSU_0678; ADHM01000033.1), DFVF1099 (CSQ_0900; ADHK01000020), 305 (CSS_1724; ADHL01000259.1), IA3902 (CJSA_1537; CP001876), NCTC11168 (Cj1625c; NC_002163), CG8486 (Cj8486_1667c; NZ_AASY01000001), 84-25 (CJJ8425_1709; NZ_AANT02000001), CF93-6 (CJJCF936_1719; NZ_AANJ01000002), CG8421 (Cj8421_1679; NZ_ABGQ01000002), ATCC 33251 (this study), RM1221 (CJE1797; NC_003912), S3 (CJS3_1706; CP001960), HB93-13 (CJJHB9313_1616; NZ_AANQ01000001), 81-176 (CJJ81176_1616; NC_008787), 1336 (C1336_000330074; NZ_ADGL01000024), 414 (C414_000010126; NZ_ADGM01000001). (DOC) [file pone.0050699.s002.doc]

260.94 ATGAATACTCCTAAATGGACTAGTCACGATACAAGGTGGGTTTTATCTCTTTTTGGAACA 60

ICDCCJ07001 ATGAATACTCCTAAATGGACTAGTCACGATACAAGGTGGGTTTTATCTCTTTTTGGAACA 60

81116 ATGAATACTCCTAAATGGACTAGTCACGATACAAGGTGGGTTTTATCTCTTTTTGGAACA 60

M1 ATGAATACTCCTAAATGGACTAGTCACGATACAAGGTGGGTTTTATCTCTTTTTGGAACA 60

327 ATGAATACTCCTAAATGGACTAGTCACGATACAAGGTGGGTTTTATCTCTTTTTGGAACA 60

DFVF1099 ATGAATACTCCTAAATGGACTAGTCACGATACAAGGTGGGTTTTATCTCTTTTTGGAACA 60

305 ATGAATACTCCTAAATGGACTAGTCACGATACAAGGTGGGTTTTATCTCTTTTTGGAACA 60

IA3902 ATGAATACTCCTAAATGGACTAGTCACGATACAAGGTGGGTTTTATCTCTTTTTGGAACA 60

NCTC 11168 ATGAATACTCCTAAATGGACTAGTCACGATACAAGGTGGGTTTTATCTCTTTTTGGAACA 60

CG8486 ATGAATACTCCTAAATGGACTAGTCACGATACAAGGTGGGTTTTATCTCTTTTTGGAACA 60

84-25 ATGAATACTCCTAAATGGACTAGTCACGATACAAGGTGGGTTTTATCTCTTTTTGGAACA 60

CF93-6 ATGAATACTCCTAAATGGACTAGTCACGATACAAGGTGGGTTTTATCTCTTTTTGGAACA 60

CG8421 ATGAATACTCCTAAATGGACTAGTCACGATACAAGGTGGGTTTTATCTCTTTTTGGAACA 60

**ATCC 33251** ATGAATACTCCTAAATGGACTAGTCACGATACAAGGTGGGTTTTATCTCTTTTTGGAACA 60

RM1221 ATGAATACTCCTAAATGGACTAGTCACGATACAAGGTGGGTTTTATCTCTTTTTGGAACA 60

S3 ATGAATACTCCTAAATGGACTAGTCACGATACAAGGTGGGTTTTATCTCTTTTTGGAACA 60

HB93-13 ATGAATACTCCTAAATGGACTAGTCACGATACAAGGTGGGTTTTATCTCTTTTTGGAACA 60

81-176 ATGAATACTCCTAAATGGACTAGTCACGATACAAGGTGGGTTTTATCTCTTTTTGGAACA 60

1336 ATGAATACTCCTAAATGGACTAGTCACGATACAAGGTGGGTTTTATCTCTTTTTGGAACA 60

414 ATGAATACTCCTAAATGGACTAGTCACGATACAAGATGGGTTTTATCTCTTTTTGGAACA 60

*********************************** ************************

260.94 GCTATTGGAGCCGGCGTACTTTTGTTACCTATTAGTGCTGGGCTTGGTGGATTGATACCT 120

ICDCCJ07001 GCTATTGGAGCCGGCGTACTTTTGTTACCTATTAGTGCTGGGCTTGGTGGATTGATACCT 120

81116 GCTATTGGAGCTGGCGTACTTTTGTTACCTATTAGTGCTGGGCTTGGTGGATTGATACCT 120

M1 GCTATTGGAGCTGGCGTACTTTTGTTACCTATTAGTGCTGGGCTTGGTGGATTGATACCT 120

327 GCTATTGGAGCTGGCGTACTTTTGTTACCTATTAGTGCTGGGCTTGGTGGATTGATACCT 120

DFVF1099 GCTATTGGAGCCGGCGTACTTTTGTTGCCTATTAGTGCTGGGCTTGGTGGATTGATACCT 120

305 GCTATTGGAGCCGGCGTACTTTTGTTGCCTATTAGTGCTGGGCTTGGTGGATTGATACCT 120

IA3902 GCTATTGGAGCCGGCGTACTTTTGTTGCCTATTAGTGCTGGGCTTGGTGGATTGATACCT 120

NCTC 11168 GCTATTGGAGCCGGCGTACTTTTGTTGCCTATTAGTGCTGGGCTTGGTGGATTGATACCT 120

CG8486 GCTATTGGAGCCGGCGTACTTTTGTTGCCTATTAGTGCTGGGCTTGGTGGATTGATACCT 120

84-25 GCTATTGGAGCCGGCGTACTTTTGTTGCCTATTAGTGCTGGGCTTGGTGGATTGATACCT 120

CF93-6 GCTATTGGAGCCGGCGTACTTTTGTTGCCTATTAGTGCTGGGCTTGGTGGATTGATACCT 120

CG8421 GCTATTGGAGCCGGCGTACTTTTGTTACCTATTAGTGCTGGGCTTGGTGGATTGATACCT 120

**ATCC 33251** GCTATTGGAGCTGGCGTACTTTTGTTACCTATTAGTGCTGGGCTTGGTGGATTGATACCT 120

RM1221 GCTATTGGAGCCGGCGTACTTTTGTTACCTATTAGTGCTGGGCTTGGTGGATTGATACCT 120

S3 GCTATTGGAGCCGGCGTACTTTTGTTACCTATTAGTGCTGGGCTTGGTGGATTGATACCT 120

HB93-13 GCTATTGGAGCCGGCGTACTTTTGTTACCTATTAGTGCTGGGCTTGGTGGATTGATACCT 120

81-176 GCTATTGGAGCCGGCGTACTTTTGTTACCTATTAGTGCTGGGCTTGGTGGATTGATACCT 120

1336 GCTATTGGAGCCGGTGTACTTTTGTTACCTATTAGTGCTGGGCTTGGTGGATTGATACCT 120

414 GCTATTGGGGCTGGCGTACTTTTGTTACCTATTAGTGCTGGACTTGGTGGATTAATACCT 120

******** ** ** *********** ************** *********** ******

260.94 TTGTTAGTTATACTTGTTTTAGCTTTTCCTATGACTTACTTAGCTCATAGAAATTTATGT 180

ICDCCJ07001 TTGTTAGTTATACTTGTTTTAGCTTTTCCTATGACTTACTTAGCTCATAGAAATTTATGT 180

81116 TTGTTAGTTATACTTGTTTTAGCTTTTCCTATGACTTACTTAGCTCATAGAAATTTATGT 180

M1 TTGTTAGTTATACTTGTTTTAGCTTTTCCTATGACTTACTTAGCTCATAGAAATTTATGT 180

327 TTGTTAGTTATACTTGTTTTAGCTTTTCCTATGACTTACTTAGCTCATAGAAATTTATGT 180

DFVF1099 TTGTTAGTTATACTTGTTTTAGCTTTTCCTATGACTTACTTAGCTCATAGAAATTTATGT 180

305 TTGTTAGTTATACTTGTTTTAGCTTTTCCTATGACTTACTTAGCTCATAGAAATTTATGT 180

IA3902 TTGTTAGTTATACTTGTTTTAGCTTTTCCTATGACTTACTTAGCTCATAGAAATTTATGT 180

NCTC 11168 TTGTTAGTTATACTTGTTTTAGCTTTTCCTATGACTTACTTAGCTCATAGAAATTTATGT 180

CG8486 TTGTTAGTTATACTTGTTTTAGCTTTTCCTATGACTTACTTAGCTCATAGAAATTTATGT 180

84-25 TTGTTAGTTATACTTGTTTTAGCTTTTCCTATGACTTACTTAGCTCATAGAAATTTATGT 180

CF93-6 TTGTTAGTTATACTTGTTTTAGCTTTTCCTATGACTTACTTAGCTCATAGAAATTTATGT 180

CG8421 TTGTTAGTTATACTTGTTTTAGCTTTTCCTATGACTTACTTAGCTCATAGAAATTTATGT 180

**ATCC 33251** TTGTTAGTTATACTTGTTTTAGCTTTTCCTATGACTTACTTAGCTCATAGAAATTTATGT 180

RM1221 TTATTAGTTATACTTGTTTTAGCTTTTCCTATGACTTACTTAGCTCATAGAAATTTATGT 180

S3 TTATTAGTTATACTTGTTTTAGCTTTTCCTATGACTTACTTAGCTCATAGAAATTTATGT 180

HB93-13 TTGTTAGTTATACTTGTTTTAGCTTTTCCTATGACTTACTTAGCTCATAGAAATTTATGT 180

81-176 TTGTTAGTTATACTTGTTTTAGCTTTTCCTATGACTTACTTAGCTCATAGAAATTTATGT 180

1336 TTGTTAGTTATACTTGTTTTAGCTTTTCCTATGACTTACTTAGCTCATAGAAATTTATGT 180

414 TTGCTGATTATACTTGTTTTAGCTTTTCCTATGACCTATTTAGCTCATAGAAATTTGTGT 180

** * **************************** ** ***************** ***

260.94 CGCTTTGTGCTTTCAAGTTCTAATCCAAAAGATGATATTACTTTTGTTGCAGAAAGTTAT 240

ICDCCJ07001 CGCTTTGTGCTTTCAAGTTCTAATCCAAAAGATGATATTACTTTTGTTGCAGAAAGTTAT 240

81116 CGCTTTGTGCTTTCAAGTTCTAATCCAAAAGATGATATTACTTTTGTTGCAGAAAGTTAT 240

M1 CGCTTTGTGCTTTCAAGTTCTAATCCAAAAGATGATATTACTTTTGTTGCAGAAAGTTAT 240

327 CGCTTTGTGCTTTCAAGTTCTAATCCAAAAGATGATATTACTTTTGTTGCAGAAAGTTAT 240

DFVF1099 CGCTTTGTGCTTTCAAGTTCTAATCCAAAAGATGATATTACTTTTGTTGCAGAAAGTTAT 240

305 CGCTTTGTGCTTTCAAGTTCTAATCCAAAAGATGATATTACTTTTGTTGCAGAAAGTTAT 240

IA3902 CGCTTTGTGCTTTCAAGTTCTAATCCAAAAGATGATATTACTTTTGTTGCAGAAAGTTAT 240

NCTC 11168 CGCTTTGTGCTTTCAAGTTCTAATCCAAAAGATGATATTACTTTTGTTGCAGAAAGTTAT 240

CG8486 CGCTTTGTGCTTTCAAGTTCTAATCCAAAAGATGATATTACTTTTGTTGCAGAAAGTTAT 240

84-25 CGCTTTGTGCTTTCAAGTTCTAATCCAAAAGATGATATTACTTTTGTTGCAGAAAGTTAT 240

CF93-6 CGCTTTGTGCTTTCAAGTTCTAATCCAAAAGATGATATTACTTTTGTTGCAGAAAGTTAT 240

CG8421 CGCTTTGTGCTTTCAAGTTCTAATCCAAAAGATGATATTACTTTTGTTGCAGAAAGTTAT 240

**ATCC 33251** CGCTTTGTGCTTTCAAGTTCTAATCCAAAAGATGATATTACTTTTGTTGCAGAAAGTTAT 240

RM1221 CGCTTTGTGCTTTCAAGTTCTAATCCAAAAGATGATATTACTTTTGTTGCAGAAAGTTAT 240

S3 CGCTTTGTGCTTTCAAGTTCTAATCCAAAAGATGATATTACTTTTGTTGCAGAAAGTTAT 240

HB93-13 CGCTTTGTGCTTTCAAGTTCTAATCCAAAAGATGATATTACTTTTGTTGCAGAAAGTTAT 240

81-176 CGCTTTGTGCTTTCAAGTTCTAATCCAAAAGATGATATTACTTTTGTTGCAGAAAGTTAT 240

1336 CGCTTTGTGCTTTCAAGTTCTAATCCAAAAGATGATATTACTTTTGTTGCAGAAAGTTAT 240

414 CGCTTTGTGCTTTCAAGTTCTAATCCAAAAGATGATATTACTTTTGTTGCGGAAAGTTAT 240

************************************************** *********

260.94 TTTGGCAAAGGTGGTGGATTTTTAATTACACTTTTGTATTTTTTTGCTATTTTGCCTATT 300

ICDCCJ07001 TTTGGCAAAGGTGGTGGATTTTTAATTACACTTTTGTATTTTTTTGCTATTTTGCCTATT 300

81116 TTTGGCAAAGGTGGTGGATTTTTAATTACACTTTTGTATTTTTTTGCTATTTTACCTATT 300

M1 TTTGGCAAAGGTGGTGGATTTTTAATTACACTTTTGTATTTTTTTGCTATTTTACCTATT 300

327 TTTGGCAAAGGTGGTGGATTTTTAATTACACTTTTGTATTTTTTTGCTATTTTACCTATT 300

DFVF1099 TTTGGCAAGGGCGGTGGATTTTTAATTACACTTTTGTATTTTTTTGCTATTTTGCCTATT 300

305 TTTGGCAAGGGCGGTGGATTTTTAATTACACTTTTGTATTTTTTTGCTATTTTGCCTATT 300

IA3902 TTTGGCAAGGGCGGTGGATTTTTAATTACACTTTTGTATTTTTTTGCTATTTTGCCTATT 300

NCTC 11168 TTTGGCAAGGGCGGTGGATTTTTAATTACACTTTTGTATTTTTTTGCTATTTTGCCTATT 300

CG8486 TTTGGCAAGGGCGGTGGATTTTTAATTACACTTTTGTATTTTTTTGCTATTTTGCCTATT 300

84-25 TTTGGCAAGGGCGGTGGATTTTTAATTACACTTTTGTATTTTTTTGCTATTTTGCCTATT 300

CF93-6 TTTGGCAAGGGCGGTGGATTTTTAATTACACTTTTGTATTTTTTTGCTATTTTGCCTATT 300

CG8421 TTTGGCAAGGGCGGTGGATTTTTAATTACACTTTTGTATTTTTTTGCTATTTTGCCTATT 300

**ATCC 33251** TTTGGCAAAGGCGGTGGATTTTTAATTACACTTTTGTATTTTTTTGCTATTTTGCCTATT 300

RM1221 TTTGGCAAGGGCGGTGGATTTTTAATTACACTTTTGTATTTTTTTGCTATTTTGCCTATT 300

S3 TTTGGCAAGGGCGGTGGATTTTTAATTACACTTTTGTATTTTTTTGCTATTTTGCCTATT 300

HB93-13 TTTGGCAAGGGCGGTGGATTTTTAATTACACTTTTGTATTTTTTTGCTATTTTACCTATT 300

81-176 TTTGGCAAGGGCGGTGGATTTTTAATTACACTTTTGTATTTTTTTGCTATTTTACCTATT 300

1336 TTTGGCAAGGGCGGTGGATTTTTAATTACACTTTTGTATTTTTTTGCTATTTTACCTATT 300

414 TTTGGCAAAGGTGGTGGATTTTTAATTACACTTTTGTATTTTTTTGCTATTTTGCCTATT 300

******** ** ***************************************** ******

260.94 TTATTGGTTTATAGTGCTAATCTTACAACAACTTTACTTGAGTTCTTAATCAATCAGTTT 360

ICDCCJ07001 TTATTGGTTTATAGTGCTAATCTTACAACAACTTTACTTGAGTTCTTAATCAATCAGTTT 360

81116 TTATTGGTTTATAGTGCTAATCTTACAACAACTTTGCTTGAGTTCTTAATCAATCAGTTT 360

M1 TTATTGGTTTATAGTGCTAATCTTACAACAACTTTGCTTGAGTTCTTAATCAATCAGTTT 360

327 TTATTGGTTTATAGTGCTAATCTTACAACAACTTTGCTTGAGTTCTTAATCAATCAGTTT 360

DFVF1099 TTATTGGTTTATAGTGCTAATCTTACAACAACTTTGCTTGAGTTCTTAATCAATCAGTTC 360

305 TTATTGGTTTATAGTGCTAATCTTACAACAACTTTGCTTGAGTTCTTAATCAATCAGTTC 360

IA3902 TTATTGGTTTATAGTGCTAATCTTACAACAACTTTGCTTGAGTTCTTAATCAATCAGTTC 360

NCTC 11168 TTATTGGTTTATAGTGCTAATCTTACAACAACTTTGCTTGAGTTCTTAATCAATCAGTTC 360

CG8486 TTATTGGTTTATAGTGCTAATCTTACAACAACTTTGCTTGAGTTCTTAATCAATCAGTTC 360

84-25 TTATTGGTTTATAGTGCTAATCTTACAACAACTTTGCTTGAGTTCTTAATCAATCAGTTC 360

CF93-6 TTATTGGTTTATAGTGCTAATCTTACAACAACTTTGCTTGAGTTCTTAATCAATCAGTTC 360

CG8421 TTATTGGTTTATAGTGCTAATCTTACAACAACTTTGCTTGAGTTCTTAATCAATCAGTTC 360

**ATCC 33251** TTATTGGTTTATAGTGCTAATCTTACAACAACTTTGCTTGAGTTCTTAATCAATCAGTTT 360

RM1221 TTATTGGTTTATAGTGCTAATCTTACAACAACTTTGCTTGAGTTCTTAATCAATCAGTTC 360

S3 TTATTGGTTTATAGTGCTAATCTTACAACAACTTTGCTTGAGTTCTTAATCAATCAGTTC 360

HB93-13 TTATTGGTTTATAGTGCTAATCTTACAACAACTTTGCTTGAGTTCTTAATCAATCAGTTT 360

81-176 TTATTGGTTTATAGTGCTAATCTTACAACAACTTTGCTTGAGTTCTTAATCAATCAGTTT 360

1336 TTATTGGTTTATAGTGCTAATCTTACAACAACTTTGCTTGAGTTCTTAATCAATCAGTTT 360

414 TTATTAGTTTATAGTGCTAATCTTACAACAACTTTACTTGAGTTTTTAATCAATCAATTT 360

***** ***************************** ******** *********** **

260.94 AATTTCAATGCAGATCTTACTCATGCTGCTCGTTGGTGGGTGAGTTTTTTGATTGTTGGA 420

ICDCCJ07001 AATTTCAATGCAGATCTTACTCATGCTGCTCGTTGGTGGGTGAGTTTTTTGATTGTTGGA 420

81116 AATTTCAATGCAGATCTTACTCATGCTGCTCGTTGGTGGGTGAGTTTTTTGATTGTTGGA 420

M1 AATTTCAATGCAGATCTTACTCATGCTGCTCGTTGGTGGGTGAGTTTTTTGATTGTTGGA 420

327 AATTTCAATGCAGATCTTACTCATGCTGCTCGTTGGTGGGTGAGTTTTTTGATTGTTGGA 420

DFVF1099 AATTTCAATGCAGATCTTACTCATGCTGCTCGTTGGTGGGTGAGTTTTTTGATTGTTGGA 420

305 AATTTCAATGCAGATCTTACTCATGCTGCTCGTTGGTGGGTGAGTTTTTTGATTGTTGGA 420

IA3902 AATTTCAATGCAGATCTTACTCATGCTGCTCGTTGGTGGGTGAGTTTTTTGATTGTTGGA 420

NCTC 11168 AATTTCAATGCAGATCTTACTCATGCTGCTCGTTGGTGGGTGAGTTTTTTGATTGTTGGA 420

CG8486 AATTTCAATGCAGATCTTACTCATGCTGCTCGTTGGTGGGTGAGTTTTTTGATTGTTGGA 420

84-25 AATTTCAATGCAGATCTTACTCATGCTGCTCGTTGGTGGGTGAGTTTTTTGATTGTTGGA 420

CF93-6 AATTTCAATGCAGATCTTACTCATGCTGCTCGTTGGTGGGTGAGTTTTTTGATTGTTGGA 420

CG8421 AATTTCAATGCAGATCTTACTCATGCTGCTCGTTGGTGGGTGAGTTTTTTGATTGTTGGA 420

**ATCC 33251** AATTTCAATGCAGATCTTACTCATGCTGCTCGTTGGTGGGTGAGTTTTTTGATTGTTGGA 420

RM1221 AATTTCAATGCAGATCTTACTCATGCTGCTCGTTGGTGGGTGAGTTTTTTGATTGTTGGA 420

S3 AATTTCAATGCAGATCTTACTCATGCTGCTCGTTGGTGGGTGAGTTTTTTGATTGTTGGA 420

HB93-13 AATTTCAATGCAGATCTTACTCATGCTGCTCGTTGGTGGGTGAGTTTTTTGATTGTTGGA 420

81-176 AATTTCAATGCAGATCTTACTCATGCTGCTCGTTGGTGGGTGAGTTTTTTGATTGTTGGA 420

1336 AATTTCAATGCAGATCTTACTCATGCTGCTCGTTGGTGGGTGAGTTTTTTGATTGTTGGA 420

414 AATTTCAATGCAGATCTTATTTATGCTGCTCGTTGGTGGATAGGTTTTTTAATCGTTGGA 420

******************* * ***************** * ******* ** ******

260.94 GTTTTGGTTTTGATTTCTATTCTTGGTGAAAATGTTGTAACAAAAGCAATGAGTTTTCTT 480

ICDCCJ07001 GTTTTGGTTTTGATTTCTATTCTTGGTGAAAATGTTGTAACAAAAGCAATGAGTTTTCTT 480

81116 GTTTTGGTTTTGATTTCTATTCTTGGTGAAAATGTTGTAACAAAAGCAATGAGTTTTCTT 480

M1 GTTTTGGTTTTGATTTCTATTCTTGGTGAAAATGTTGTAACAAAAGCAATGAGTTTTCTT 480

327 GTTTTGGTTTTGATTTCTATTCTTGGTGAAAATGTTGTAACAAAAGCAATGAGTTTTCTT 480

DFVF1099 GTTTTGGTTTTGATTTCTATTCTTGGTGAAAATGTTGTAACAAAAGCAATGAGTTTTCTT 480

305 GTTTTGGTTTTGATTTCTATTCTTGGTGAAAATGTTGTAACAAAAGCAATGAGTTTTCTT 480

IA3902 GTTTTGGTTTTGATTTCTATTCTTGGTGAAAATGTTGTAACAAAAGCAATGAGTTTTCTT 480

NCTC 11168 GTTTTGGTTTTGATTTCTATTCTTGGTGAAAATGTTGTAACAAAAGCAATGAGTTTTCTT 480

CG8486 GTTTTGGTTTTGATTTCTATTCTTGGTGAAAATGTTGTAACAAAAGCAATGAGTTTTCTT 480

84-25 GTTTTGGTTTTGATTTCTATTCTTGGTGAAAATGTTGTAACAAAAGCAATGAGTTTTCTT 480

CF93-6 GTTTTGGTTTTGATTTCTATTCTTGGTGAAAATGTTGTAACAAAAGCAATGAGTTTTCTT 480

CG8421 GTTTTGGTTTTGATTTCTATTCTTGGTGAAAATGTTGTAACAAAAGCAATGAGTTTTCTT 480

**ATCC 33251** GTTTTGGTTTTGATTTCTATTCTTGGTGAAAATGTTGTAACAAAAGCAATGAGTTTTCTT 480

RM1221 GTTTTGGTTTTGATTTCTATTCTTGGTGAAAATGTTGTAACAAAAGCAATGAGTTTTCTT 480

S3 GTTTTGGTTTTGATTTCTATTCTTGGTGAAAATGTTGTAACAAAAGCAATGAGTTTTCTT 480

HB93-13 GTTTTGGTTTTGATTTCTATTCTTGGTGAAAATGTTGTAACAAAAGCAATGAGTTTTCTT 480

81-176 GTTTTGGTTTTGATTTCTATTCTTGGTGAAAATGTTGTAACAAAAGCAATGAGTTTTCTT 480

1336 GTTTTGGTTTTGATTTCTATTCTTGGTGAAAATGTTGTAACAAAAGCAATGAGTTTTCTT 480

414 GTTTTGATTCTTATTTCTATTCTTGGTGAAAATGTTGTAACAAAAGCAATGAGCTTTCTT 480

****** ** * ***************************************** ******

260.94 GTTTTTCCTTTTATTATATTTTTATTTATTTTTTCATTGCTTTTAATTCCACAATGGAAT 540

ICDCCJ07001 GTTTTTCCTTTTATTATATTTTTATTTATTTTTTCATTGCTTTTAATTCCACAATGGAAT 540

81116 GTTTTTCCTTTTATTATATTTTTATTTATTTTTTCATTGCTTTTAATTCCACAATGGAAT 540

M1 GTTTTTCCTTTTATTATATTTTTATTTATTTTTTCATTGCTTTTAATTCCACAATGGAAT 540

327 GTTTTTCCTTTTATTATATTTTTATTTATTTTTTCATTGCTTTTAATTCCACAATGGAAT 540

DFVF1099 GTTTTTCCTTTTATTATATTTTTATTTATTTTTTCATTGCTTTTAATTCCACAATGGAAT 540

305 GTTTTTCCTTTTATTATATTTTTATTTATTTTTTCATTGCTTTTAATTCCACAATGGAAT 540

IA3902 GTTTTTCCTTTTATTATATTTTTATTTATTTTTTCATTGCTTTTAATTCCACAATGGAAT 540

NCTC 11168 GTTTTTCCTTTTATTATATTTTTATTTATTTTTTCATTGCTTTTAATTCCACAATGGAAT 540

CG8486 GTTTTTCCTTTTATTATATTTTTATTTATTTTTTCATTGCTTTTAATTCCACAATGGAAT 540

84-25 GTTTTTCCTTTTATTATATTTTTATTTATTTTTTCATTGCTTTTAATTCCACAATGGAAT 540

CF93-6 GTTTTTCCTTTTATTATATTTTTATTTATTTTTTCATTGCTTTTAATTCCACAATGGAAT 540

CG8421 GTTTTTCCTTTTATTATATTTTTATTTATTTTTTCATTGCTTTTAATTCCACAATGGAAT 540

**ATCC 33251** GTTTTTCCTTTTATTATATTTTTATTTATTTTTTCATTGCTTTTAATTCCACAATGGAAT 540

RM1221 GTTTTTCCTTTTATTATATTTTTATTTATTTTTTCATTGCTTTTAATTCCACAATGGAAT 540

S3 GTTTTTCCTTTTATTATATTTTTATTTATTTTTTCATTGCTTTTAATTCCACAATGGAAT 540

HB93-13 GTTTTTCCTTTTATTATATTTTTATTTATTTTTTCATTGCTTTTAATTCCACAATGGAAT 540

81-176 GTTTTTCCTTTTATTATATTTTTATTTATTTTTTCATTGCTTTTAATTCCACAATGGAAT 540

1336 GTTTTTCCTTTTATTATATTTTTATTTATTTTTTCATTGCTTTTAATTCCACAATGGAAT 540

414 GTTTTTCCTTTTATTATATTTTTATTTATTTTTTCATTACTTTTAATTCCGCAATGGAAC 540

************************************** *********** ********

260.94 TCATCATTATTTACAAATGTTGATTTTTCCGTAATTTCAACAAGTAATTTTTGGGTTACT 600

ICDCCJ07001 TCATCATTATTTACAAATGTTGATTTTTCCGTAATTTCAACAAGTAATTTTTGGGTTACT 600

81116 TCATCATTATTTACAAATGTTGATTTTTCCGTAATTTCAACAAGTAATTTTTGGGTTACT 600

M1 TCATCATTATTTACAAATGTTGATTTTTCCGTAATTTCAACAAGTAATTTTTGGGTTACT 600

327 TCATCATTATTTACAAATGTTGATTTTTCCGTAATTTCAACAAGTAATTTTTGGGTTACT 600

DFVF1099 TTATCGTTATTTGCAAATGTTGATTTTTCCGTAATTTCAACAAGTAATTTTTGGGTTACT 600

305 TTATCGTTATTTGCAAATGTTGATTTTTCCGTAATTTCAACAAGTAATTTTTGGGTTACT 600

IA3902 TTATCGTTATTTGCAAATGTTGATTTTTCCGTAATTTCAACAAGTAATTTTTGGGTTACT 600

NCTC 11168 TTATCGTTATTTGCAAATGTTGATTTTTCCGTAATTTCAACAAGTAATTTTTGGGTTACT 600

CG8486 TTATCGTTATTTGCAAATGTTGATTTTTCCGTAATTTCAACAAGTAATTTTTGGGTTACT 600

84-25 TTATCGTTATTTGCAAATGTTGATTTTTCCGTAATTTCAACAAGTAATTTTTGGGTTACT 600

CF93-6 TTATCGTTATTTGCAAATGTTGATTTTTCCGTAATTTCAACAAGTAATTTTTGGGTTACT 600

CG8421 TTATCGTTATTTGCAAATGTTGATTTTTCCGTAATTTCAACAAGTAATTTTTGGGTTACT 600

**ATCC 33251** TCATCATTATTTACAAATGTTGACTTTTCCGTAATTTCAACAAGTAATTTTTGGGTTACT 600

RM1221 TCATCATTATTTGCAAATGTTGATTTTTCCGTAATTTCAACAAGTAATTTTTGGGTTACT 600

S3 TCATCATTATTTGCAAATGTTGATTTTTCCGTAATTTCAACAAGTAATTTTTGGGTTACT 600

HB93-13 TCATCATTATTTACAAATGTTGATTTTTCCGTAATTTCAACAAGTAATTTTTGGGTTACT 600

81-176 TCATCATTATTTACAAATGTTGATTTTTCCGTAATTTCAACAAGTAATTTTTGGGTTACT 600

1336 TCATCATTATTTACAAATGTTGATTTTTCCGTAATTTCAACAAGTAATTTTTGGGTTACT 600

414 TCATCATTATTTACAAATGTCGATCTTTCTATAATCTCAACAAGTAATTTTTGGGTTACT 600

* *** ****** ******* ** **** **** ************************

260.94 TTATGGCTTGTTATTCCTGTGATGGTATTTAGTTTTAATCACTCTCCTATTATTTCTTCA 660

ICDCCJ07001 TTATGGCTTGTTATTCCTGTGATGGTATTTAGTTTTAATCACTCTCCTATTATTTCTTCA 660

81116 TTATGGCTTGTTATTCCTGTGATGGTATTTAGTTTTAATCACTCTCCTATTATTTCTTCA 660

M1 TTATGGCTTGTTATTCCTGTGATGGTATTTAGTTTTAATCACTCTCCTATTATTTCTTCA 660

327 TTATGGCTTGTTATTCCTGTGATGGTATTTAGTTTTAATCACTCTCCTATTATTTCTTCA 660

DFVF1099 TTATGGCTTGTTATTCCTGTGATGGTATTTAGTTTTAATCACTCTCCTATCATTTCTTCA 660

305 TTATGGCTTGTTATTCCTGTGATGGTATTTAGTTTTAATCACTCTCCTATCATTTCTTCA 660

IA3902 TTATGGCTTGTTATTCCTGTGATGGTATTTAGTTTTAATCACTCTCCTATCATTTCTTCA 660

NCTC 11168 TTATGGCTTGTTATTCCTGTGATGGTATTTAGTTTTAATCACTCTCCTATCATTTCTTCA 660

CG8486 TTATGGCTTGTTATTCCTGTGATGGTATTTAGTTTTAATCACTCTCCTATCATTTCTTCA 660

84-25 TTATGGCTTGTTATTCCTGTGATGGTATTTAGTTTTAATCACTCTCCTATCATTTCTTCA 660

CF93-6 TTATGGCTTGTTATTCCTGTGATGGTATTTAGTTTTAATCACTCTCCTATCATTTCTTCA 660

CG8421 TTATGGCTTGTTATTCCTGTGATGGTATTTAGTTTTAATCACTCTCCTATCATTTCTTCA 660

**ATCC 33251** TTATGGCTTGTTATTCCTGTGATGGTATTTAGTTTTAATCACTCTCCTATTATTTCTTCA 660

RM1221 TTATGGCTTGTTATTCCTGTGATGGTATTTAGTTTTAATCACTCTCCTATCATTTCTTCA 660

S3 TTATGGCTTGTTATTCCTGTGATGGTATTTAGTTTTAATCACTCTCCTATCATTTCTTCA 660

HB93-13 TTATGGCTTGTTATTCCTGTGATGGTATTTAGTTTTAATCACTCTCCTATTATTTCTTCA 660

81-176 TTATGGCTTGTTATTCCTGTGATGGTATTTAGTTTTAATCACTCTCCTATTATTTCTTCA 660

1336 TTATGGCTTGTTATTCCTGTGATGGTATTTAGTTTTAATCACTCTCCTATTATTTCTTCA 660

414 TTATGGCTTGTTATTCCTGTGATGGTTTTTAGCTTTAATCACTCTCCTATTATTTCTTCA 660

************************** ***** ***************** *********

260.94 CTTGCTTGTTATTGTAAAAAAGAATATGGTGATTATGCTGAACCTCGCGCTAGAAAAATT 720

ICDCCJ07001 CTTGCTTGTTATTGTAAAAAAGAATATGGTGATTATGCTGAACCTCGCGCTAGAAAAATT 720

81116 CTTGCTTGTTATTGTAAAAAAGAATATGGTGATTATGCTGAGCCTCGCGCTAGAAAAATT 720

M1 CTTGCTTGTTATTGTAAAAAAGAATATGGTGATTATGCTGAGCCTCGCGCTAGAAAAATT 720

327 CTTGCTTGTTATTGTAAAAAAGAATATGGTGATTATGCTGAGCCTCGCGCTAGAAAAATT 720

DFVF1099 CTTGCTTGTTATTGTAAAAAAGAATATGGTGGTTATGCTGAACCTCGCGCTAGAAAAATT 720

305 CTTGCTTGTTATTGTAAAAAAGAATATGGTGGTTATGCTGAACCTCGCGCTAGAAAAATT 720

IA3902 CTTGCTTGTTATTGTAAAAAAGAATATGGTGGTTATGCTGAACCTCGCGCTAGAAAAATT 720

NCTC 11168 CTTGCTTGTTATTGTAAAAAAGAATATGGTGGTTATGCTGAACCTCGCGCTAGAAAAATT 720

CG8486 CTTGCTTGTTATTGTAAAAAAGAATATGGTGGTTATGCTGAACCTCGCGCTAGAAAAATT 720

84-25 CTTGCTTGTTATTGTAAAAAAGAATATGGTGGTTATGCTGAACCTCGCGCTAGAAAAATT 720

CF93-6 CTTGCTTGTTATTGTAAAAAAGAATATGGTGGTTATGCTGAACCTCGCGCTAGAAAAATT 720

CG8421 CTTGCTTGTTATTGTAAAAAAGAATATGGTGGTTATGCTGAACCTCGCGCTAGAAAAATT 720

**ATCC 33251** CTTGCTTGTTATTGTAAAAAAGAATATGGTGGTTATGCTGAACCTCGCGCTAGAAAAATT 720

RM1221 CTTGCTTGTTATTGTAAAAAAGAATATGGTGATTATGCTGAACCTCGCGCTAGAAAAATT 720

S3 CTTGCTTGTTATTGTAAAAAAGAATATGGTGATTATGCTGAACCTCGCGCTAGAAAAATT 720

HB93-13 CTTGCTTGTTATTGTAAAAAAGAATATGGTGATTATGCTGAACCTCGCGCTAGAAAAATT 720

81-176 CTTGCTTGTTATTGTAAAAAAGAATATGGTGATTATGCTGAACCTCGCGCTAGAAAAATT 720

1336 CTTGCTTGTTATTGTAAAAAAGAATATGGTGATTATGCTGAACCTCGCGCTAGAAAAATT 720

414 CTTGCTTGTTATTGTAAAAAAGAATATGGTGATTATGCCGAACCTCGTGCTAGAAAAATT 720

******************************* ****** ** ***** ************

260.94 ATTTCTTTGGCGATTATTCTTATGGTTTTTGTTGTAATGTTTTTTGTTTTTTCTTGTGCT 780

ICDCCJ07001 ATTTCTTTGGCGATTATTCTTATGGTTTTTGTTGTAATGTTTTTTGTTTTTTCTTGTGCT 780

81116 ATTTCTTTGGCAATTATTCTTATGGTTTTTGTTGTAATGTTTTTTGTTTTTTCTTGTGCT 780

M1 ATTTCTTTGGCAATTATTCTTATGGTTTTTGTTGTAATGTTTTTTGTTTTTTCTTGTGCT 780

327 ATTTCTTTGGCAATTATTCTTATGGTTTTTGTTGTAATGTTTTTTGTTTTTTCTTGTGCT 780

DFVF1099 ATTTCTTTGGCAATTATTCTTATGGTTTTTGTTGTAATGTTTTTTGTTTTTTCTTGTGCT 780

305 ATTTCTTTGGCAATTATTCTTATGGTTTTTGTTGTAATGTTTTTTGTTTTTTCTTGTGCT 780

IA3902 ATTTCTTTGGCAATTATTCTTATGGTTTTTGTTGTAATGTTTTTTGTTTTTTCTTGTGCT 780

NCTC 11168 ATTTCTTTGGCAATTATTCTTATGGTTTTTGTTGTAATGTTTTTTGTTTTTTCTTGTGCT 780

CG8486 ATTTCTTTGGCAATTATTCTTATGGTTTTTGTTGTAATGTTTTTTGTTTTTTCTTGTGCT 780

84-25 ATTTCTTTGGCAATTATTCTTATGGTTTTTGTTGTAATGTTTTTTGTTTTTTCTTGTGCT 780

CF93-6 ATTTCTTTGGCAATTATTCTTATGGTTTTTGTTGTAATGTTTTTTGTTTTTTCTTGTGCT 780

CG8421 ATTTCTTTAGCAATTATTCTTATGGTTTTTGTTGTAATGTTTTTTGTTTTTTCTTGTGCT 780

**ATCC 33251** ATTTCTTTGGCAATTATTCTTATGGTTTTTGTTGTAATGTTTTTTGTTTTTTCTTGTGCT 780

RM1221 ATTTCTTTGGCAATTATTCTTATGGTTTTTGTTGTAATGTTTTTTGTTTTTTCTTGTGCT 780

S3 ATTTCTTTGGCAATTATTCTTATGGTTTTTGTTGTAATGTTTTTTGTTTTTTCTTGTGCT 780

HB93-13 ATTTCTTTGGCAATTATTCTTATGGTTTTTGTTGTAATGTTTTTTGTTTTTTCTTGTGCT 780

81-176 ATTTCTTTGGCAATTATTCTTATGGTTTTTGTTGTAATGTTTTTTGTTTTTTCTTGTGCT 780

1336 ATTTCTTTGGCAATTATTCTTATGGTTTTTGTTGTAATGTTTTTTGTTTTTTCTTGCGCT 780

414 ATTTCTTTAGCAGTTGTTCTTATGGTTTTTGTTGTAATGTTTTTTGTTTTTTCTTGCGCC 780

******** ** ** **************************************** **

260.94 TTAACCTTTACACCAGAAGATTTTGCATCAGCAAAAGATCAAAATATCAATATTCTTACT 840

ICDCCJ07001 TTAACCTTTACACCAGAAGATTTTGCATCAGCAAAAGATCAAAATATCAATATTCTTACT 840

81116 TTAACCTTTACACCAGAAGATTTTGCATCAGCAAAAGATCAAAATATCAATATTCTTACT 840

M1 TTAACCTTTACACCAGAAGATTTTGCATCAGCAAAAGATCAAAATATCAATATTCTTACT 840

327 TTAACCTTTACACCAGAAGATTTTGCATCAGCAAAAGATCAAAATATCAATATTCTTACT 840

DFVF1099 TTAACCTTTACACCAGAAGATTTTGCATCAGCAAAAGATCAAAATATCAATATTCTTACT 840

305 TTAACCTTTACACCAGAAGATTTTGCATCAGCAAAAGATCAAAATATCAATATTCTTACT 840

IA3902 TTAACCTTTACACCAGAAGATTTTGCATCAGCAAAAGATCAAAATATCAATATTCTTACT 840

NCTC 11168 TTAACCTTTACACCAGAAGATTTTGCATCAGCAAAAGATCAAAATATCAATATTCTTACT 840

CG8486 TTAACCTTTACACCAGAAGATTTTGCATCAGCAAAAGATCAAAATATCAATATTCTTACT 840

84-25 TTAACCTTTACACCAGAAGATTTTGCATCAGCAAAAGATCAAAATATCAATATTCTTACT 840

CF93-6 TTAACCTTTACACCAGAAGATTTTGCATCAGCAAAAGATCAAAATATCAATATTCTTACT 840

CG8421 TTAACCTTTACACCAGAAGATTTTGCATCAGCAAAAGATCAAAATATCAATATTCTTACT 840

**ATCC 33251** TTAACCTTTACACCAGAAGATTTTGCATCAGCAAAAGATCAAAATATCAATATTCTTACT 840

RM1221 TTAACCTTTACACCAGAAGATTTTGCATCAGCAAAAGATCAAAATATCAATATTCTTACT 840

S3 TTAACCTTTACACCAGAAGATTTTGCATCAGCAAAAGATCAAAATATCAATATTCTTACT 840

HB93-13 TTAACCTTTACACCAGAAGATTTTGCATCAGCAAAAGATCAAAATATCAATATTCTTACT 840

81-176 TTAACCTTTACACCAGAAGATTTTGCATCAGCAAAAGATCAAAATATCAATATTCTTACT 840

1336 TTAACTTTTACACCAGAAGATTTTGCATCAGCAAAAGATCAAAATATCAATATTCTTACC 840

414 TTAACTTTCACACCAGAAGATTTTACATCAGCAAAAGATCAAAATGTAAATATTCTTACT 840

***** ** *************** ******************** * ***********

260.94 TTTATAGCAAATAAATTTCCTGAAGTTTCTTTACTAGCTTATGTTGGACCTATTGTTGCA 900

ICDCCJ07001 TTTATAGCAAATAAATTTCCTGAAGTTTCTTTACTAGCTTATGTTGGACCTATTGTTGCA 900

81116 TTTATAGCAAATAAATTTCCTGAAGTTTCTTTACTAGCTTATGTTGGGCCTATTGTTGCG 900

M1 TTTATAGCAAATAAATTTCCTGAAGTTTCTTTACTAGCTTATGTTGGGCCTATTGTTGCG 900

327 TTTATAGCAAATAAATTTCCTGAAGTTTCTTTACTAGCTTATGTTGGGCCTATTGTTGCG 900

DFVF1099 TTTATAGCAAATAAATTTCCTGAAGTTTCTTTACTAGCTTATGTTGGACCTATTGTTGCA 900

305 TTTATAGCAAATAAATTTCCTGAAGTTTCTTTACTAGCTTATGTTGGACCTATTGTTGCA 900

IA3902 TTTATAGCAAATAAATTTCCTGAAGTTTCTTTACTAGCTTATGTTGGACCTATTGTTGCA 900

NCTC 11168 TTTATAGCAAATAAATTTCCTGAAGTTTCTTTACTAGCTTATGTTGGACCTATTGTTGCA 900

CG8486 TTTATAGCAAATAAATTTCCTGAAGTTTCTTTACTAGCTTATGTTGGACCTATTGTTGCA 900

84-25 TTTATAGCAAATAAATTTCCTGAAGTTTCTTTACTAGCTTATGTTGGACCTATTGTTGCA 900

CF93-6 TTTATAGCAAATAAATTTCCTGAAGTTTCTTTACTAGCTTATGTTGGACCTATTGTTGCA 900

CG8421 TTTATAGCAAATAAATTTCCTGAAGTTTCTTTACTAGCTTATGTTGGACCTATTGTTGCA 900

**ATCC 33251** TTTATAGCAAATAAATTTCCTGAAGTTTCTTTACTAGCTTATGTTGGACCTATTGTTGCA 900

RM1221 TTTATAGCAAATAAATTTCCTGAAGTTTCTTTACTAGCTTATGTTGGACCTATTGTTGCA 900

S3 TTTATAGCAAATAAATTTCCTGAAGTTTCTTTACTAGCTTATGTTGGACCTATTGTTGCA 900

HB93-13 TTTATAGCAAATAAATTTCCTGAAGTTTCTTTACTAGCTTATGTTGGGCCTATTGTTGCA 900

81-176 TTTATAGCAAATAAATTTCCTGAAGTTTCTTTACTAGCTTATGTTGGGCCTATTGTTGCA 900

1336 TTTATAGCAAATAAATTTCCTGAAGTTTCTTTGCTGACTTATGTTGGGCCTATTGTTGCA 900

414 TTTATAGCAAATAAATTTCCCGAAGTTTCTTTATTAGCTTATGTTGGACCTATTGTTGCA 900

******************** *********** * ********** ***********

260.94 CTTGTGGCTATTAGCAAAAGTTTCTTAGGGCATTATCTTGGTTCTCAAGAAGGCTTAAAT 960

ICDCCJ07001 CTTGTGGCTATTAGCAAAAGTTTCTTAGGGCATTATCTTGGTTCTCAAGAAGGCTTAAAT 960

81116 CTTGTGGCTATTAGCAAAAGTTTCTTAGGGCATTATCTTGGTTCTCAAGAAGGCTTAAAT 960

M1 CTTGTGGCTATTAGCAAAAGTTTCTTAGGGCATTATCTTGGTTCTCAAGAAGGCTTAAAT 960

327 CTTGTGGCTATTAGCAAAAGTTTCTTAGGGCATTATCTTGGTTCTCAAGAAGGCTTAAAT 960

DFVF1099 CTTGTGGCTATTAGCAAAAGTTTCTTAGGGCATTATCTTGGTTCTCAAGAAGGCTTAAAT 960

305 CTTGTGGCTATTAGCAAAAGTTTCTTAGGGCATTATCTTGGTTCTCAAGAAGGCTTAAAT 960

IA3902 CTTGTGGCTATTAGCAAAAGTTTCTTAGGGCATTATCTTGGTTCTCAAGAAGGCTTAAAT 960

NCTC 11168 CTTGTGGCTATTAGCAAAAGTTTCTTAGGGCATTATCTTGGTTCTCAAGAAGGCTTAAAT 960

CG8486 CTTGTGGCTATTAGCAAAAGTTTCTTAGGGCATTATCTTGGTTCTCAAGAAGGCTTAAAT 960

84-25 CTTGTGGCTATTAGCAAAAGTTTCTTAGGGCATTATCTTGGTTCTCAAGAAGGCTTAAAT 960

CF93-6 CTTGTGGCTATTAGCAAAAGTTTCTTAGGGCATTATCTTGGTTCTCAAGAAGGCTTAAAT 960

CG8421 CTTGTAGCTATTAGCAAAAGTTTCTTAGGGCATTATCTTGGTTCTCAAGAAGGCTTAAAT 960

**ATCC 33251** CTTGTGGCTATTAGCAAAAGTTTCTTAGGGCATTATCTTGGTTCTCAAGAAGGCTTAAAT 960

RM1221 CTTGTGGCTATTAGCAAAAGTTTCTTAGGACATTATCTTGGTTCTCAAGAAGGCTTAAAT 960

S3 CTTGTGGCTATTAGCAAAAGTTTCTTAGGACATTATCTTGGTTCTCAAGAAGGCTTAAAT 960

HB93-13 CTTGTGGCTATTAGTAAAAGTTTCTTAGGACATTATCTTGGTTCTCAAGAAGGCTTAAAT 960

81-176 CTTGTGGCTATTAGTAAAAGTTTCTTAGGACATTATCTTGGTTCTCAAGAAGGCTTAAAT 960

1336 CTTGTGGCCATTAGTAAAAGTTTCTTAGGACATTATCTTGGTTCTCAAGAAGGCTTAAAC 960

414 CTTGTGGCTATTAGTAAAAGTTTTTTAGGTCATTATCTTGGCTCTCAAGAAGGCTTAAAT 960

***** ** ***** ******** ***** *********** *****************

260.94 GGCATTTTATATAAAGCAAGTAATGGTAAAATTCAAGGTAAATTCGCTCAAACTTTAACA 1020

ICDCCJ07001 GGCATTTTATATAAAGCAAGTAATGGTAAAATTCAAGGTAAATTCGCTCAAACTTTAACA 1020

81116 GGTATTTTATATAAAGCAAGTAATGGTAAAATTCAAGGTAAATTCGCTCAAACTTTAACA 1020

M1 GGTATTTTATATAAAGCAAGTAATGGTAAAATTCAAGGTAAATTCGCTCAAACTTTAACA 1020

327 GGTATTTTATATAAAGCAAGTAATGGTAAAATTCAAGGTAAATTCGCTCAAACTTTAACA 1020

DFVF1099 GGTATTTTATATAAAGCAAGTAATGGTAGAATTCAAGGTAAATTCGCTCAAACTTTAACA 1020

305 GGTATTTTATATAAAGCAAGTAATGGTAGAATTCAAGGTAAATTCGCTCAAACTTTAACA 1020

IA3902 GGTATTTTATATAAAGCAAGTAATGGTAGAATTCAAGGTAAATTCGCTCAAACTTTAACA 1020

NCTC 11168 GGTATTTTATATAAAGCAAGTAATGGTAGAATTCAAGGTAAATTCGCTCAAACTTTAACA 1020

CG8486 GGTATTTTATATAAAGCAAGTAATGGTAGAATTCAAGGTAAATTCGCTCAAACTTTAACA 1020

84-25 GGTATTTTATATAAAGCAAGTAATGGTAGAATTCAAGGTAAATTCGCTCAAACTTTAACA 1020

CF93-6 GGTATTTTATATAAAGCAAGTAATGGTAGAATTCAAGGTAAATTCGCTCAAACTTTAACA 1020

CG8421 GGTATTTTATATAAAGCAAGTAATGGTAGAATTCAAGGTAAATTCGCTCAAACTTTAACA 1020

**ATCC 33251** GGTATTTTATATAAAGCAAGTAATGGTAGAATTCAAGGTAAATTCGCTCAAACTTTAACA 1020

RM1221 GGCATTTTATATAAAGCAAGTAATGGTAGAATTCAAGGTAAATTCGCTCAAACTTTAACA 1020

S3 GGCATTTTATATAAAGCAAGTAATGGTAGAATTCAAGGTAAATTCGCTCAAACTTTAACA 1020

HB93-13 GGCATTTTATATAAAGCAAGTAATGGTAAAATTCAAGGTAAATTCGCTCAAACTTTAACA 1020

81-176 GGCATTTTATATAAAGCAAGTAATGGTAAAATTCAAGGTAAATTCGCTCAAACTTTAACA 1020

1336 GGTATTTTATATAAAGCAAGTAATGGTAAAATTCAAGGTAAATTCGCTCAAACTTTAACA 1020

414 GGTATTTTATATAAAGCAAGTAATGGTAAAATTCAAGGCAAATTAGCTCAAACTTTAACA 1020

** ************************* ********* ***** ***************

260.94 GCGATCATTACCTTTGCTATTGCTTGGCTTGTTGCGTATAAAAATCCAAGTGTTATAGGA 1080

ICDCCJ07001 GCGATCATTACCTTTGCTATTGCTTGGCTTGTTGCGTATAAAAATCCAAGTGTTATAGGA 1080

81116 GCGATCATTACCTTTGCTATTGCTTGGCTTGTTGCGTATAAAAATCCAAGTGTTATAGGA 1080

M1 GCGATCATTACCTTTGCTATTGCTTGGCTTGTTGCGTATAAAAATCCAAGTGTTATAGGA 1080

327 GCGATCATTACCTTTGCTATTGCTTGGCTTGTTGCGTATAAAAATCCAAGTGTTATAGGA 1080

DFVF1099 GCGATCATTACCTTTGCTATCGCGTGGCTTGTTGCGTATAAAAATCCAAGTGTTATAGGA 1080

305 GCGATCATTACCTTTGCTATCGCGTGGCTTGTTGCGTATAAAAATCCAAGTGTTATAGGA 1080

IA3902 GCGATCATTACCTTTGCTATCGCGTGGCTTGTTGCGTATAAAAATCCAAGTGTTATAGGA 1080

NCTC 11168 GCGATCATTACCTTTGCTATCGCGTGGCTTGTTGCGTATAAAAATCCAAGTGTTATAGGA 1080

CG8486 GCGATCATTACCTTTGCTATCGCGTGGCTTGTTGCGTATAAAAATCCAAGTGTTATAGGA 1080

84-25 GCGATCATTACCTTTGCTATCGCGTGGCTTGTTGCGTATAAAAATCCAAGTGTTATAGGA 1080

CF93-6 GCGATCATTACCTTTGCTATCGCGTGGCTTGTTGCGTATAAAAATCCAAGTGTTATAGGA 1080

CG8421 GCGATCATTACCTTTGCTATCGCGTGGTTTGTTGCGTATAAAAATCCAAGTGTTATAGGA 1080

**ATCC 33251** GCGATCATTACCTTTGCTATCGCGTGGCTTGTTGCGTATAAAAATCCAAGTGTTATAGGA 1080

RM1221 GCGATCATTACCTTTGCTATTGCTTGGCTTGTTGCGTATAAAAATCCAAGTGTTATAGGA 1080

S3 GCGATCATTACCTTTGCTATTGCTTGGCTTGTTGCGTATAAAAATCCAAGTGTTATAGGA 1080

HB93-13 GCGATCATTACCTTTGCTATCGCTTGGCTTGTTGCGTATAAAAATCCAAGTGTTATAGGA 1080

81-176 GCGATCATTACCTTTGCTATCGCTTGGCTTGTTGCGTATAAAAATCCAAGTGTTATAGGA 1080

1336 GCGATCATTACCTTTGTTATCGCTTGGCTTGTTGCGTATAAAAATCCAAGTGTTATAGGA 1080

414 GCAGTTATTACTTTTGCTATCGCTTGGTTTGTTGCGTATAAAAATCCAAGTGTTATAGGG 1080

** * ***** **** *** ** *** *******************************

260.94 ATTATCGAGGCTATTGGTGGTCCTGTTTTGGCTATTTTGCTTTTCTTAATGCCACTTTAT 1140

ICDCCJ07001 ATTATCGAGGCTATTGGTGGTCCTGTTTTGGCTATTTTGCTTTTCTTAATGCCACTTTAT 1140

81116 ATTATTGAGGCTATTGGTGGTCCTGTTTTGGCTATTTTGCTTTTCTTAATGCCACTTTAT 1140

M1 ATTATTGAGGCTATTGGTGGTCCTGTTTTGGCTATTTTGCTTTTCTTAATGCCACTTTAT 1140

327 ATTATTGAGGCTATTGGTGGTCCTGTTTTGGCTATTTTGCTTTTCTTAATGCCACTTTAT 1140

DFVF1099 ATTATCGAGGCTATTGGTGGTCCTGTTTTGGCTATTTTGCTTTTCTTAATGCCGCTTTAT 1140

305 ATTATCGAGGCTATTGGTGGTCCTGTTTTGGCTATTTTGCTTTTCTTAATGCCGCTTTAT 1140

IA3902 ATTATCGAGGCTATTGGTGGTCCTGTTTTGGCTATTTTGCTTTTCTTAATGCCGCTTTAT 1140

NCTC 11168 ATTATCGAGGCTATTGGTGGTCCTGTTTTGGCTATTTTGCTTTTCTTAATGCCGCTTTAT 1140

CG8486 ATTATCGAGGCTATTGGTGGTCCTGTTTTGGCTATTTTGCTTTTCTTAATGCCGCTTTAT 1140

84-25 ATTATCGAGGCTATTGGTGGTCCTGTTTTGGCTATTTTGCTTTTCTTAATGCCGCTTTAT 1140

CF93-6 ATTATCGAGGCTATTGGTGGTCCTGTTTTGGCTATTTTGCTTTTCTTAATGCCGCTTTAT 1140

CG8421 ATTATCGAGGCTATTGGTGGTCCTGTTTTGGCTATTTTGCTTTTCTTAATGCCGCTTTAT 1140

**ATCC 33251** ATTATCGAGGCTATTGGTGGTCCTGTTTTGGCTATTTTGCTTTTCTTAATGCCGCTTTAT 1140

RM1221 ATTATCGAGGCTATTGGTGGCCCTGTTTTGGCTATTTTGCTTTTCTTAATGCCACTTTAT 1140

S3 ATTATCGAGGCTATTGGTGGCCCTGTTTTGGCTATTTTGCTTTTCTTAATGCCACTTTAT 1140

HB93-13 ATTATCGAGGCTATTGGTGGTCCTGTTTTGGCTATTTTGCTTTTCTTAATGCCGCTTTAT 1140

81-176 ATTATCGAGGCTATTGGTGGTCCTGTTTTGGCTATTTTGCTTTTCTTAATGCCGCTTTAT 1140

1336 ATTATTGAGGCTATTGGTGGTCCTGTTTTGGCTATTTTGCTTTTCTTGATGCCGCTTTAT 1140

414 ATTATTGAAGCTATTGGTGGTCCTGTTTTGGCTATTTTACTTTTCTTAATGCCACTTTAT 1140

***** ** *********** ***************** ******** ***** ******

260.94 TGTATTTATCGTTTTGATATTTTGGCAAGATTTCGTAATAAATTTTTAGATCTTTTTATT 1200

ICDCCJ07001 TGTATTTATCGTTTTGATATTTTGGCAAGATTTCGTAATAAATTTTTAGATCTTTTTATT 1200

81116 TGTATTTATCGTTTTGATATTTTGGCAAGATTTCGTAATAAATTTTTAGATCTTTTTATT 1200

M1 TGTATTTATCGTTTTGATATTTTGGCAAGATTTCGTAATAAATTTTTAGATCTTTTTATT 1200

327 TGTATTTATCGTTTTGATATTTTGGCAAGATTTCGTAATAAATTTTTAGATCTTTTTATT 1200

DFVF1099 TGTATTTATCGTTTTGATATTTTGGCAAGGTTTCGTAATAAATTTTTAGATCTTTTTGTT 1200

305 TGTATTTATCGTTTTGATATTTTGGCAAGGTTTCGTAATAAATTTTTAGATCTTTTTGTT 1200

IA3902 TGTATTTATCGTTTTGATATTTTGGCAAGGTTTCGTAATAAATTTTTAGATCTTTTTGTT 1200

NCTC 11168 TGTATTTATCGTTTTGATATTTTGGCAAGGTTTCGTAATAAATTTTTAGATCTTTTTGTT 1200

CG8486 TGTATTTATCGTTTTGATATTTTGGCAAGGTTTCGTAATAAATTTTTAGATCTTTTTGTT 1200

84-25 TGTATTTATCGTTTTGATATTTTGGCAAGGTTTCGTAATAAATTTTTAGATCTTTTTGTT 1200

CF93-6 TGTATTTATCGTTTTGATATTTTGGCAAGGTTTCGTAATAAATTTTTAGATCTTTTTGTT 1200

CG8421 TGTATTTATCGTTTTGATATTTTGGCAAGGTTTCGTAATAAATTTTTAGATCTTTTTGTT 1200

**ATCC 33251** TGTATTTATCGTTTTGATATTTTGGCAAGGTTTCGTAATAAATTTTTAGATCTTTTTGTT 1200

RM1221 TGTATTTATCGTTTTGATATTTTGGCAAGATTTCGTAATAAATTTTTAGATCTTTTTATT 1200

S3 TGTATTTATCGTTTTGATATTTTGGCAAGATTTCGTAATAAATTTTTAGATCTTTTTATT 1200

HB93-13 TGTATTTATCGTTTTGATATTTTGGCAAGATTTCGTAATAAATTTTTAGATCTTTTTATT 1200

81-176 TGTATTTATCGTTTTGATATTTTGGCAAGATTTCGTAATAAATTTTTAGATCTTTTTATT 1200

1336 TGTATTTATCGTTTTGATATTTTGGCAAGATTTCGTAATAAATTTTTAGATCTTTTTATT 1200

414 TGTATTTATCGTTTTGATATTTTGGCAAAATTTCGTAATAAATTTTTAGATCTTTTTATT 1200

**************************** *************************** **

260.94 TTGGTAATGGGGATAGTTGCAATTTCTGCTGCAATTCATGATCTTTTATAA 1251

ICDCCJ07001 TTGGTAATGGGGATAGTTGCAATTTCTGCTGCAATTCATGATCTTTTATAA 1251

81116 TTGGTAATGGGGATAGTTGCAATTTCTGCTGCAATTCATGATCTTTTATAA 1251

M1 TTGGTAATGGGGATAGTTGCAATTTCTGCTGCAATTCATGATCTTTTATAA 1251

327 TTGGTAATGGGGATAGTTGCAATTTCTGCTGCAATTCATGATCTTTTATAA 1251

DFVF1099 TTGGTAATGGGGATAGTTGCAATTTCTGCTGCAATTCATGATCTTTTATAA 1251

305 TTGGTAATGGGGATAGTTGCAATTTCTGCTGCAATTCATGATCTTTTATAA 1251

IA3902 TTGGTAATGGGGATAGTTGCAATTTCTGCTGCAATTCATGATCTTTTATAA 1251

NCTC 11168 TTGGTAATGGGGATAGTTGCAATTTCTGCTGCAATTCATGATCTTTTATAA 1251

CG8486 TTGGTAATGGGGATAGTTGCAATTTCTGCTGCAATTCATGATCTTTTATAA 1251

84-25 TTGGTAATGGGGATAGTTGCAATTTCTGCTGCAATTCATGATCTTTTATAA 1251

CF93-6 TTGGTAATGGGGATAGTTGCAATTTCTGCTGCAATTCATGATCTTTTATAA 1251

CG8421 TTGGTAATGGGGATAGTTGCAATTTCTGCTGCAATTCATGATCTTTTATAA 1251

**ATCC 33251** TTGGTAATGGGGATAGTTGCAATTTCTGCTGCAATTCACGATCTTTTATAA 1251

RM1221 TTGGTAATGGGGATAGTTGCAATTTCTGCTGCAATTCATGATCTTTTATAA 1251

S3 TTGGTAATGGGGATAGTTGCAATTTCTGCTGCAATTCATGATCTTTTATAA 1251

HB93-13 TTGGTAATGGGGATAGTTGCAATTTCTGCTGCAATTCATGATCTTTTATAA 1251

81-176 TTGGTAATGGGGATAGTTGCAATTTCTGCTGCAATTCATGATCTTTTATAA 1251

1336 TTGGTAATGGGAATAGTTGCAATTTCTGCTGCAATTCACAATCTTTTATAA 1251

414 TTGGTAATGGGGATAGTTGCAATTTCTGCTGCAATTCATAATCTTTTATAA 1251

*********** ************************** ***********

**Figure S2. Nucleotide sequence comparison of the serine transporter *sdaC*  genes in different *C. jejuni* isolates.**
