# Supplementary material for: Contribution of Amino Acid Catabolism to the Tissue Specific Persistence of Campylobacter jejuni in a Murine Colonization Model
Source: PLoS One. 2012 Nov 30;7(11):e50699. doi: 10.1371/journal.pone.0050699 (PMC3511319; doi:10.1371/journal.pone.0050699)
Supplement: Figure S3 — Amino acid sequence comparison of SdaA proteins from different C. jejuni strains. ClustaW (www.ebi.ac.uk/Tools/msa/clustalW2) was used for the alignment of the SdaA amino acid sequences from various C. jejuni isolates. The accession numbers were as follows: CF93-6 (CJJCF936_1718; ZP_01067690), 84-25 (CJJ8425_1708; ZP_01099834), NCTC 11168 (Cj1624c; YP_002344993), IA3902 (CJSA_1536; ADC29171), DFVF1099 (CSQ_0902, this study), 305 (CSS_1725; this study), CG8486 (Cj8486_1666c; ZP_01809456), RM1221 (CJE1796; YP_179767), S3 (CJS3_1705; ADT73404), 260.94 (CJJ26094_1675; NZ_AANK01000006.1), 81-176 (CJJ81176_1615; YP_001001267), 1336 (C1336_000330073; ZP_06374482), HB93-13 (CJJHB9313_1615; ZP_01070837), M1 (CJM1_1565; ADN91750), 81116 (C8J_1526; YP_001483100), ICDCCJ07001 (ICDCCJ07001_1539; YP_004067035), 327 (CSU_0676; this study), CG8421 (Cj8421_1678; ZP_03222408), ATCC 33251 (this study), 414 (C414_000010127; ZP_06371291). (DOC) [file pone.0050699.s003.doc]

CF93-6 MSNLSIFKIGVGPSSSHTLGPMLAGNLFCKKVAKKLDEIDRVEVTLYGSLSLTGKGHLSD 60

84-25 MSNLSIFKIGVGPSSSHTLGPMLAGNLFCKKVAKKLDEIDRVEVTLYGSLSLTGKGHLSD 60

NCTC 11168 MSNLSIFKIGVGPSSSHTLGPMLAGNLFCKKVAKKLDEIDRVEVTLYGSLSLTGKGHLSD 60

IA3902 MSNLSIFKIGVGPSSSHTLGPMLAGNLFCKKVAKKLDEIDRVEVTLYGSLSLTGKGHLSD 60

DFVF1099 MSNLSIFKIGVGPSSSHTLGPMLAGNLFCKKVAKKLDEIDRVEVTLYGSLSLTGKGHLSD 60

305 MSNLSIFKIGVGPSSSHTLGPMLAGNLFCKKVAKKLDEIDRVEVTLYGSLSLTGKGHLSD 60

CG8486 MSNLSIFKIGVGPSSSHTLGPMLAGNLFCKKVAKKLDEIDRVEVTLYGSLSLTGKGHLSD 60

RM1221 MSNLSIFKIGVGPSSSHTLGPMLAGNLFCKKVAKKLDEIDRVEVTLYGSLSLTGKGHLSD 60

S3 MSNLSIFKIGVGPSSSHTLGPMLAGNLFCKKVAKKLDEIDRVEVTLYGSLSLTGKGHLSD 60

260.94 MSNLSIFKIGVGPSSSHTLGPMLAGNLFCKKVAKKLDEIDRVEVTLYGSLSLTGKGHLSD 60

81-176 MSNLSIFKIGVGPSSSHTLGPMLAGNLFCKKVAKKLDEIDRVEVTLYGSLSLTGKGHLSD 60

1336 MSNLSIFKIGVGPSSSHTLGPMLAGNLFCKKVAKKLDEIDRVEVTLYGSLSLTGKGHLSD 60

HB93-13 MSNLSIFKIGVGPSSSHTLGPMLAGNLFCKKVAKKLDEIDRVEVTLYGSLSLTGKGHLSD 60

M1 MSNLSIFKIGVGPSSSHTLGPMLAGNLFCKKVAKKLDEIDRVEVTLYGSLSLTGKGHLSD 60

81116 MSNLSIFKIGVGPSSSHTLGPMLAGNLFCKKVAKKLDEIDRVEVTLYGSLSLTGKGHLSD 60

ICDCCJ07001 MSNLSIFKIGVGPSSSHTLGPMLAGNLFCKKVAKKLDEIDRVEVTLYGSLSLTGKGHLSD 60

327 MSNLSIFKIGVGPSSSHTLGPMLAGNLFCKKVAKKLDEIDRVEVTLYGSLSLTGKGHLSD 60

CG8421 MSNLSIFKIGVGPSSSHTLGPMLAGNLFCKKVAKKLDEIDRVEVTLYGSLSLTGKGHLSD 60

**ATCC 33251** MSNLNIFKIGVGPSSSHTLGPMLAGNLFCKKVAKKLDEIDRVEVTLYGSLSLTGKGHLSD 60

414 MSNLSIFKIGVGPSSSHTLGPMLAGNLFCKKVAKKLDEINRVEVTLYGSLSLTGKGHLSD 60

****.**********************************:********************

CF93-6 KAVIWGLNGLEAKNLSAAIQDEVNKNAIENAQIDFCGEKKLSFNYEKDLIFSKDFLPL-- 118

84-25 KAVIWGLNGLEAKNLSAAIQDEVNKNAIENAQIDFCGEKKLSFNYEKDLIFSKDFLPL-- 118

NCTC 11168 KAVIWGLNGLEAKNLSAAIQDEVNKNAIENAQIDFCGEKKLSFNYEKDLIFSKDFLPL-- 118

IA3902 KAVIWGLNGLEAKNLSAAIQDEVNKNAIENAQIDFCGEKKLSFNYEKDLIFSKDFLPL-- 118

DFVF1099 KAVIWGLNGLEAKNLSAAIQDEVNKNAIENAQIDFCGEKKLSFNYEKDLIFSKDFLPL-- 118

305 KAVIWGLNGLEAKNLSAAIQDEVNKNAIENAQIDFCGEKKLSFNYEKDLIFSKDFLPL-- 118

CG8486 KAVIWGLNGLEAKNLSAAIQDEVNKNAIENAQIDFCGEKKLSFNYEKDLIFSKDFLPL-- 118

RM1221 KAVIWGLNGLEAKNLSTAIQDEVNKNAIENAQIDFCGEKKLCFNYEKDLIFSKDFLPL-- 118

S3 KAVIWGLNGLEAKNLSTAIQDEVNKNAIENAQIDFCGEKKLCFNYEKDLIFSKDFLPL-- 118

260.94 KAVIWGLNGLEAKNLSAAIQDEVNKNAIENAQIDFCGEKKLCFNYEKDLIFSKDFLPL-- 118

81-176 KAVIWGLNGLEAKNLSAAIQDEVNKNAIENAQIDFCGEKKLCFNYEKDLIFSKDFLPL-- 118

1336 KAVIWGLNGLEAKNLSAAIQDEVNKNAIENAQIDFCGEKKLCFNYEKDLIFSKDFLPL-- 118

HB93-13 KAVIWGLNGLEAKNLSAAIQDEVNKNAIENAQIDFCGEKKLCFNYEKDLIFSKDFLPL-- 118

M1 KAVIWGLNGLEAKNLSAAIQDEVNKNAIENAQIDFCGEKKLCFNYEKDLIFSKDFLPL-- 118

81116 KAVIWGLNGLEAKNLSAAIQDEVNKNAIENAQIDFCGEKKLCFNYEKDLIFSKDFLPL-- 118

ICDCCJ07001 KAVIWGLNGLEAKNLSAAIQDEVNKNAIENAQIDFCGEKKLCFNYEKDLIFSKDFLPL-- 118

327 KAVIWGLNGLEAKNLSAAIQDEVNKNAIENAQIDFCGEKKLCFNYEKDLIFSKDFLPL-- 118

CG8421 KAVIWGLNGLEAKNLSAAIQDEVNKNAIENAQIDFCGEKKLCFNYEKDLIFSKDFLPL-- 118

**ATCC 33251** KAVIWGLNGLEAKNLSAAIQDEVNKNAIENAQIDFCGEKKLSFNYEKDLIFSKDFLPLHV 120

414 KAVIWGLNGLEAKNLSAAIQDEVNKNAIENAQIDFCGEKKLSFNYEKDLIFSKDFLPL-- 118

****************:************************.****************

CF93-6 HENGMKIKAYDCKGGLVDEETYYSVGGGFVLTAAELEKKGKNSNQNKKKKLDIELNNAKE 178

84-25 HENGMKIKAYDCKGGLVDEETYYSVGGGFVLTAAELEKKGKNSNQNKKKKLDIELNNAKE 178

NCTC 11168 HENGMKIKAYDCKGGLVDEETYYSVGGGFVLTAAELEKKGKNSNQNKKKKLDIELNNAKE 178

IA3902 HENGMKIKAYDCKGGLVDEETYYSVGGGFVLTAAELEKKGKNSNQNKKKKLDIELNNAKE 178

DFVF1099 HENGMKIKAYDCKGGLVDEETYYSVGGGFVLTAAELEKKGKNSNQNKKKKLDIELNNAKE 178

305 HENGMKIKAYDCKGGLVDEETYYSVGGGFVLTAAELEKKGKNSNQNKKKKLDIELNNAKE 178

CG8486 HENGMKIKAYDCKGGLVDEETYYSVGGGFVLTAAELEKKGKNSNQNKKKKLDIELNNAKE 178

RM1221 HENGMKIKAYDCKGGLVDEETYYSVGGGFVLTAAQLEKKGKNSNQNKKKKLDIELNNAKE 178

S3 HENGMKIKAYDCKGGLVDEETYYSVGGGFVLTAAQLEKKGKNSNQNKKKKLDIELNNAKE 178

260.94 HENGMKIKAYDCKGGLVDEETYYSVGGGFVLTAAQLEKKGKNSNQNKKKKLDIELNNAKE 178

81-176 HENGMKIKAYDCKGGLVDEETYYSVGGGFVLTAAQLEKKGKNSNQNKKKKLDIELNNAKE 178

1336 HENGMKIKAYDCKGGLVDEETYYSVGGGFVLTAAQLEKKGKNSNQNKKKKLDIELNNAKE 178

HB93-13 HENGMKIKAYDCKGGLVDEETYYSVGGGFVLTAAQLEKKGKNSNQNKKKKLDIELNNAKE 178

M1 HENGMKIKAYDCKGGLVDEETYYSVGGGFVLTAAQLEKKGKNSNQNKKKKLDIELNNAKE 178

81116 HENGMKIKAYDCKGGLVDEETYYSVGGGFVLTAAQLEKKGKNSNQNKKKKLDIELNNAKE 178

ICDCCJ07001 HENGMKIKAYDCKGGLVDEETYYSVGGGFVLTAAQLEKKGKNSNQNKKKKLDIELNNAKE 178

327 HENGMKIKAYDCKGGLVDEETYYSVGGGFVLTAAQLEKKGKNSNQNKKKKLDIELNNAKE 178

CG8421 HENGMKIKAYDCKGGLVDEETYYSVGGGFVLTAAELEKKDKNSNQNKKKKLDIELNNAKE 178

**ATCC 33251** HENGMKIKAYDCKGGLVDEETYYSVGGGFVLTAAELEKKGKNSNQNKKKKLDIELNNAKE 180

414 HENGMKIKAYDCKGALVDEETYYSVGGGFVLTAAELEKEGKNSNQNKKKKLDIELNNAKE 178

**************.*******************:***:.********************

CF93-6 ALELCDKRDWDLAELSYRYELQFHTKEEICAYCLEIWEVMQEVYYNGTHPNEDYLPGKLH 238

84-25 ALELCDKRDWDLAELSYRYELQFHTKEEICAYCLEIWEVMQEVYYNGTHPNEDYLPGKLH 238

NCTC 11168 ALELCDKRDWDLAELSYRYELQFHTKEEICAYCLEIWEVMQEVYYNGTHPNEDYLPGKLH 238

IA3902 ALELCDKRDWDLAELSYRYELQFHTKEEICAYCLEIWEVMQEVYYNGTHPNEDYLPGKLH 238

DFVF1099 ALELCDKRDWDLAELSYRYELQFHTKEEICAYCLEIWEVMQEVYYNGTHPNEDYLPGKLH 238

305 ALELCDKRDWDLAELSYRYELQFHTKEEICAYCLEIWEVMQEVYYNGTHPNEDYLPGKLH 238

CG8486 ALELCDKRDWDLAELSYRYELQFHTKEEIRAYCLEIWEVMQEVYYNGTHPNEDYLPGKLH 238

RM1221 ALELCDKRDWDLAELSYRYELQFHTKEEIRAYCLEIWEVMQEVYYNGTHPNEDYLPGKLH 238

S3 ALELCDKRDWDLAELSYRYELQFHTKEEIRAYCLEIWEVMQEVYYNGTHPNEDYLPGKLH 238

260.94 ALELCDKRDWDLAELSYRYELQFHTKEEIRAYCLEIWEVMQEVYYNGTHPNEDYLPGKLH 238

81-176 ALELCDKRDWDLAELSYRYELQFHTKEEIRAYCLEIWEVMQEVYYNGTHPNEDYLPGKLH 238

1336 ALELCDKRDWDLAELSYRYELQFHTKEEIRAYCLEIWEVMQEVYYNGTHPNEDYLPGKLH 238

HB93-13 ALELCDKRDWDLAELSYRYELQFHTKEEIRAYCLEIWEVMQEVYYNGTHPNEDYLPGKLH 238

M1 ALELCDKRDWDLAELSYRYELQFHTKEEIRAYCLEIWEVMQEVYYSGTHPNEDYLPGKLH 238

81116 ALELCDKRDWDLAELSYRYELQFHTKEEIRAYCLEIWEVMQEVYYNGTHPNEDYLPGKLH 238

ICDCCJ07001 ALELCDKRDWDLAELSYRYELQFHTKEEIRAYCLEIWEVMQEVYYNGTHPNEDYLPGKLH 238

327 ALELCDKRDWDLAELSYRYELQFHTKEEIRAYCLEIWEVMQEVYYNGTHPNEDYLPGKLH 238

CG8421 ALELCDKRDWDLAELSYRYELQFHTKEEIRAYCLEIWEVMQEVYYNGTHPNEDYLPGKLH 238

**ATCC 33251** ALELCDKRDWDLAELSYRYELQFHTKEEIRAYCLEIWEVMQEVYYNGTHPNEDYLPGKLH 240

414 ALELCDKRDWDLAELSYRYELQFHTKEEIRAYCLEIWEVMQEVYYNGTHPNEDYLPGKLH 238

***************************** ***************.**************

CF93-6 LKRRAKGLKERVAMTADPMGIIDFISLYAIAIAEENASGAKVVTAPTNGACAVIPAVMLY 298

84-25 LKRRAKGLKERVAMTADPMGIIDFISLYAIAIAEENASGAKVVTAPTNGACAVIPAVMLY 298

NCTC 11168 LKRRAKGLKERVAMTADPMGIIDFISLYAIAIAEENASGAKVVTAPTNGACAVIPAVMLY 298

IA3902 LKRRAKGLKERVAMTADPMGIIDFISLYAIAIAEENASGAKVVTAPTNGACAVIPAVMLY 298

DFVF1099 LKRRAKGLKERVAMTADPMGIIDFISLYAIAIAEENASGAKVVTAPTNGACAVIPAVMLY 298

305 LKRRAKGLKERVAMTADPMGIIDFISLYAIAIAEENASGAKVVTAPTNGACAVIPAVMLY 298

CG8486 LKRRAKGLKERVAMTADPMGIIDFISLYAIAIAEENASGAKVVTAPTNGACAVIPAVMLY 298

RM1221 LKRRAKGLKERVAMTADPMGIIDFISLYAIAIAEENASGAKVVTAPTNGACAVIPAVMLY 298

S3 LKRRAKGLKERVAMTADPMGIIDFISLYAIAIAEENASGAKVVTAPTNGACAVIPAVMLY 298

260.94 LKRRAKGLKERVAMTADPMGIIDFISLYAIAIAEENASGAKVVTAPTNGACAVIPAVMLY 298

81-176 LKRRAKGLKERVAMTADPMGIIDFISLYAIAIAEENASGAKVVTAPTNGACAVIPAVMLY 298

1336 LKRRAKGLKERVAMTADPMGIIDFISLYAIAIAEENASGAKVVTAPTNGACAVIPAVMLY 298

HB93-13 LKRRAKGLKERVAMTADPMGIIDFISLYAIAIAEENASGAKVVTAPTNGACAVIPAVMLY 298

M1 LKRRAKGLKERVAMTADPMGIIDFISLYAIAIAEENASGAKVVTAPTNGACAVIPAVMLY 298

81116 LKRRAKGLKERVAMTADPMGIIDFISLYAIAIAEENASGAKVVTAPTNGACAVIPAVMLY 298

ICDCCJ07001 LKRRAKGLKERVAMTADPMGIIDFISLYAIAIAEENASGAKVVTAPTNGACAVIPAVMLY 298

327 LKRRAKGLKERVAMTADPMGIIDFISLYAIAIAEENASGAKVVTAPTNGACAVIPAVMLY 298

CG8421 LKRRAKGLKERVAMTADPMGIIDFISLYAIAIAEENASGAKVVTAPTNGACAVIPAVMLY 298

**ATCC 33251** LKRRAKGLKERVAMTADPMGIIDFISLYAIAIAEENASGAKVVTAPTNGACAVIPAVMLY 300

414 LKRRAKGLKKRVAMTADPMGIIDFISLYAIAIAEENASGAKVVTAPTNGACAVIPAVMLY 298

*********:**************************************************

CF93-6 LKNHTIGFSDEKVIEFLLTAMLIGSFYKKNASISGAEAGCQAEIGSASSMAAAAMATVLG 358

84-25 LKNHTIGFSDEKVIEFLLTAMLIGSFYKKNASISGAEAGCQAEIGSASSMAAAAMATVLG 358

NCTC 11168 LKNHTIGFSDEKVIEFLLTAMLIGSFYKKNASISGAEAGCQAEIGSASSMAAAAMATVLG 358

IA3902 LKNHTIGFSDEKVIEFLLTAMLIGSFYKKNASISGAEAGCQAEIGSASSMAAAAMATVLG 358

DFVF1099 LKNHTIGFSDEKVIEFLLTAMLIGSFYKKNASISGAEAGCQAEIGSASSMAAAAMATVLG 358

305 LKNHTIGFSDEKVIEFLLTAMLIGSFYKKNASISGAEAGCQAEIGSASSMAAAAMATVLG 358

CG8486 LKNHTIGFSDEKAIEFLLTAMLIGSFYKKNASISGAEAGCQAEIGSASSMAAAAMATVLG 358

RM1221 LKNHTIGFSDEKAIEFLLTAMLIGSFYKKNASISGAEAGCQAEIGSASSMAAAAMATVLG 358

S3 LKNHTIGFSDEKAIEFLLTAMLIGSFYKKNASISGAEAGCQAEIGSASSMAAAAMATVLG 358

260.94 LKNHTIGFSDEKAIEFLLTAMLIGSFYKKNASISGAEAGCQAEIGSASSMAAAAMATVLG 358

81-176 LKNHTIGFSDEKAIEFLLTAMLIGSFYKKNASISGAEAGCQAEIGSASSMAAAAMATVLG 358

1336 LKNHTIGFSDEKAIEFLLTAMLIGSFYKKNASISGAEAGCQAEIGSASSMAAAAMATVLG 358

HB93-13 LKNHTIGFSDEKAIEFLLTAMLIGSFYKKNASISGAEAGCQAEIGSASSMAAAAMATVLG 358

M1 LKNHTIGFSDEKAIEFLLTAMLIGSFYKKNASISGAEAGCQAEIGSASSMAAAAMATVLG 358

81116 LKNHTIGFSDEKAIEFLLTAMLIGSFYKKNASISGAEAGCQAEIGSASSMAAAAMATVLG 358

ICDCCJ07001 LKNHTIGFSDEKAIEFLLTAMLIGSFYKKNASISGAEAGCQAEIGSASSMAAAAMATVLG 358

327 LKNHTIGFSDEKAIEFLLTAMLIGSFYKKNASISGAEAGCQAEIGSASSMAAAAMATVLG 358

CG8421 LKNHTIGFSDEKAIEFLLTAMLIGSFYKKNASISGAEAGCQAEIGSASSMAAAAMATVLG 358

**ATCC 33251** LKNHTIGFSDEKAIEFLLTAMLIGSFYKKNASISGAEAGCQAEIGSASSMAAAAMATVLG 360

414 LKNHTIGFSDEKAIEFLLTAMLIGSFYKKNASISGAEAGCQAEIGSASSMAAAAMATVLG 358

************.***********************************************

CF93-6 ANAFKACNAAEMAMEHHLGLTCDPVAGLVQIPCIERNAFGAIKAISAARMAMTRKSTPMV 418

84-25 ANAFKACNAAEMAMEHHLGLTCDPVAGLVQIPCIERNAFGAIKAISAARMAMTRKSTPMV 418

NCTC 11168 ANAFKACNAAEMAMEHHLGLTCDPVAGLVQIPCIERNAFGAIKAISAARMAMTRKSTPMV 418

IA3902 ANAFKACNAAEMAMEHHLGLTCDPVAGLVQIPCIERNAFGAIKAISAARMAMTRKSTPMV 418

DFVF1099 ANAFKACNAAEMAMEHHLGLTCDPVAGLVQIPCIERNAFGAIKAISAARMAMTRKSTPMV 418

305 ANAFKACNAAEMAMEHHLGLTCDPVAGLVQIPCIERNAFGAIKAISAARMAMTRKSTPMV 418

CG8486 VNAFKACNAAEMAMEHHLGLTCDPVAGLVQIPCIERNAFGAIKAISAARMAMTRKSTPMV 418

RM1221 ANAFKACNAAEMAMEHHLGLTCDPVAGLVQIPCIERNAFGAIKAISAARMAMTRKSTPMV 418

S3 ANAFKACNAAEMAMEHHLGLTCDPVAGLVQIPCIERNAFGAIKAISAARMAMTRKSTPMV 418

260.94 ANAFKACNAAEMAMEHHLGLTCDPVAGLVQIPCIERNAFGAIKAISAARMAMTRKSTPMV 418

81-176 ANAFKACNAAEMAMEHHLGLTCDPVAGLVQIPCIERNAFGAIKAISAARMAMTRKSTPMV 418

1336 ANAFKACNAAEMAMEHHLGLTCDPVAGLVQIPCIERNAFGAIKAISAARMAMTRKSTPMV 418

HB93-13 ANAFKACNAAEMAMEHHLGLTCDPVAGLVQIPCIERNAFGAIKAISAARMAMTRKSTPMV 418

M1 ANAFKACNAAEMAMEHHLGLTCDPVAGLVQIPCIERNAFGAIKAISAARMAMTRKSTPMV 418

81116 ANAFKACNAAEMAMEHHLGLTCDPVAGLVQIPCIERNAFGAIKAISAARMAMTRKSTPMV 418

ICDCCJ07001 ANAFKACNAAEMAMEHHLGLTCDPVAGLVQIPCIERNAFGAIKAISAARMAMTRKSTPMV 418

327 ANAFKACNAAEMAMEHHLGLTCDPVAGLVQIPCIERNAFGAIKAISAARMAMTRKSTPMV 418

CG8421 ANAFKACNAAEMAMEHHLGLTCDPVAGLVQIPCIERNAFGAIKAISAARMAMTRKSTPMV 418

**ATCC 33251** ANAFKACNAAEMAMEHHLGLTCDPVAGLVQIPCIERNAFGAIKAISAARMAMTRKSTPMV 420

414 ANAFKACNAAEMAMEHHLGLTCDPVAGLVQIPCIERNAFGAIKAISAARMTMTRKSTPMV 418

.*************************************************:*********

CF93-6 SLDEVIETMYETGKDMNYKYKETSLGGLATNLKTVC 454

84-25 SLDEVIETMYETGKDMNYKYKETSLGGLATNLKTVC 454

NCTC 11168 SLDEVIETMYETGKDMNYKYKETSLGGLATNLKTVC 454

IA3902 SLDEVIETMYETGKDMNYKYKETSLGGLATNLKTVC 454

DFVF1099 SLDEVIETMYETGKDMNYKYKETSLGGLATNLKTVC 454

305 SLDEVIETMYETGKDMNYKYKETSLGGLATNLKTVC 454

CG8486 SLDEVIETMYETGKDMNYKYKETSLGGLATNLKTVC 454

RM1221 SLDEVIETMYETGKDMNYKYKETSLGGLATNLKTVC 454

S3 SLDEVIETMYETGKDMNYKYKETSLGGLATNLKTVC 454

260.94 SLDEVIETMYETGKDMNYKYKETSLGGLATNLKTVC 454

81-176 SLDEVIETMYETGKDMNYKYKETSLGGLATNLKTVC 454

1336 SLDEVIETMYETGKDMNYKYKETSLGGLATNLKTVC 454

HB93-13 SLDEVIETMYETGKDMNYKYKETSLGGLATNLKTVC 454

M1 SLDEVIETMYETGKDMNYKYKETSLGGLATNLKTVC 454

81116 SLDEVIETMYETGKDMNYKYKETSLGGLATNLKTVC 454

ICDCCJ07001 SLDEVIETMYETGKDMNYKYKETSLGGLATNLKTVC 454

327 SLDEVIETMYETGKDMNYKYKETSLGGLATNLKTVC 454

CG8421 SLDEVIETMYETGKDMNYKYKETSLGGLATNLKTVC 454

**ATCC 33251** SLDEVIETMCETGKDMNYKYKETSLGGLATNLKTVC 456

414 SLDEVIETMYETGKDMNYKYKETSLGGLATNLKTVC 454

********* **************************

**Figure S3. Amino acid sequence comparison of SdaA proteins from different *C. jejuni* strains.**
