# Supplementary material for: Contribution of Amino Acid Catabolism to the Tissue Specific Persistence of Campylobacter jejuni in a Murine Colonization Model
Source: PLoS One. 2012 Nov 30;7(11):e50699. doi: 10.1371/journal.pone.0050699 (PMC3511319; doi:10.1371/journal.pone.0050699)
Supplement: Figure S4 — Comparison of SdaC serine transporter protein sequences in various C. jejuni isolates. ClustaW (www.ebi.ac.uk/Tools/msa/clustalW2) was used for the alignment of the SdaC amino acid sequences from listed C. jejuni isolates. The protein sequence accession numbers for the SdaC serine transporters of the different C. jejuni strains were as follows: RM1221 (CJE1797; YP_179768), S3 (CJS3_1706, ADT73405), CF93-6 (CJJCF936_1719; ZP_01067605), 84-25 (CJJ8425_1709; ZP_01099564); CG8486 (Cj8486_1667c; ZP_01809457), NCTC 11168 (Cj1625c; YP_002344994), IA3902 (CJSA_1537; ADC29172), DFVF1099 (CSQ_0900; EFV06958), 305 (CSS_1724; EFV08300); CG8421 (Cj8421_1679; ZP_03222409); ATCC 33251 (this study); 260.94 (CJJ26094_1676; ZP_01070445), HB93-13 (CJJHB9313_1616; ZP_01071370); 81-176 (CJJ81176_1616; ZP_02271921), 81116 (C8J_1527; YP_001483101), ICDCCJ07001 (ICDCCJ07001_1540; YP_004067036), M1 (CJM1_1566; ADN91751); 327 (CSU_0678; EFV10631), 1336 (C1336_000330074; ZP_06374483), 414 (C414_000010126; ZP_06371290). (DOC) [file pone.0050699.s004.doc]

RM1221 MNTPKWTSHDTRWVLSLFGTAIGAGVLLLPISAGLGGLIPLLVILVLAFPMTYLAHRNLC 60

S3 MNTPKWTSHDTRWVLSLFGTAIGAGVLLLPISAGLGGLIPLLVILVLAFPMTYLAHRNLC 60

CF93-6 MNTPKWTSHDTRWVLSLFGTAIGAGVLLLPISAGLGGLIPLLVILVLAFPMTYLAHRNLC 60

84-25 MNTPKWTSHDTRWVLSLFGTAIGAGVLLLPISAGLGGLIPLLVILVLAFPMTYLAHRNLC 60

CG8486 MNTPKWTSHDTRWVLSLFGTAIGAGVLLLPISAGLGGLIPLLVILVLAFPMTYLAHRNLC 60

NCTC 11168 MNTPKWTSHDTRWVLSLFGTAIGAGVLLLPISAGLGGLIPLLVILVLAFPMTYLAHRNLC 60

IA3902 MNTPKWTSHDTRWVLSLFGTAIGAGVLLLPISAGLGGLIPLLVILVLAFPMTYLAHRNLC 60

DFVF1099 MNTPKWTSHDTRWVLSLFGTAIGAGVLLLPISAGLGGLIPLLVILVLAFPMTYLAHRNLC 60

305 MNTPKWTSHDTRWVLSLFGTAIGAGVLLLPISAGLGGLIPLLVILVLAFPMTYLAHRNLC 60

CG8421 MNTPKWTSHDTRWVLSLFGTAIGAGVLLLPISAGLGGLIPLLVILVLAFPMTYLAHRNLC 60

**ATCC 33251** MNTPKWTSHDTRWVLSLFGTAIGAGVLLLPISAGLGGLIPLLVILVLAFPMTYLAHRNLC 60

260.94 MNTPKWTSHDTRWVLSLFGTAIGAGVLLLPISAGLGGLIPLLVILVLAFPMTYLAHRNLC 60

HB93-13 MNTPKWTSHDTRWVLSLFGTAIGAGVLLLPISAGLGGLIPLLVILVLAFPMTYLAHRNLC 60

81-176 MNTPKWTSHDTRWVLSLFGTAIGAGVLLLPISAGLGGLIPLLVILVLAFPMTYLAHRNLC 60

81116 MNTPKWTSHDTRWVLSLFGTAIGAGVLLLPISAGLGGLIPLLVILVLAFPMTYLAHRNLC 60

ICDCCJ07001 MNTPKWTSHDTRWVLSLFGTAIGAGVLLLPISAGLGGLIPLLVILVLAFPMTYLAHRNLC 60

M1 MNTPKWTSHDTRWVLSLFGTAIGAGVLLLPISAGLGGLIPLLVILVLAFPMTYLAHRNLC 60

327 MNTPKWTSHDTRWVLSLFGTAIGAGVLLLPISAGLGGLIPLLVILVLAFPMTYLAHRNLC 60

1336 MNTPKWTSHDTRWVLSLFGTAIGAGVLLLPISAGLGGLIPLLVILVLAFPMTYLAHRNLC 60

414 MNTPKWTSHDTRWVLSLFGTAIGAGVLLLPISAGLGGLIPLLIILVLAFPMTYLAHRNLC 60

******************************************:*****************

RM1221 RFVLSSSNPKDDITFVAESYFGKGGGFLITLLYFFAILPILLVYSANLTTTLLEFLINQF 120

S3 RFVLSSSNPKDDITFVAESYFGKGGGFLITLLYFFAILPILLVYSANLTTTLLEFLINQF 120

CF93-6 RFVLSSSNPKDDITFVAESYFGKGGGFLITLLYFFAILPILLVYSANLTTTLLEFLINQF 120

84-25 RFVLSSSNPKDDITFVAESYFGKGGGFLITLLYFFAILPILLVYSANLTTTLLEFLINQF 120

CG8486 RFVLSSSNPKDDITFVAESYFGKGGGFLITLLYFFAILPILLVYSANLTTTLLEFLINQF 120

NCTC 11168 RFVLSSSNPKDDITFVAESYFGKGGGFLITLLYFFAILPILLVYSANLTTTLLEFLINQF 120

IA3902 RFVLSSSNPKDDITFVAESYFGKGGGFLITLLYFFAILPILLVYSANLTTTLLEFLINQF 120

DFVF1099 RFVLSSSNPKDDITFVAESYFGKGGGFLITLLYFFAILPILLVYSANLTTTLLEFLINQF 120

305 RFVLSSSNPKDDITFVAESYFGKGGGFLITLLYFFAILPILLVYSANLTTTLLEFLINQF 120

CG8421 RFVLSSSNPKDDITFVAESYFGKGGGFLITLLYFFAILPILLVYSANLTTTLLEFLINQF 120

**ATCC 33251** RFVLSSSNPKDDITFVAESYFGKGGGFLITLLYFFAILPILLVYSANLTTTLLEFLINQF 120

260.94 RFVLSSSNPKDDITFVAESYFGKGGGFLITLLYFFAILPILLVYSANLTTTLLEFLINQF 120

HB93-13 RFVLSSSNPKDDITFVAESYFGKGGGFLITLLYFFAILPILLVYSANLTTTLLEFLINQF 120

81-176 RFVLSSSNPKDDITFVAESYFGKGGGFLITLLYFFAILPILLVYSANLTTTLLEFLINQF 120

81116 RFVLSSSNPKDDITFVAESYFGKGGGFLITLLYFFAILPILLVYSANLTTTLLEFLINQF 120

ICDCCJ07001 RFVLSSSNPKDDITFVAESYFGKGGGFLITLLYFFAILPILLVYSANLTTTLLEFLINQF 120

M1 RFVLSSSNPKDDITFVAESYFGKGGGFLITLLYFFAILPILLVYSANLTTTLLEFLINQF 120

327 RFVLSSSNPKDDITFVAESYFGKGGGFLITLLYFFAILPILLVYSANLTTTLLEFLINQF 120

1336 RFVLSSSNPKDDITFVAESYFGKGGGFLITLLYFFAILPILLVYSANLTTTLLEFLINQF 120

414 RFVLSSSNPKDDITFVAESYFGKGGGFLITLLYFFAILPILLVYSANLTTTLLEFLINQF 120

************************************************************

RM1221 NFNADLTHAARWWVSFLIVGVLVLISILGENVVTKAMSFLVFPFIIFLFIFSLLLIPQWN 180

S3 NFNADLTHAARWWVSFLIVGVLVLISILGENVVTKAMSFLVFPFIIFLFIFSLLLIPQWN 180

CF93-6 NFNADLTHAARWWVSFLIVGVLVLISILGENVVTKAMSFLVFPFIIFLFIFSLLLIPQWN 180

84-25 NFNADLTHAARWWVSFLIVGVLVLISILGENVVTKAMSFLVFPFIIFLFIFSLLLIPQWN 180

CG8486 NFNADLTHAARWWVSFLIVGVLVLISILGENVVTKAMSFLVFPFIIFLFIFSLLLIPQWN 180

NCTC 11168 NFNADLTHAARWWVSFLIVGVLVLISILGENVVTKAMSFLVFPFIIFLFIFSLLLIPQWN 180

IA3902 NFNADLTHAARWWVSFLIVGVLVLISILGENVVTKAMSFLVFPFIIFLFIFSLLLIPQWN 180

DFVF1099 NFNADLTHAARWWVSFLIVGVLVLISILGENVVTKAMSFLVFPFIIFLFIFSLLLIPQWN 180

305 NFNADLTHAARWWVSFLIVGVLVLISILGENVVTKAMSFLVFPFIIFLFIFSLLLIPQWN 180

CG8421 NFNADLTHAARWWVSFLIVGVLVLISILGENVVTKAMSFLVFPFIIFLFIFSLLLIPQWN 180

**ATCC 33251** NFNADLTHAARWWVSFLIVGVLVLISILGENVVTKAMSFLVFPFIIFLFIFSLLLIPQWN 180

260.94 NFNADLTHAARWWVSFLIVGVLVLISILGENVVTKAMSFLVFPFIIFLFIFSLLLIPQWN 180

HB93-13 NFNADLTHAARWWVSFLIVGVLVLISILGENVVTKAMSFLVFPFIIFLFIFSLLLIPQWN 180

81-176 NFNADLTHAARWWVSFLIVGVLVLISILGENVVTKAMSFLVFPFIIFLFIFSLLLIPQWN 180

81116 NFNADLTHAARWWVSFLIVGVLVLISILGENVVTKAMSFLVFPFIIFLFIFSLLLIPQWN 180

ICDCCJ07001 NFNADLTHAARWWVSFLIVGVLVLISILGENVVTKAMSFLVFPFIIFLFIFSLLLIPQWN 180

M1 NFNADLTHAARWWVSFLIVGVLVLISILGENVVTKAMSFLVFPFIIFLFIFSLLLIPQWN 180

327 NFNADLTHAARWWVSFLIVGVLVLISILGENVVTKAMSFLVFPFIIFLFIFSLLLIPQWN 180

1336 NFNADLTHAARWWVSFLIVGVLVLISILGENVVTKAMSFLVFPFIIFLFIFSLLLIPQWN 180

414 NFNADLIYAARWWIGFLIVGVLILISILGENVVTKAMSFLVFPFIIFLFIFSLLLIPQWN 180

****** :*****:.*******:*************************************

RM1221 SSLFANVDFSVISTSNFWVTLWLVIPVMVFSFNHSPIISSLACYCKKEYGDYAEPRARKI 240

S3 SSLFANVDFSVISTSNFWVTLWLVIPVMVFSFNHSPIISSLACYCKKEYGDYAEPRARKI 240

CF93-6 LSLFANVDFSVISTSNFWVTLWLVIPVMVFSFNHSPIISSLACYCKKEYGGYAEPRARKI 240

84-25 LSLFANVDFSVISTSNFWVTLWLVIPVMVFSFNHSPIISSLACYCKKEYGGYAEPRARKI 240

CG8486 LSLFANVDFSVISTSNFWVTLWLVIPVMVFSFNHSPIISSLACYCKKEYGGYAEPRARKI 240

NCTC 11168 LSLFANVDFSVISTSNFWVTLWLVIPVMVFSFNHSPIISSLACYCKKEYGGYAEPRARKI 240

IA3902 LSLFANVDFSVISTSNFWVTLWLVIPVMVFSFNHSPIISSLACYCKKEYGGYAEPRARKI 240

DFVF1099 LSLFANVDFSVISTSNFWVTLWLVIPVMVFSFNHSPIISSLACYCKKEYGGYAEPRARKI 240

305 LSLFANVDFSVISTSNFWVTLWLVIPVMVFSFNHSPIISSLACYCKKEYGGYAEPRARKI 240

CG8421 LSLFANVDFSVISTSNFWVTLWLVIPVMVFSFNHSPIISSLACYCKKEYGGYAEPRARKI 240

**ATCC 33251** SSLFTNVDFSVISTSNFWVTLWLVIPVMVFSFNHSPIISSLACYCKKEYGGYAEPRARKI 240

260.94 SSLFTNVDFSVISTSNFWVTLWLVIPVMVFSFNHSPIISSLACYCKKEYGDYAEPRARKI 240

HB93-13 SSLFTNVDFSVISTSNFWVTLWLVIPVMVFSFNHSPIISSLACYCKKEYGDYAEPRARKI 240

81-176 SSLFTNVDFSVISTSNFWVTLWLVIPVMVFSFNHSPIISSLACYCKKEYGDYAEPRARKI 240

81116 SSLFTNVDFSVISTSNFWVTLWLVIPVMVFSFNHSPIISSLACYCKKEYGDYAEPRARKI 240

ICDCCJ07001 SSLFTNVDFSVISTSNFWVTLWLVIPVMVFSFNHSPIISSLACYCKKEYGDYAEPRARKI 240

M1 SSLFTNVDFSVISTSNFWVTLWLVIPVMVFSFNHSPIISSLACYCKKEYGDYAEPRARKI 240

327 SSLFTNVDFSVISTSNFWVTLWLVIPVMVFSFNHSPIISSLACYCKKEYGDYAEPRARKI 240

1336 SSLFTNVDFSVISTSNFWVTLWLVIPVMVFSFNHSPIISSLACYCKKEYGDYAEPRARKI 240

414 SSLFTNVDLSIISTSNFWVTLWLVIPVMVFSFNHSPIISSLACYCKKEYGDYAEPRARKI 240

***:***:*:***************************************.*********

RM1221 ISLAIILMVFVVMFFVFSCALTFTPEDFASAKDQNINILTFIANKFPEVSLLAYVGPIVA 300

S3 ISLAIILMVFVVMFFVFSCALTFTPEDFASAKDQNINILTFIANKFPEVSLLAYVGPIVA 300

CF93-6 ISLAIILMVFVVMFFVFSCALTFTPEDFASAKDQNINILTFIANKFPEVSLLAYVGPIVA 300

84-25 ISLAIILMVFVVMFFVFSCALTFTPEDFASAKDQNINILTFIANKFPEVSLLAYVGPIVA 300

CG8486 ISLAIILMVFVVMFFVFSCALTFTPEDFASAKDQNINILTFIANKFPEVSLLAYVGPIVA 300

NCTC 11168 ISLAIILMVFVVMFFVFSCALTFTPEDFASAKDQNINILTFIANKFPEVSLLAYVGPIVA 300

IA3902 ISLAIILMVFVVMFFVFSCALTFTPEDFASAKDQNINILTFIANKFPEVSLLAYVGPIVA 300

DFVF1099 ISLAIILMVFVVMFFVFSCALTFTPEDFASAKDQNINILTFIANKFPEVSLLAYVGPIVA 300

305 ISLAIILMVFVVMFFVFSCALTFTPEDFASAKDQNINILTFIANKFPEVSLLAYVGPIVA 300

CG8421 ISLAIILMVFVVMFFVFSCALTFTPEDFASAKDQNINILTFIANKFPEVSLLAYVGPIVA 300

**ATCC 33251** ISLAIILMVFVVMFFVFSCALTFTPEDFASAKDQNINILTFIANKFPEVSLLAYVGPIVA 300

260.94 ISLAIILMVFVVMFFVFSCALTFTPEDFASAKDQNINILTFIANKFPEVSLLAYVGPIVA 300

HB93-13 ISLAIILMVFVVMFFVFSCALTFTPEDFASAKDQNINILTFIANKFPEVSLLAYVGPIVA 300

81-176 ISLAIILMVFVVMFFVFSCALTFTPEDFASAKDQNINILTFIANKFPEVSLLAYVGPIVA 300

81116 ISLAIILMVFVVMFFVFSCALTFTPEDFASAKDQNINILTFIANKFPEVSLLAYVGPIVA 300

ICDCCJ07001 ISLAIILMVFVVMFFVFSCALTFTPEDFASAKDQNINILTFIANKFPEVSLLAYVGPIVA 300

M1 ISLAIILMVFVVMFFVFSCALTFTPEDFASAKDQNINILTFIANKFPEVSLLAYVGPIVA 300

327 ISLAIILMVFVVMFFVFSCALTFTPEDFASAKDQNINILTFIANKFPEVSLLAYVGPIVA 300

1336 ISLAIILMVFVVMFFVFSCALTFTPEDFASAKDQNINILTFIANKFPEVSLLTYVGPIVA 300

414 ISLAVVLMVFVVMFFVFSCALTFTPEDFTSAKDQNVNILTFIANKFPEVSLLAYVGPIVA 300

****::**********************:******:****************:*******

RM1221 LVAISKSFLGHYLGSQEGLNGILYKASNGRIQGKFAQTLTAIITFAIAWLVAYKNPSVIG 360

S3 LVAISKSFLGHYLGSQEGLNGILYKASNGRIQGKFAQTLTAIITFAIAWLVAYKNPSVIG 360

CF93-6 LVAISKSFLGHYLGSQEGLNGILYKASNGRIQGKFAQTLTAIITFAIAWLVAYKNPSVIG 360

84-25 LVAISKSFLGHYLGSQEGLNGILYKASNGRIQGKFAQTLTAIITFAIAWLVAYKNPSVIG 360

CG8486 LVAISKSFLGHYLGSQEGLNGILYKASNGRIQGKFAQTLTAIITFAIAWLVAYKNPSVIG 360

NCTC 11168 LVAISKSFLGHYLGSQEGLNGILYKASNGRIQGKFAQTLTAIITFAIAWLVAYKNPSVIG 360

IA3902 LVAISKSFLGHYLGSQEGLNGILYKASNGRIQGKFAQTLTAIITFAIAWLVAYKNPSVIG 360

DFVF1099 LVAISKSFLGHYLGSQEGLNGILYKASNGRIQGKFAQTLTAIITFAIAWLVAYKNPSVIG 360

305 LVAISKSFLGHYLGSQEGLNGILYKASNGRIQGKFAQTLTAIITFAIAWLVAYKNPSVIG 360

CG8421 LVAISKSFLGHYLGSQEGLNGILYKASNGRIQGKFAQTLTAIITFAIAWFVAYKNPSVIG 360

**ATCC 33251** LVAISKSFLGHYLGSQEGLNGILYKASNGRIQGKFAQTLTAIITFAIAWLVAYKNPSVIG 360

260.94 LVAISKSFLGHYLGSQEGLNGILYKASNGKIQGKFAQTLTAIITFAIAWLVAYKNPSVIG 360

HB93-13 LVAISKSFLGHYLGSQEGLNGILYKASNGKIQGKFAQTLTAIITFAIAWLVAYKNPSVIG 360

81-176 LVAISKSFLGHYLGSQEGLNGILYKASNGKIQGKFAQTLTAIITFAIAWLVAYKNPSVIG 360

81116 LVAISKSFLGHYLGSQEGLNGILYKASNGKIQGKFAQTLTAIITFAIAWLVAYKNPSVIG 360

ICDCCJ07001 LVAISKSFLGHYLGSQEGLNGILYKASNGKIQGKFAQTLTAIITFAIAWLVAYKNPSVIG 360

M1 LVAISKSFLGHYLGSQEGLNGILYKASNGKIQGKFAQTLTAIITFAIAWLVAYKNPSVIG 360

327 LVAISKSFLGHYLGSQEGLNGILYKASNGKIQGKFAQTLTAIITFAIAWLVAYKNPSVIG 360

1336 LVAISKSFLGHYLGSQEGLNGILYKASNGKIQGKFAQTLTAIITFVIAWLVAYKNPSVIG 360

414 LVAISKSFLGHYLGSQEGLNGILYKASNGKIQGKLAQTLTAVITFAIAWFVAYKNPSVIG 360

*****************************:****:******:***.***:**********

RM1221 IIEAIGGPVLAILLFLMPLYCIYRFDILARFRNKFLDLFILVMGIVAISAAIHDLL 416

S3 IIEAIGGPVLAILLFLMPLYCIYRFDILARFRNKFLDLFILVMGIVAISAAIHDLL 416

CF93-6 IIEAIGGPVLAILLFLMPLYCIYRFDILARFRNKFLDLFVLVMGIVAISAAIHDLL 416

84-25 IIEAIGGPVLAILLFLMPLYCIYRFDILARFRNKFLDLFVLVMGIVAISAAIHDLL 416

CG8486 IIEAIGGPVLAILLFLMPLYCIYRFDILARFRNKFLDLFVLVMGIVAISAAIHDLL 416

NCTC 11168 IIEAIGGPVLAILLFLMPLYCIYRFDILARFRNKFLDLFVLVMGIVAISAAIHDLL 416

IA3902 IIEAIGGPVLAILLFLMPLYCIYRFDILARFRNKFLDLFVLVMGIVAISAAIHDLL 416

DFVF1099 IIEAIGGPVLAILLFLMPLYCIYRFDILARFRNKFLDLFVLVMGIVAISAAIHDLL 416

305 IIEAIGGPVLAILLFLMPLYCIYRFDILARFRNKFLDLFVLVMGIVAISAAIHDLL 416

CG8421 IIEAIGGPVLAILLFLMPLYCIYRFDILARFRNKFLDLFVLVMGIVAISAAIHDLL 416

**ATCC 33251** IIEAIGGPVLAILLFLMPLYCIYRFDILARFRNKFLDLFVLVMGIVAISAAIHDLL 416

260.94 IIEAIGGPVLAILLFLMPLYCIYRFDILARFRNKFLDLFILVMGIVAISAAIHDLL 416

HB93-13 IIEAIGGPVLAILLFLMPLYCIYRFDILARFRNKFLDLFILVMGIVAISAAIHDLL 416

81-176 IIEAIGGPVLAILLFLMPLYCIYRFDILARFRNKFLDLFILVMGIVAISAAIHDLL 416

81116 IIEAIGGPVLAILLFLMPLYCIYRFDILARFRNKFLDLFILVMGIVAISAAIHDLL 416

ICDCCJ07001 IIEAIGGPVLAILLFLMPLYCIYRFDILARFRNKFLDLFILVMGIVAISAAIHDLL 416

M1 IIEAIGGPVLAILLFLMPLYCIYRFDILARFRNKFLDLFILVMGIVAISAAIHDLL 416

327 IIEAIGGPVLAILLFLMPLYCIYRFDILARFRNKFLDLFILVMGIVAISAAIHDLL 416

1336 IIEAIGGPVLAILLFLMPLYCIYRFDILARFRNKFLDLFILVMGIVAISAAIHNLL 416

414 IIEAIGGPVLAILLFLMPLYCIYRFDILAKFRNKFLDLFILVMGIVAISAAIHNLL 416

*****************************:*********:*************:**

**Figure S4. Comparison of SdaC serine transporter** **protein sequences in various *C. jejuni* isolates.**
