# Supplementary figures and images for: Contribution of Amino Acid Catabolism to the Tissue Specific Persistence of Campylobacter jejuni in a Murine Colonization Model
Source: PLoS One. 2012 Nov 30;7(11):e50699. doi: 10.1371/journal.pone.0050699 (PMC3511319; doi:10.1371/journal.pone.0050699)

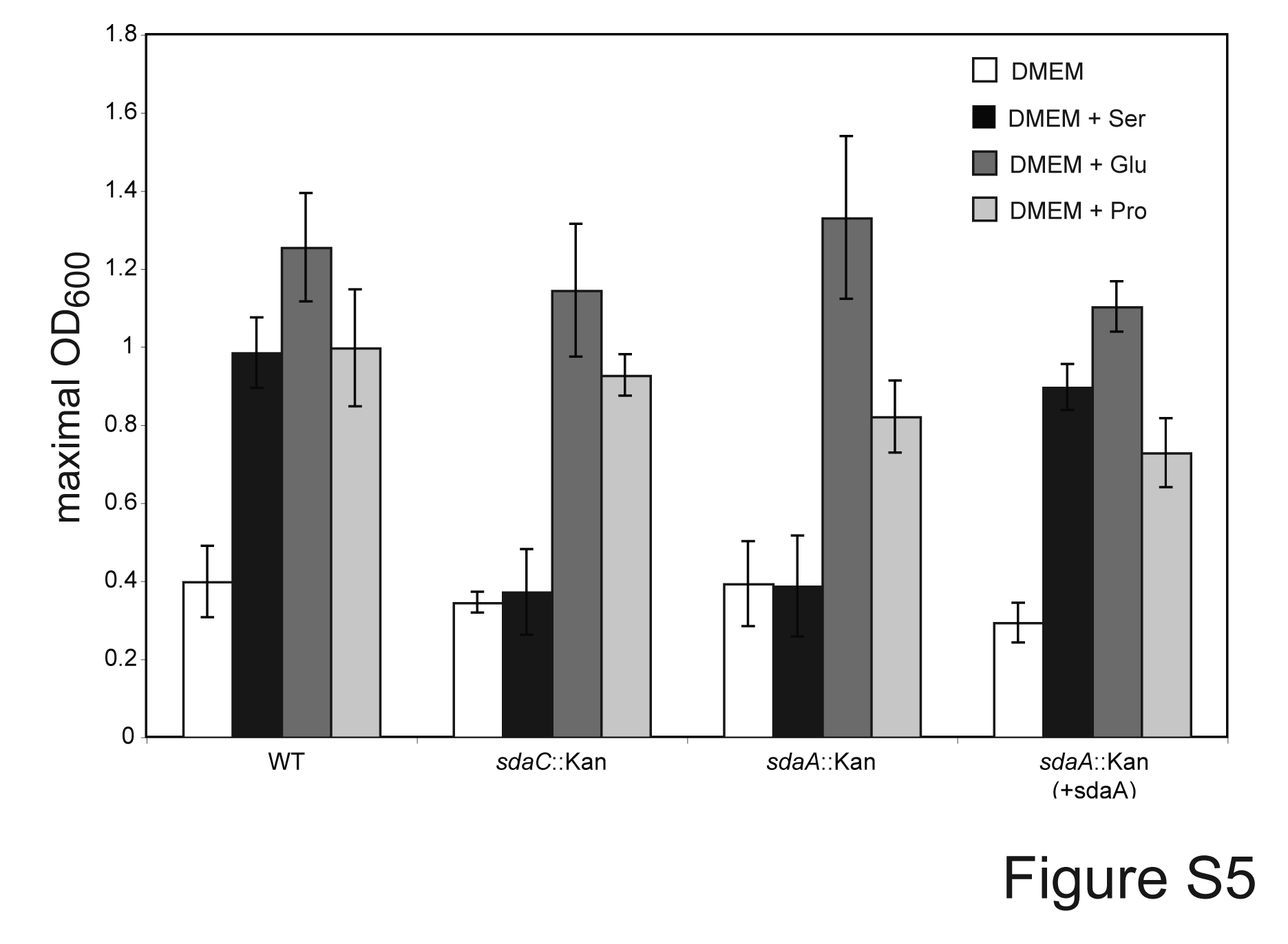

Supplement: Figure S5 — Growth of C. jejuni 81-176 and its isogenic sdaA and sdaC mutants. Growth characteristics of the C. jejuni 81-176 wild-type strain, its isogenic sdaC and sdaA mutants as well as a complemented sdaA mutant in DMEM and DMEM supplemented with 20 mM serine, glutamate or proline. The maximal optical densities (OD600) of liquid cultures from indicated C. jejuni strains over a time period of 24 hours are presented. (TIF) [file pone.0050699.s005.tif]

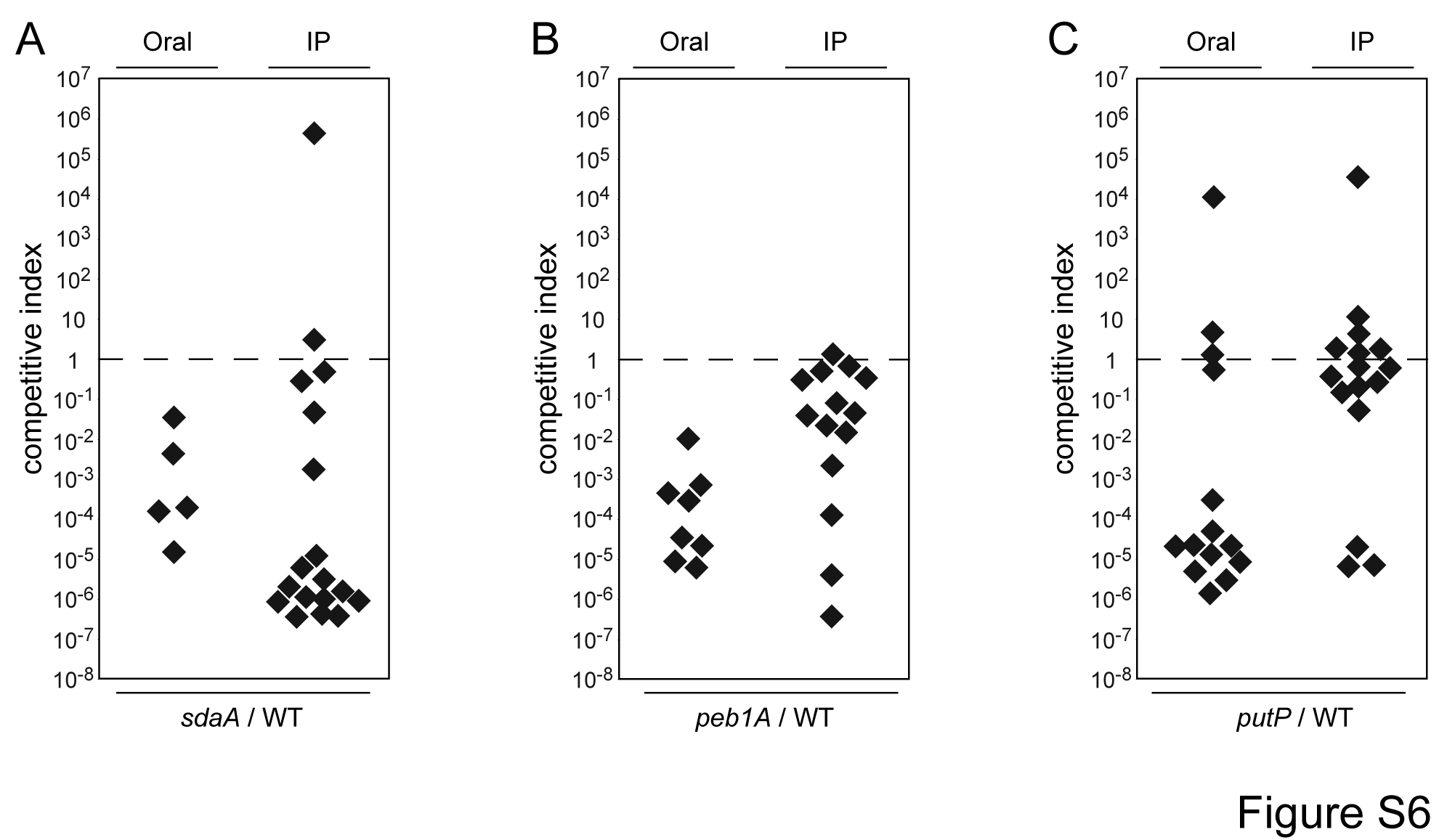

Supplement: Figure S6 — Competitive index of mice co-infection with C. jejuni 81-176 and its sdaA , peb1A or putP mutants. The competitive index, CI = (mutantoutput/wild-typeoutput)/(mutantinoculum/wild-typeinoculum), was calculated for each mouse infected with C. jejuni 81-176 wild-type strain and its indicated mutant. The output numbers representing the CFUs of wild-type and mutant strains recovered from the intestine or the liver of each animal are plotted in Figures 3, 4 and 6. Each mouse was infected with approximately the same number of wild-type and mutant strain as determined by the CFU counting of the inoculum. (TIF) [file pone.0050699.s006.tif]

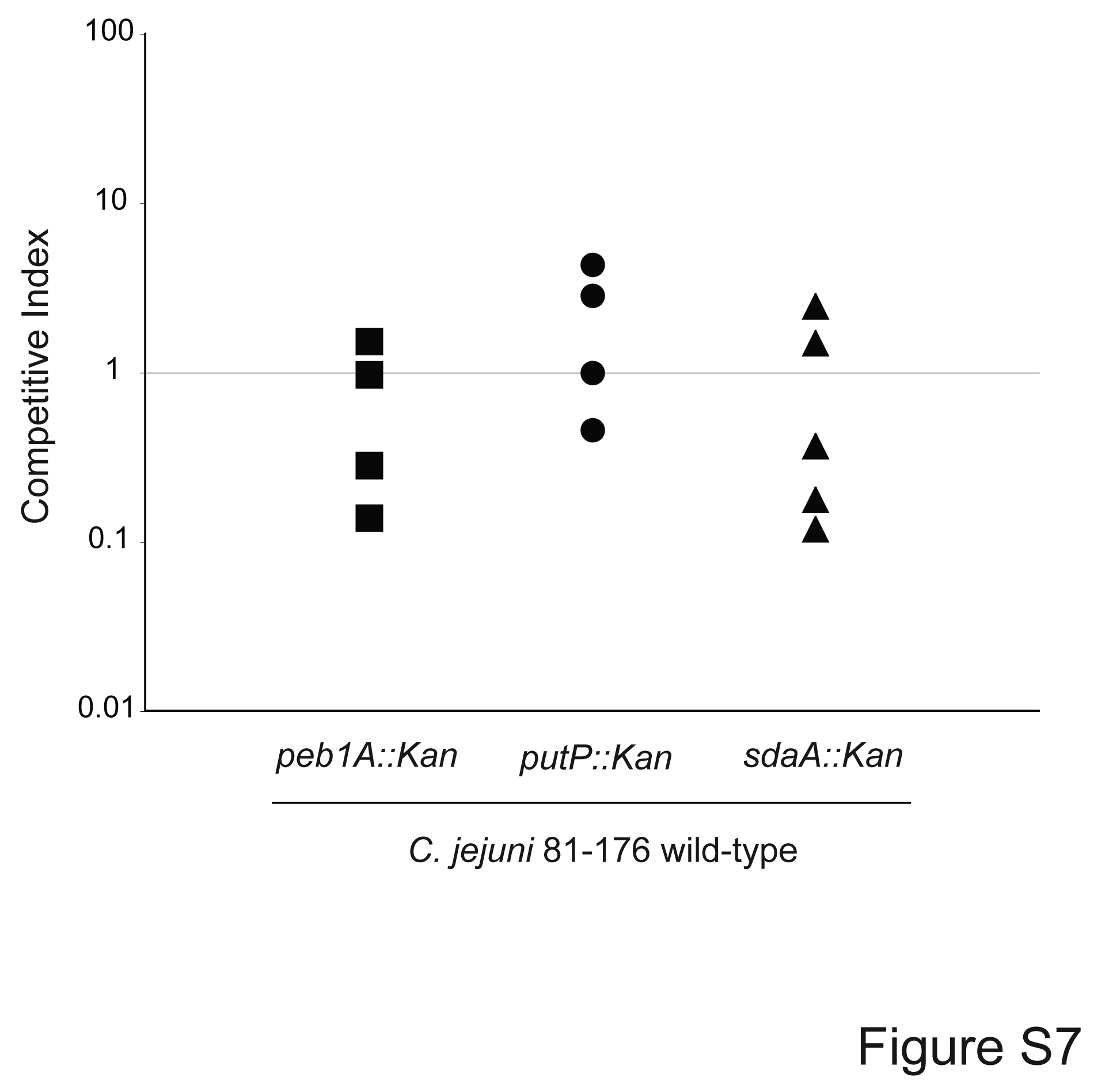

Supplement: Figure S7 — In vitro growth competition experiments of C. jejuni 81-176 and its mutants. Shown are the co-cultivation experiments of C. jejuni 81-176 with its peb1A, putP and sdaA mutants, respectively. Equal amounts of wild-type and a mutant strain were incubated in nutrient rich BHI medium over night and the CFUs of each strain were determined after 20 hours. Each symbol represents the calculated competitive index for one co-cultivation experiment. (TIF) [file pone.0050699.s007.tif]

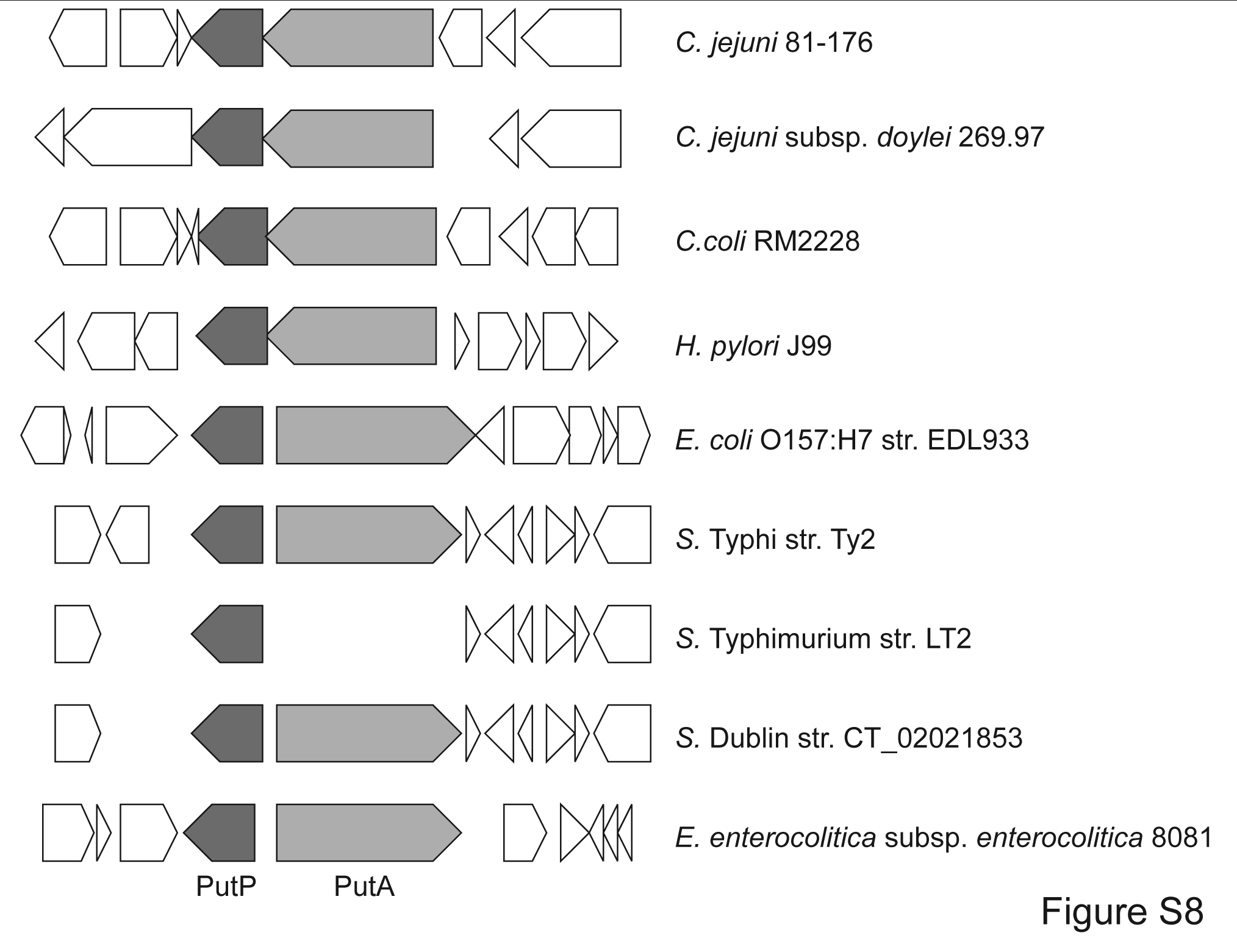

Supplement: Figure S8 — Comparison of the putA / putP gene cluster in Campylobacter and Enterobacteria . The schematic comparison of the putAputP gene locus and its flanking regions of several Campylobacter and Enterobacter strains was derived from the comparative genome database xBASE2 (Chaudhuri RR et al. (2008) Nucleic Acids Res., D543-6) in particular CampyDB (www.xbase.ac.uk/campydb) and ColiDB (www.xbase.ac.uk/colibase). The putA and putP genes are marked in light and dark grey, respectively. The genes of the flaking regions are represented as white arrows. (TIF) [file pone.0050699.s008.tif]
