# Supplementary material for: Contribution of Amino Acid Catabolism to the Tissue Specific Persistence of Campylobacter jejuni in a Murine Colonization Model
Source: PLoS One. 2012 Nov 30;7(11):e50699. doi: 10.1371/journal.pone.0050699 (PMC3511319; doi:10.1371/journal.pone.0050699)
Supplement: Table S1 — Proteobacteria with homologues to the L-serine dehydratase SdaA of C. jejuni 81-176. The table shows the homology between the SdaA protein of C. jejuni 81-176 and the SdaA proteins in other proteobacteria with their given accession numbers. The percent of amino acids identical and similar (conserved amino acid exchanges) between the serine ammonia-lyase of C. jejuni 81-176 and the other SdaA proteins were determined by BLASTP analysis (http://blast.ncbi.nlm.nih.gov/Blast.cgi). The order of the table reflects the score values calculated by the BLASTP algorithm. C. jejuni isolates are marked in red, other Campylobacter species in orange and Helicobacter species in yellow. Only a subset of C. jejuni isolates are listed, but all sequenced C. jejuni strains encode for SdaA homologues that are 100% or 99% identical to the SdaA protein of C. jejuni 81-176. (DOC) [file pone.0050699.s009.doc]

**Table S1. Proteobacteria with homologues to the serine ammonia-lyase SdaA of *C. jejuni* 81-176.**

| **Proteobacteria encoding SdaA** | **Acc. number** | **Identities (%)** | **Positives (%)** |
| --- | --- | --- | --- |
| *Campylobacter jejuni* 81-176 | ZP_02271920 | 100 | 100 |
| *Campylobacter jejuni* 81116 | YP_001483100 | 100 | 100 |
| *Campylobacter jejuni* RM 1221 | YP_179767 | 99 | 99 |
| *Campylobacter jejuni* NCTC11168 | YP_002344993 | 99 | 99 |
| *Campylobacter jejuni* subsp. *doylei* 269.97 | YP_001398903 | 96 | 98 |
| *Campylobacter coli* RM2228 | ZP_00367957 | 96 | 98 |
| *Campylobacter lari* RM2100 | YP_002574702 | 76 | 89 |
| *Helicobacter bilis* ATCC 43879 | ZP_04581007 | 72 | 86 |
| *Succinatimonas hippei* YIT 12066 | ZP_08078167 | 67 | 81 |
| *Helicobacter canadensis* MIT 98-5491 | ZP_04871063 | 67 | 80 |
| *Helicobacter cinaedi* CCUG 18818 | ZP_07806022 | 66 | 81 |
| *Helicobacter pullorum* MIT 98-5489 | ZP_04809370 | 64 | 79 |
| *Helicobacter pylori* 26695 | NP_206932 | 54 | 68 |
| *Helicobacter acinonychis str.* Sheeba | YP_664153 | 53 | 68 |
| *Helicobacter suis* HS1 | ZP_08052829 | 48 | 68 |
| *Helicobacter felis* ATCC 49179 | YP_004073089 | 50 | 67 |
| *Helicobacter mustelae* 12198 | YP_003516276 | 48 | 65 |
| *Vibrio vulnificus* YJ016 | NP_936552 | 49 | 66 |
| *Eikenella corrodens* ATCC 23834 | ZP_03713662 | 47 | 63 |
| *Vibrio splendidus* ATCC 33789 | EGU37989 | 48 | 65 |
| *Psychromonas sp.* CNPT3 | ZP_01216641 | 49 | 65 |
| *Vibrio cholerae* TMA 21 | ZP_04403583 | 48 | 65 |
| *Vibrio parahaemolyticus* RIMD 2210633 | NP_799764 | 49 | 65 |
| *Photobacterium profundum* SS9 | YP_131992 | 47 | 65 |
| *Sutterella wadsworthensis* 3_1_45B | ZP_08015780 | 48 | 64 |
| *Aliivibrio salmonicida* LFI1238 | YP_002265349 | 48 | 64 |
| *Haemophilus influenzae* 7P49H1 | ZP_04466483 | 46 | 64 |
| *Aggregatibacter aphrophilus* F0387 | EHB89863 | 47 | 65 |
| Haemophilus haemolyticus M19107 | EGT74613 | 46 | 64 |
| Gallibacterium anatis UMN179 | YP_004418969 | 45 | 64 |
| Mannheimia haemolytica serotype A2 str. BOVINE | ZP_05990193 | 46 | 64 |
| Pasteurella dagmatis ATCC 43325 | ZP_05919588 | 46 | 64 |
| Salmonella enterica subsp. enterica serovar Typhi str. CT18 | NP_456333 | 46 | 64 |
| Citrobacter koseri ATCC BAA-895 | YP_001452743 | 46 | 64 |
| Salmonella enterica subsp. enterica serovar Typhimurium str. LT2 | NP_460782 | 46 | 64 |

The table shows the homology between the SdaA protein of *C. jejuni* 81-176 and the SdaA proteins in other proteobacteria with their given accession numbers. The percent of amino acids identical and similar (conserved amino acid exchanges) between the serine ammonia-lyase of *C. jejuni* 81-176 and the other SdaA proteins were determined by BLASTP analysis (http://blast.ncbi.nlm.nih.gov/Blast.cgi). The order of the table reflects the score values calculated by the BLASTP algorithm. *C. jejuni* isolates are marked in red, other *Campylobacter* species in orange and *Helicobacter* species in yellow. Only a subset of *C. jejuni* isolates are listed, but all sequenced *C. jejuni* strains encode for SdaA homologues that are 100% or 99% identical to the SdaA protein of *C. jejuni* 81-176.
