# Supplementary material for: Contribution of Amino Acid Catabolism to the Tissue Specific Persistence of Campylobacter jejuni in a Murine Colonization Model
Source: PLoS One. 2012 Nov 30;7(11):e50699. doi: 10.1371/journal.pone.0050699 (PMC3511319; doi:10.1371/journal.pone.0050699)
Supplement: Table S2 — Proteobacteria with homologues to the serine transporter SdaC of C. jejuni 81-176. The table shows the homology between the SdaC protein of C. jejuni 81-176 and the SdaC proteins in other proteobacteria. The order represents the grade of homology according to the score calculated by the BlastP algorithm (http://blast.ncbi.nlm.nih.gov/Blast.cgi). C. jejuni isolates are marked in red other Campylobacter species in orange and Helicobacter species in yellow. Sequenced C. jejuni strains that are not represented in the table encode for SdaC homologues that are at least 99% identical to the SdaC of C. jejuni 81-176. (DOC) [file pone.0050699.s010.doc]

**Table S2. Proteobacteria with homologues to the serine transporter SdaC of *C. jejuni* 81-176.**

| **Proteobacteria encoding SdaC** | **Acc. number** | **Identities (%)** | **Positives (%)** |
| --- | --- | --- | --- |
| *Campylobacter jejuni* 81-176 | ZP_02271921 | 100 | 100 |
| *Campylobacter jejuni* 81116 | YP_001483101 | 100 | 100 |
| *Campylobacter jejuni* RM 1221 | YP_179768 | 99 | 99 |
| *Campylobacter jejuni* NCTC11168 | YP_002344994 | 99 | 99 |
| Helicobacter bilis ATCC 43879 | ZP_04581008 | 70 | 82 |
| Helicobacter felis ATCC 49179 | YP_004074223 | 54 | 73 |
| Helicobacter mustelae 12198 | YP_003516275 | 54 | 72 |
| *Proteus mirabilis* HI4320 | YP_002150439 | 51 | 70 |
| *Erwinia billingiae* Eb661 | YP_003740839 | 50 | 71 |
| *Escherichia albertii* TW07627 | ZP_02900365 | 50 | 71 |
| *Enterobacter aerogenes* KCTC 2190 | YP_004590621 | 50 | 70 |
| *Yersinia enterocolitica subsp. enterocolitica* 8081 | YP_001005780 | 50 | 71 |
| *Yersinia pseudotuberculosis* IP 32953 | YP_069884 | 49 | 70 |
| *Yersinia pestis* KIM 10 | NP_670162 | 49 | 70 |
| *Salmonella enterica subsp. enterica serovar* Typhi str. CT18 | NP_457365 | 51 | 70 |
| *Salmonella enterica subsp. enterica serovar* Typhimurium str. LT2 | NP_461890 | 51 | 70 |
| *Photobacterium leiognathi subsp. mandapamensis* svers.1.1. | ZP_08309497 | 50 | 70 |
| *Citrobacter youngae* ATCC 29220 | ZP_06355175 | 51 | 71 |
| *Klebsiella pneumoniae* 342 | YP_002236852 | 50 | 70 |
| *Psychromonas sp.* CNPT3 | ZP_01216641 | 49 | 65 |
| *Vibrio cholerae* TMA 21 | ZP_04403583 | 48 | 65 |
| *Vibrio parahaemolyticus* RIMD 2210633 | NP_799764 | 49 | 65 |
| *Photobacterium profundum* SS9 | YP_131992 | 47 | 65 |
| *Sutterella wadsworthensis* 3_1_45B | ZP_08015780 | 48 | 64 |
| *Aliivibrio salmonicida* LFI1238 | YP_002265349 | 48 | 64 |
| *Haemophilus influenzae* 7P49H1 | ZP_04466483 | 46 | 64 |
| *Aggregatibacter aphrophilus* F0387 | EHB89863 | 47 | 65 |
| *Haemophilus haemolyticus* M19107 | EGT74613 | 46 | 64 |
| *Gallibacterium anatis* UMN179 | YP_004418969 | 45 | 64 |
| *Helicobacter pullorum* MIT 98-5489 | ZP_04809369 | 43 | 62 |
| *Campylobacter jejuni subsp. doylei* 269.97 | YP_001398904 | 41 | 60 |
| *Helicobacter canadensis* MIT 98-5491 | ZP_04871062 | 43 | 61 |
| *Campylobacter coli* JV20 | ZP_00367956 | 41 | 60 |
| *Campylobacter coli* RM2228 | ZP_07401597 | 41 | 60 |
| *Campylobacter lari* RM2100 | YP_002574701 | 40 | 60 |
| *Campylobacter fetus subsp. fetus* 82-40 | YP_891624 | 40 | 60 |
| *Helicobacter cinaedi* CCUG 18818 | ZP_07805820 | 40 | 57 |
| *Campylobacter upsaliensis* JV21 | ZP_07892998 | 40 | 59 |
| *Campylobacter upsaliensis RM3195* | ZP_00371633 | 40 | 58 |
| *Campylobacter hominis ATCC BAA-381* | YP_001406444 | 39 | 62 |
| *Helicobacter hepaticus* ATCC 51449 | NP_860631 | 38 | 55 |
| *Helicobacter felis* ATCC 49179 | YP_004073088 | 41 | 61 |
| *Helicobacter pylori* 26695 | AAD07203 | 41 | 60 |
| *Helicobacter acinonychis str.* Sheeba | YP_664154 | 42 | 61 |
| *Helicobacter suis* HS1 | ZP_08052830 | 39 | 61 |

The table shows the homology between the SdaC protein of *C. jejuni* 81-176 and the SdaC proteins in other proteobacteria. The order represents the grade of homology according to the score calculated by the BlastP algorithm (http://blast.ncbi.nlm.nih.gov/Blast.cgi). *C. jejuni* isolates are marked in red other *Campylobacter* species in orange and *Helicobacter* species in yellow. Sequenced *C. jejuni* strains that are not represented in the table encode for SdaC homologues that are at least 99% identical to the SdaC of *C. jejuni* 81-176.
