# Supplementary material for: Contribution of Amino Acid Catabolism to the Tissue Specific Persistence of Campylobacter jejuni in a Murine Colonization Model
Source: PLoS One. 2012 Nov 30;7(11):e50699. doi: 10.1371/journal.pone.0050699 (PMC3511319; doi:10.1371/journal.pone.0050699)
Supplement: Table S3 — Proteobacteria with homologues to the proline dehydrogenase/delta 1-pyrroline-5-carboxylate dehydrogenase PutA of C. jejuni 81-176. The table shows the homology between the PutA protein of C. jejuni 81-176 and the PutA proteins in other proteobacteria with their given accession numbers. The percent of amino acids identical and similar (conserved amino acid exchanges) between C. jejuni 81-176 PutA and the PutA proteins of other presented proteobacteria were determined by BLASTP analysis (http://blast.ncbi.nlm.nih.gov/Blast.cgi). The order of the table reflects the score values calculated by the BLASTP algorithm. C. jejuni isolates are marked in red, other Campylobacter species in orange and Helicobacter species in yellow. Only a subset of C. jejuni isolates are listed, but all sequenced C. jejuni strains encode for SdaA homologues that are at least 99% identical to the SdaA protein of C. jejuni 81-176. (DOC) [file pone.0050699.s011.doc]

**Table S3. Proteobacteria with homologues to the proline dehydrogenase / delta 1-pyrroline-5-carboxylate dehydrogenase PutA of *C. jejuni* 81-176.**

| **Proteobacteria encoding PutA** | **Acc. number** | **Identities (%)** | **Positives (%)** |
| --- | --- | --- | --- |
| *C. jejuni* 81-176 | ZP_02271801 | 100 | 100 |
| *C. jejuni* NCTC11168 | YP_002344882 | 99 | 99 |
| *C. jejuni* RM 1221 | YP_179657 | 99 | 99 |
| *C. jejuni* 81116 | YP_001482981 | 99 | 99 |
| *C. jejuni* subsp. doylei 269.97 | YP_001398805 | 97 | 99 |
| *C. coli* RM2228 | ZP_00368214 | 85 | 93 |
| *C. coli* JV20 | ZP_07401459 | 85 | 93 |
| *C. lari* RM2100 | YP_002574973 | 82 | 91 |
| *H. canadensis* MIT 98-5491 | ZP_04870753 | 79 | 89 |
| *C. upsaliensis* JV21 | ZP_07894422 | 78 | 88 |
| *C. upsaliensis* RM3195 | ZP_00371223 | 79 | 88 |
| *H. hepaticus* ATCC 51449 | NP_859687 | 71 | 84 |
| *C. fetus subsp. fetus* 82-40 | YP_892398 | 66 | 82 |
| *H. mustelae* 12198 | YP_003516223 | 66 | 81 |
| *H. pylori* 26695 | NP_206857 | 65 | 79 |
| *H. felis* ATCC 49179 | YP_004073925 | 61 | 75 |
| *H. bilis* ATCC 43879 | ZP_04580976 | 58 | 74 |
| *Arcobacter nitrofigilis* DSM 7299 | YP_003655956 | 48 | 67 |
| *Sulfurimonas denitrificans* DSM 1251 | YP_393840 | 46 | 66 |
| *Desulfatibacillum alkenivorans* AK-01 | YP_002433097 | 42 | 61 |
| *Desulfonatronospira thiodismutans* ASO3-1 | ZP_07015683 | 41 | 61 |
| *Verrucomicrobiae bacterium* DG1235 | ZP_05055749 | 41 | 60 |
| *Verrucomicrobium spinosum* DSM 4136 | ZP_02925945 | 39 | 59 |
| *Paludibacter propionicigenes* WB4 | YP_004042135 | 39 | 59 |
| *Dysgonomonas mossii* DSM 22836 | ZP_08469539 | 40 | 61 |
| *Parachlamydia acanthamoebae str.* Hall's coccus | ZP_06298641 | 38 | 57 |
| *H. suis* HS1 | ZP_08053889 | 59 | 76 |
| *Bacteroides vulgatus* ATCC 8482 | YP_001300839 | 38 | 58 |

The table shows the homology between the PutA protein of *C. jejuni* 81-176 and the PutA proteins in other proteobacteria with their given accession numbers. The percent of amino acids identical and similar (conserved amino acid exchanges) between *C. jejuni* 81-176 PutA and the PutA proteins of other presented proteobacteria were determined by BLASTP analysis (http://blast.ncbi.nlm.nih.gov/Blast.cgi). The order of the table reflects the score values calculated by the BLASTP algorithm. *C. jejuni* isolates are marked in red, other *Campylobacter* species in orange and *Helicobacter* species in yellow. Only a subset of *C. jejuni* isolates are listed, but all sequenced *C. jejuni* strains encode for SdaA homologues that are at least 99% identical to the SdaA protein of *C. jejuni* 81-176.
