# Supplementary material for: Contribution of Amino Acid Catabolism to the Tissue Specific Persistence of Campylobacter jejuni in a Murine Colonization Model
Source: PLoS One. 2012 Nov 30;7(11):e50699. doi: 10.1371/journal.pone.0050699 (PMC3511319; doi:10.1371/journal.pone.0050699)
Supplement: Table S4 — Proteobacteria with homologues to the proline transporter PutP of C. jejuni 81-176. The table illustrates the homology between the PutP protein of C. jejuni 81-176 and the PutP proteins in other proteobacteria. The accession numbers of the PutP proteins from indicated bacteria are listed. The order represents the grade of homology according to the score calculated by the BlastP algorithm (http://blast.ncbi.nlm.nih.gov/Blast.cgi). C. jejuni isolates are marked in red, Campyobacter species besides C. jejuni are highlighted in orange and Helicobacter species in yellow. Not all C. jejuni isolates are listed, but every sequenced C. jejuni strain encodes for a PutP homologue that is at least 98% identical to the PutP protein of C. jejuni 81-176. (DOC) [file pone.0050699.s012.doc]

**Table S4. Proteobacteria with homologues to the proline transporter PutP of**

***C. jejuni* 81-176.**

| **Proteobacteria encoding PutP** | **Acc. number** | **Identities (%)** | **Positives (%)** |
| --- | --- | --- | --- |
| *C. jejuni* 81-176 | ZP_02271800 | 100 | 100 |
| *C. jejuni* NCTC11168 | YP_002344881 | 99 | 99 |
| *C. jejuni* RM 1221 | YP_179656 | 99 | 99 |
| *C. jejuni* 81116 | YP_001482980 | 99 | 99 |
| *C. jejuni* subsp. doylei 269.97 | YP_001398804 | 98 | 99 |
| *C. coli* RM2228 | ZP_00368213 | 84 | 92 |
| *C. coli* JV20 | ZP_07401458 | 84 | 92 |
| *C. lari* RM2100 | YP_002574974 | 82 | 90 |
| *C. upsaliensis* JV21 | ZP_07894423 | 80 | 91 |
| *C. upsaliensis* RM3195 | ZP_00371224 | 80 | 91 |
| *C. fetus subsp. fetus* 82-40 | YP_892399 | 77 | 88 |
| *H. pylori* 26695 | NP_206856 | 74 | 88 |
| *H. acinonychis* *str.* Sheeba | YP_665273 | 73 | 97 |
| *H. hepaticus* ATCC 51449 * | NP_859686 | 81 | 91 |
| *H. suis* HS1 | ZP_08053888 | 70 | 86 |
| *H. felis* ATCC 49179 | YP_004073924 | 69 | 84 |
| *W. succinogenes* DSM 1740 | NP_907055 | 69 | 84 |
| *C. lari* RM2100 | YP_002575061 | 62 | 79 |
| *Bacillus selenitireducens* MLS10 | YP_003699092 | 54 | 73 |
| *Sulfurimonas denitrificans* DSM 1251 | YP_393839 | 53 | 72 |
| *Enterobacter hormaechei* ATCC 49162 | ZP_08496576 | 52 | 73 |
| *Enterobacter cloacae subsp. cloacae*  ATCC 13047 | YP_003613110 | 52 | 72 |
| *Desulfohalobium retbaense* DSM 5692 | YP_003198823 | 54 | 71 |
| *Pectobacterium carotovorum subsp. carotovorum* PC1 | YP_003019569 | 54 | 71 |
| *Providencia alcalifaciens* DSM 30120 | ZP_03317592 | 53 | 70 |
| *Pseudomonas aeruginosa* PAO1 | NP_249474 | 52 | 68 |
| *Geobacillus thermoleovorans* CCB_US3_UF5 | AEV21164 | 52 | 71 |
| *Pseudomonas stutzeri ATCC* 17588 | YP_004713526 | 54 | 71 |

The table illustrates the homology between the PutP protein of *C. jejuni* 81-176 and the PutP proteins in other proteobacteria. The accession numbers of the PutP proteins from indicated bacteria are listed. The order represents the grade of homology according to the score calculated by the BlastP algorithm (http://blast.ncbi.nlm.nih.gov/Blast.cgi). *C. jejuni* isolates are marked in red, *Campyobacter* species besides *C. jejuni* are highlighted in orange and *Helicobacter* species in yellow. Not all *C. jejuni* isolates are listed, but every sequenced *C. jejuni* strain encodes for a PutP homologue that is at least 98% identical to the PutP protein of *C. jejuni* 81-176.
