# Supplementary material for: Contribution of Amino Acid Catabolism to the Tissue Specific Persistence of Campylobacter jejuni in a Murine Colonization Model
Source: PLoS One. 2012 Nov 30;7(11):e50699. doi: 10.1371/journal.pone.0050699 (PMC3511319; doi:10.1371/journal.pone.0050699)
Supplement: Table S5 — L-serine dehydratase activity in cell extracts of C. jejuni isolates. The mean serine dehydratase activities with standard deviations are shown for measurements repeated four (a) and three (b) times in triplicates. The statistical significance in the differences of SdaA activity between C. jejuni 81-176 and C. jejuni 33251 or RM1221 was calculated by Student t test: * P<0.01; ** P<0.05. (DOC) [file pone.0050699.s013.doc]

**Table S5. Serine dehydratase activity in cell extracts of *C. jejuni* isolates**

| *C. jejuni*  wild-type strain | L-serine dehydratase activity  [nmol/(min*mg of protein )] |
| --- | --- |
| 81-176 | 78.4 +/- 5.8 ^a^ |
| 33251 | 63.6 +/- 4.7 ^b^ * |
| RM1221 | 61.8 +/- 12 ^b^ ** |

The mean serine dehydratase acitvities with standard deviations are shown for measurements repeated four (a) and three (b) times in triplicates. The statistical significance in the differences of SdaA activity between *C. jejuni* 81-176 and *C. jejuni* 33251 or RM1221 was calculated by Student *t* test: * P < 0.01; ** P < 0.05
