# Supplementary material for: Contribution of Amino Acid Catabolism to the Tissue Specific Persistence of Campylobacter jejuni in a Murine Colonization Model
Source: PLoS One. 2012 Nov 30;7(11):e50699. doi: 10.1371/journal.pone.0050699 (PMC3511319; doi:10.1371/journal.pone.0050699)
Supplement: Table S6 — Primers used in this study. (DOC) [file pone.0050699.s014.doc]

**Table S6. Primers used in this study.**

| Name | Sequence 5’ - 3’ |
| --- | --- |
| DHO2_*aphA*3-3’ | CGCGGATCC**AAGCTT**TTTAGACATCTAAAT |
| DHO51_*aphA*3-5’ | GATCCTAGG**AAGCTT**GAATTCTCATGTTTGACAGCTTATCATC |
| DHO231_putA-5’ | AGCTATGGT**TCTAGA**TGCAAATCCTAGT |
| DHO232_putA-3’ | CCG**CTCGAG**CTCTAATGCCTAAATTTCCATAACGA |
| DHO233_putP-5’ | GC**TCTAGA**TACCCCAATAGCAGTGATGTTTGT |
| DHO234_putP-3’ | CCGC**CTCGAG**ATCACCGCCGAAGCTACTAAAAACCCT |
| DHO283_sdaA-5’ | GC**TCTAGA**GTTTTGGTAATGGGGATAGTTGCA |
| DHO284_sdaA-3’ | CCG**CTCGAG**CACTTTAGCATACTGTTTTTAAATTTGTAGC |
| DHO285_sdaC-5’ | GC**TCTAGA**AAGGAAGAATAATGAATACTCCTAAATGGACTAG |
| DHO368_putP-SD | CATG**CCATGG**TTATGGAAATTTAGGCATTAGAGCAATATCT |
| DHO287_putP-3’ | GG**ACTAGT**GGCTTTTGTTCTCAAAGCTCTTTTAACATC |
| DHO349_erm-5’ | G**GAATTC**AGCTTTGGCTAACACACACGCCAT |
| DHO350_erm-3’ | G**GAATTC**TTACTTATTAAATAATTTATA |
| DHO352_sdaA | GATTTGCGTGTCATTGCCATTCTAGC |
| OW2_sdaA-SD-5’ | CATG**ACTAGT**CCATGGTTAAATTAAGGATAAAAAATGAGTAA  TTTAAGCATTTTTAAAATAGG |
| OW3_sdaA-3’ | G**ACTAGT**ACTTTAGCATACTGTTTTTAAATTTGTAGCAAGACCACC |
| OW10_sdaA | GAGGAAATTCGTGCTTATTGTCTTGA |
| sdaA_fwd | TAGT**GGATCC**TTTTATGCGAGAAATGATTTAAGAT |
| sdaA_rev | GTTT**CTGCAG**TAATGTTTTTTGTTTTTTCTTGTGCT |
| SR15_recA | ATCAACATGTAGGACTTCAAG |
| SR16_recA | CCTTCACGGCTTAAACCCTCTC |
