# Supplementary material for: Contribution of Amino Acid Catabolism to the Tissue Specific Persistence of Campylobacter jejuni in a Murine Colonization Model
Source: PLoS One. 2012 Nov 30;7(11):e50699. doi: 10.1371/journal.pone.0050699 (PMC3511319; doi:10.1371/journal.pone.0050699)
Supplement: Table S7 — Strains and plasmids used in this study. (DOC) [file pone.0050699.s015.doc]

**Table S7. Strains and plasmids used in this study.**

| Strain or plasmid | Description | Reference and/or source |
| --- | --- | --- |
| *E. coli* strains | | |
| DH5α | F^-^, ö80*lac*Z∆M15, ∆(*lac*ZYA-*arg*F)U169, *deo*R *rec*A1, *end*A1, *hsd*R17(rk-, mk+), *pho*A,  *sup*E44, *thi*-1, *gyr*A96, *rel*A1 ë^-^ | Stratagene |
| GM2199 | F-, dam-13::Tn9, thr-1, ara-14, leuB6, tonA31 lacY1, tsx-78, supE44, galK2, galT22, hisG4, rpsL136, xyl-5, mtl-1, thi-1 | [[1](#_ENREF_1)] |
| *C. jejuni* strains | | |
| 81-176 | wild-type; human isolate | [[2](#_ENREF_2),[3](#_ENREF_3)] |
| 81116 | wild-type; human isolate | [[4](#_ENREF_4)] |
| RM1221 | wild-type; isolate from the skin of chicken | [[5](#_ENREF_5)] |
| 305 | wild-type; poultry isolate (from turkey skin surface) | [[6](#_ENREF_6)] |
| 327 | wild-type; poultry isolate (from turkey skin surface) | [[7](#_ENREF_7)] |
| DFVF1099 | wild-type; poultry isolate (from chicken skin surface) | [[6](#_ENREF_6)] |
| CB25 | *peb1A::aphA3* (*cjj81176_0928::aphA3*) | [[8](#_ENREF_8)] |
| CB101 | *putA::aphA3* (*cjj81176_1495::aphA3*) | this study |
| CB102 | *putP::aphA3* (*cjj81176_1494::aphA3*) | this study |
| CB115 | complemented *putP* mutant (*cjj81176_1494::aphA3 putP*) | this study |
| CB103 | *sdaA::aphA3* (*cjj81176_1615::aphA3*) | this study |
| CH18 | *sdaC::erm* (*cjj81176_1616::erm*) | this study |
| CH16 | complemented *sdaA* mutant (*cjj81176_1615::aphA3* *sdaA*) | this study |
| CH19 | *katA::aphA3-cat* (*cjj81176_1387::aphA3-erm*) | this study |
| Plasmids | | |
| pBluescript II | cloning vector, Amp^R^ | Stratagene |
| pDHO36 | pBluescriptIIKS with erythromycin resistance cassette (*erm*) of plasmid pNE131 [[9](#_ENREF_9)] | this study |
| pILL600 | plasmid with kanamycin resistance cassette (*aphA3*) | [[10](#_ENREF_10)] |
| pRY109 | plasmid with subcloned chloramphenicol acetyltransferase gene (*cat* cassette) of *C. coli* | [[11](#_ENREF_11)] |
| pSB3021 | *Campylobacter* complementation vector, Cm^R^ | [[12](#_ENREF_12)] |
| pSB3030 | *putA* gene (*cjj81176_1495*) subcloned in pBluescript | this study |
| pSB3031 | *putA*(*cjj81176_1495*)*::aphA3* in pBluescript | this study |
| pSB3032 | *putP**(cjj81176_1494)::aphA3* in pBluescript | this study |
| pSB3033 | *putP(cjj81176_1494)* subcloned in pSB3021 | this study |
| pSB3622 | *sdaA*(*cjj81176_1615*)*::aphA3* in pBluescript | this study |
| pSK1 | *sdaC_sdaA*(*cjj81176_1616,cjj81176_1615*) in pBluescript | this study |
| pOW34 | *sdaC*(*cjj81176_1616)::erm_sdaA*(*cjj81176_1615*) in pBluescript | this study |
| pOW4 | *sdaA*(*cjj81176_1615*) subcloned in pSB3021 | this study |

1. Marinus MG, Carraway M, Frey AZ, Brown L, Arraj JA (1983) Insertion mutations in the dam gene of Escherichia coli K-12. Mol Gen Genet 192: 288-289.

2. Korlath JA, Osterholm MT, Judy LA, Forfang JC, Robinson RA (1985) A point-source outbreak of campylobacteriosis associated with consumption of raw milk. J Infect Dis 152: 592-596.

3. Hofreuter D, Tsai J, Watson RO, Novik V, Altman B, et al. (2006) Unique features of a highly pathogenic Campylobacter jejuni strain. Infect Immun 74: 4694-4707.

4. Pearson BM, Gaskin DJ, Segers RP, Wells JM, Nuijten PJ, et al. (2007) The complete genome sequence of Campylobacter jejuni strain 81116 (NCTC11828). J Bacteriol 189: 8402-8403.

5. Fouts DE, Mongodin EF, Mandrell RE, Miller WG, Rasko DA, et al. (2005) Major structural differences and novel potential virulence mechanisms from the genomes of multiple campylobacter species. PLoS Biol 3: e15.

6. Takamiya M, Ozen A, Rasmussen M, Alter T, Gilbert T, et al. (2011) Genome sequences of two stress-tolerant Campylobacter jejuni poultry strains, 305 and DFVF1099. J Bacteriol 193: 5546-5547.

7. Takamiya M, Ozen A, Rasmussen M, Alter T, Gilbert T, et al. Genome Sequence of Campylobacter jejuni strain 327, a strain isolated from a turkey slaughterhouse. Stand Genomic Sci 4: 113-122.

8. Novik V, Hofreuter D, Galan JE Identification of Campylobacter jejuni genes involved in its interaction with epithelial cells. Infect Immun 78: 3540-3553.

9. Lampson BC, Parisi JT (1986) Nucleotide sequence of the constitutive macrolide-lincosamide-streptogramin B resistance plasmid pNE131 from Staphylococcus epidermidis and homologies with Staphylococcus aureus plasmids pE194 and pSN2. J Bacteriol 167: 888-892.

10. Labigne-Roussel A, Courcoux P, Tompkins L (1988) Gene disruption and replacement as a feasible approach for mutagenesis of Campylobacter jejuni. J Bacteriol 170: 1704-1708.

11. Yao R, Alm RA, Trust TJ, Guerry P (1993) Construction of new Campylobacter cloning vectors and a new mutational cat cassette. Gene 130: 127-130.

12. Watson RO, Novik V, Hofreuter D, Lara-Tejero M, Galan JE (2007) A MyD88-deficient mouse model reveals a role for Nramp1 in Campylobacter jejuni infection. Infect Immun 75: 1994-2003.
